# Evolving Highly Active Oxidic Iron(III) Phase from Corrosion of Intermetallic Iron Silicide to Master Efficient Electrocatalytic Water Oxidation and Selective Oxygenation of 5‐Hydroxymethylfurfural

**DOI:** 10.1002/adma.202008823

**Published:** 2021-05-28

**Authors:** J. Niklas Hausmann, Rodrigo Beltrán‐Suito, Stefan Mebs, Viktor Hlukhyy, Thomas F. Fässler, Holger Dau, Matthias Driess, Prashanth W. Menezes

**Affiliations:** ^1^ Department of Chemistry: Metalorganics and Inorganic Materials Technical University of Berlin Straße des 17 Juni 135. Sekr. C2 10623 Berlin Germany; ^2^ Department of Physics Free University of Berlin Arnimallee 14 14195 Berlin Germany; ^3^ Department of Chemistry Technical University of Munich Lichtenbergstraße 4 85747 Garching Germany

**Keywords:** alkaline oxygen evolution reaction, intermetallic compounds, iron, selective oxygenation of organics, silicon, water oxidation

## Abstract

In a green energy economy, electrocatalysis is essential for chemical energy conversion and to produce value added chemicals from regenerative resources. To be widely applicable, an electrocatalyst should comprise the Earth's crust's most abundant elements. The most abundant 3d metal, iron, with its multiple accessible redox states has been manifold applied in chemocatalytic processes. However, due to the low conductivity of Fe^III^O*_x_* H*_y_* phases, its applicability for targeted electrocatalytic oxidation reactions such as water oxidation is still limited. Herein, it is shown that iron incorporated in conductive intermetallic iron silicide (FeSi) can be employed to meet this challenge. In contrast to silicon‐poor iron–silicon alloys, intermetallic FeSi possesses an ordered structure with a peculiar bonding situation including covalent and ionic contributions together with conducting electrons. Using in situ X‐ray absorption and Raman spectroscopy, it could be demonstrated that, under the applied corrosive alkaline conditions, the FeSi partly forms a unique, oxidic iron(III) phase consisting of edge and corner sharing [FeO_6_] octahedra together with oxidized silicon species. This phase is capable of driving the oxyge evolution reaction (OER) at high efficiency under ambient and industrially relevant conditions (500 mA cm^−2^ at 1.50 ± 0.025 V_RHE_ and 65 °C) and to selectively oxygenate 5‐hydroxymethylfurfural (HMF).

## Graphical abstract

The application of iron for oxidizing electrocatalytic reactions is limited due to the low conductivity of Fe^III^O*_x_* H*_y_* phases. Using the second most abundant element of the Earth's crust, silicon, intermetallic FeSi is formed, which is demonstrated to drive the oxygen evolution reaction (OER) at high efficiency under ambient and industrially relevant conditions (500 mA cm^−2^ at 1.50 ± 0.025 V_RHE_ and 65 °C), as well as selectively oxygenate 5‐hydroxymethylfurfural (HMF).

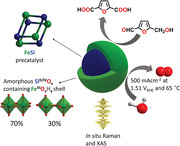

## 1 Introduction

During the last century, chemical energy in form of fuels (mainly oil, gas, and coal) was used to produce the various energy types demanded by society (e.g., electric, kinetic, and thermal).^[^ ^1^, ^2^, ^3^ ^]^ To do so, the electric generator and combustion engine were of particular importance.^[^ ^3^ ^]^ On the contrary, in a future sustainable energy economy, electricity from wind and solar power plants must be utilized and efficiently transformed.^[^ ^4^ ^]^ In such a system, electrocatalysts are of fundamental relevance as with their help electricity can be used to produce value‐added chemicals and converted into chemical energy, which is easily storable and transportable.^[^ ^4^, ^5^ ^]^ Such electrocatalysts are only economically viable if they consist out of cheap, earth‐abundant constituents.^[^ ^2^ ^]^ In this regard, the most abundant elements in the earth crust are oxygen (46%), silicon (28%), aluminum (8.3%), and iron (5.6%), and within them, the 3d metal iron has multiple accessible redox states and has been frequently applied as an electrocatalyst for various processes.^[^ ^6^, ^7^, ^8^, ^9^ ^]^ Besides earth abundance also the energy consumption required to receive an element in a processable form is important. To make Si(0) from Si(IV), high temperatures and electric furnaces are required.^[^ ^10^ ^]^ Nevertheless, this is an industrial established process, and, depending on the purity, the price of elemental silicon is as low as 1–3 US$/kg.^[^ ^11^ ^]^

Under ambient conditions and during most electrocatalytic oxidation processes such as the oxygen evolution reaction (OER), iron forms oxidic phases.^[^ ^8^, ^12^ ^]^ Unfortunately, these Fe^III^O*_x_* H*_y_* phases are poorly conducting compared to nickel‐ and cobalt‐containing ones which impede iron's suitability as an electrocatalyst due to large efficiency losses.^[^ ^8^, ^13^, ^14^ ^]^ To overcome this drawback, iron has been successfully doped into conducting, oxidic matrixes of less abundant 3d metals such as cobalt or nickel.^[^ ^8^, ^15^ ^]^ Here, we successfully apply an alternative and potentially cheaper way to master the conductivity disadvantage of Fe^III^O*_x_* H*_y_*: the utilization of iron incorporated in the intermetallic iron silicide (FeSi) phase and bonded to the second most abundant element on the earth's crust, silicon.^[^ ^16^, ^17^, ^18^ ^]^ In contrast to alloys, intermetallics possess ordered, particular structures, which are different than those of the constituent elements.^[^ ^19^, ^20^ ^]^ These structures result from a peculiar bonding situation with covalent and ionic contributions together with conducting electrons.^[^ ^19^ ^]^ The intermetallic FeSi exhibits a crossover from a low‐temperature nonmagnetic semiconductor with a direct bandgap of 73 meV at 7 K to a high‐temperature paramagnetic metal with a Curie–Weiss‐like susceptibility, due to temperature‐dependent phonon renormalization over a wide temperature range.^[^ ^16^, ^21^, ^22^ ^]^ Moreover, intermetallics have been considered to be suitable as tunable electro(pre)catalyst.^[^ ^20^, ^23^ ^]^ In this regard, several reports refer to intermetallic noble‐metal‐based systems for electrocatalysis with high durability and activity compared to unordered alloys.^[^ ^20^, ^24^ ^]^ Recently, for the OER, non‐noble‐metal‐based intermetallics showed remarkable activities and it was revealed that, despite their inherent thermodynamic stability under ambient conditions, some undergo partial corrosion during the OER evolving active oxidized sites interconnected to the conductive precursor phase.^[^ ^25^, ^26^, ^27^, ^28^, ^29^, ^30^ ^]^ Also an intermetallic nickel silicide has been investigated which showed only moderate OER activities probably due to the absence of highly active iron sites.^[^ ^31^ ^]^

Considering the observation that, during the OER, most of the usually highly corrosion resistant intermetallic materials transform at least partly to MO*_x_* H*_y_* phases (M = Fe, Co, Ni), a central research question is to uncover the nature of the newly formed MO*_x_* H*_y_* phase as it is most likely responsible for the catalytic activity.^[^ ^32^, ^33^ ^]^ Recently, it has been shown that the structure of the precursor (e.g., FeSi) and the transformation conditions (e.g., pH or potential) strongly influence the catalytic and chemical properties of the in situ formed catalytically active MO*_x_* H*_y_* phases.^[^ ^32^ ^]^ These considerations explain why MO*_x_* H*_y_* phases are often more active when they are formed in situ from precursors which contain anions that tend to get oxidized and leach during OER conditions. To understand why a catalyst is active or not, the in situ chemical state must be known and analytical methods must be applied that can at least partly answer the following questions: Is the phase ordered on the long range and/or short range, and does it contain defects? What is the coordination of the metal (e.g., tetrahedral or octahedral)? How are the MO*_x_* units linked (e.g., edge or corner sharing)? How many coordinatively unsaturated sites does the phase contain (e.g., edge sites of layered oxyhydroxides)? What is the in situ oxidation state of the metal? Is the phase doped (e.g., by the partially leached anion)? All these factors can be crucial for the catalytic activity and are relevant for the deduction of structure‐activity relations.^[^ ^32^ ^]^

We now learned that the conductive intermetallic FeSi phase is a suitable precursor to master two economically relevant iron‐based electrocatalytic oxidation reactions: the OER and the selective formation of 2,5‐furandicarboxylic acid (FDCA) from 5‐hydroxymethylfurfural (HMF). The OER is a kinetically demanding four‐electron four‐proton transfer reaction. It is the limiting process in water splitting and is also required for electrocatalytic CO_2_ reduction.^[^ ^34^ ^]^ Water splitting and CO_2_ reduction are the key steps of future green fuel formation, which is a prerequisite for a sustainable energy economy with a constant power supply as demanded by society.^[^ ^5^ ^]^ To perform water splitting efficiently, harsh conditions are necessary which include highly basic or acidic environments and elevated temperature (50–80 °C).^[^ ^35^, ^36^, ^37^ ^]^ These conditions are an ongoing challenge for non‐noble‐metal catalysts that easily degrade.^[^ ^12^, ^38^, ^39^ ^]^ Regarding the selective oxygenation of organic substrates, FDCA is an essential substrate in the polymer industry.^[^ ^26^, ^40^ ^]^ It can be used as a precursor for polyethylene 2,5‐furandicarboxylate and poly(ethylene terephthalate).^[^ ^41^ ^]^ Additionally, it is a suitable substituent for terephthalic acid, an intensively used constituent of various polyesters.^[^ ^42^ ^]^ The chosen substrate, HMF, is a biomass derived resource, and selective electrooxidation of it can be driven by green electricity while avoiding both toxic chemicals and harsh conditions.^[^ ^40^ ^]^ Thus, HMF electrooxidation is a sustainable route to FDCA and resulting green polymers.

As a starting point of our investigation, we observed that, under corrosive alkaline conditions, intermetallic FeSi partly transforms to a unique, oxidic iron(III) phase consisting of edge and corner sharing [FeO_6_] octahedra together with oxidized silicon species. This oxidic phase is still interconnected to the remaining FeSi precursor and, in contrast to ordinary Fe^III^O*_x_* H*_y_* species, sufficiently conducting to drive not only the OER at exceptionally low overpotentials (500 mAm^−2^ at 1.50 ± 0.025 V_RHE_ and 65 °C) but also the selective oxygenation of HMF.

## 2 Results and Discussion

**Figure 1:**
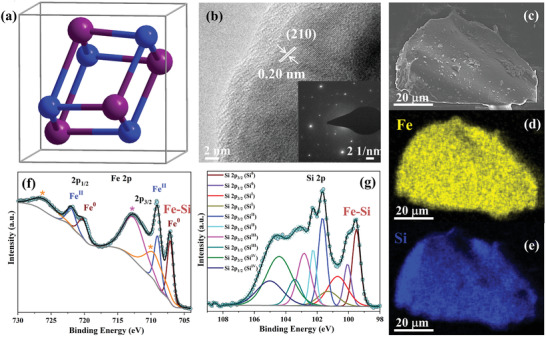
Structural investigation of intermetallic FeSi. a) The crystal structure depicting four Fe (purple spheres) and four silicon (blue spheres) in the unit cell (gray lines). b) HR‐TEM image showing a lattice fringe spacing of 0.20 nm that corresponds to the crystallographic (210) plane. The inserted SAED pattern indicates the high crystallinity of the phase. c) SEM image and d,e) EDX mapping of the particle displaying a homogeneous distribution of Fe (yellow) and Si (blue). The O mapping of the phase is presented in Figure S4, Supporting Information, and the referring EDX spectrum in Figure S5, Supporting Information. f,g) High‐resolution Fe 2p and Si 2p XPS data revealing a slight surface oxidation of the FeSi (* denotes the satellite speaks).

**Figure 2:**
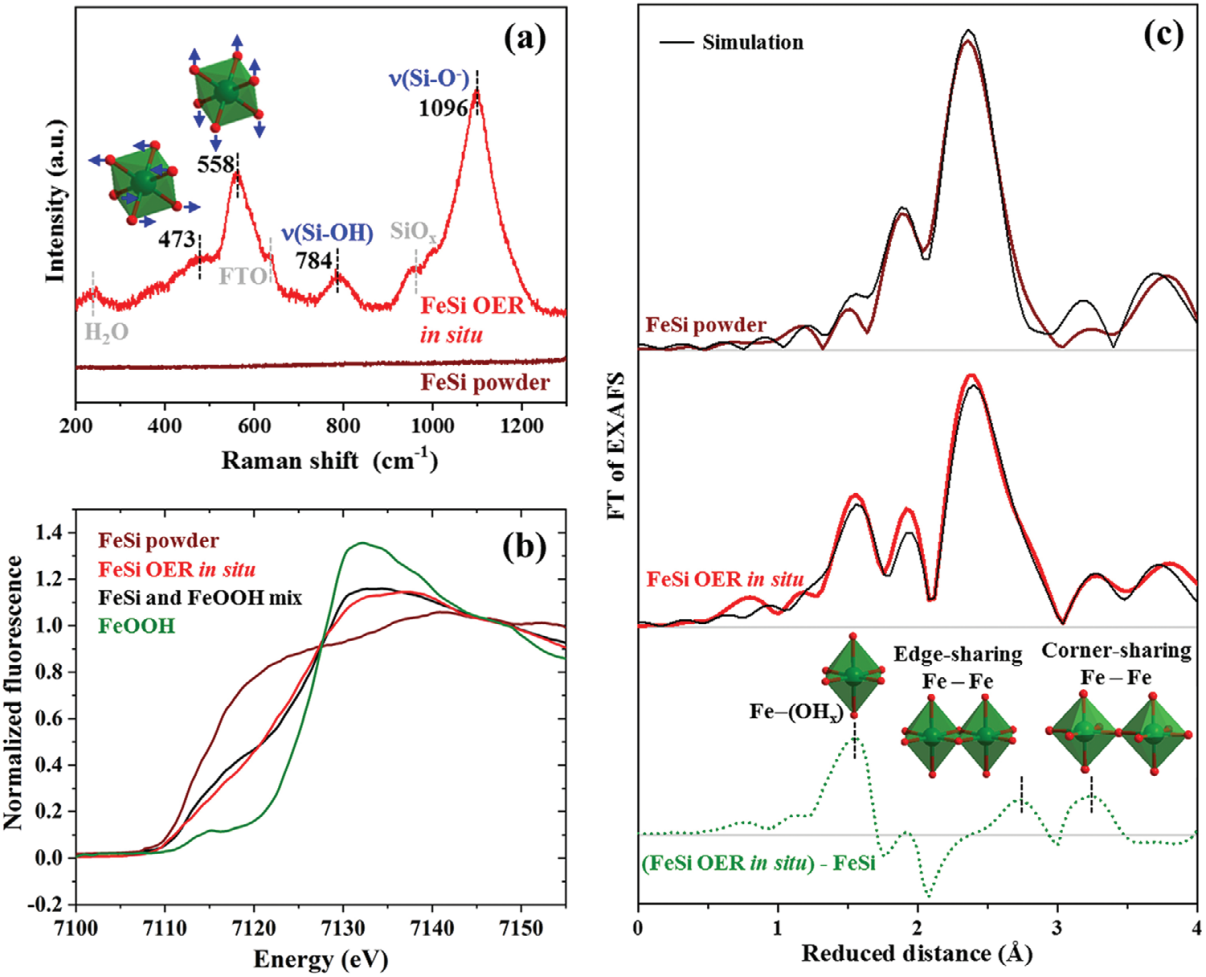
In situ characterization of samples freeze quenched (−196 °C) during OER at 1.65 V_RHE_ after 24 h. The green octahedra are [FeO_6_] units wherein oxygen is represented as red spheres. a) Raman data of pristine FeSi powder and in situ during OER. The numbers represent the frequencies in cm^−1^ of the Raman bands. b) Fe K_α_ XANES spectra. The black line is a linear combination of FeSi powder and FeOOH in a 1 to 1 ratio. c) EXAFS spectra together with their simulations. The green dotted line is the result of the subtraction of the FeSi powder spectrum FT amplitude from the FeSi OER in situ spectrum FT amplitude to gain an EXAFS plot that reflects the newly formed iron(III) phase. The data for the simulations can be found in Tables S8 and S9, Supporting Information.

As a phase‐pure sample, FeSi can be easily synthesized from stoichiometric amounts (1:1) of elemental iron and silicon by arc‐melting. It crystallizes in its own structural type (B20‐type) with the cubic noncentrosymmetric space group *P*2_1_3 (No. 198) and a lattice parameter of *a* = 4.48768(6) Å (**Figure** **1**a and Figure S1, Supporting Information). The unit cell consists of four Fe and four Si atoms and can be described as the distorted NaCl structure.^[^ ^17^, ^18^ ^]^ The phase purity of the obtained material was analyzed by various methods: powder X‐ray diffraction with Rietveld refinement (PXRD, Figure S2, Supporting Information), inductively coupled plasma atomic emission spectroscopy (Table S1, Supporting Information), scanning electron microscopy (SEM) with energy‐dispersive X‐ray (EDX) mapping (Figure 1c–e and Figures S3–S5, Supporting Information), transmission electron microscopy (TEM) with selected‐area electron diffraction (SAED, Figure 1b and Figures S6 and S7, Supporting Information), X‐ray photoelectron spectroscopy (XPS, Figure 1f,g), Raman (**Figure** **2**a), and X‐ray absorption spectroscopy, including X‐ray absorption near edge structure (XANES, Figure 2b) and extended X‐ray absorption fine structure (EXAFS, Figure 2c) analyses. All methods confirm the formation of a pure FeSi phase with a slight surface passivation (Figure 1f,g) as typical for intermetallic compounds.^[^ ^25^, ^26^ ^]^ The obtained particles are irregularly shaped and mostly between 1 and 40 µm large (Figure S3a, Supporting Information).

For the electrochemical investigations of FeSi, we loaded 0.4 ± 0.1 mg on fluorine‐doped tin oxide (FTO) and 0.8 ± 0.1 mg on nickel foam (NF) by electrophoretic deposition, a well‐established binder‐free method for the formation of thin films (see Supporting Information for further information). As iron can leach under strongly oxidizing conditions and redeposit on NiO*_x_* H*_y_*, a contribution of NiFeOOH phases cannot be ruled out for the NF samples.^[^ ^13^, ^43^ ^]^ Therefore, activity experiments and mechanistic assumptions were confirmed on FTO. It should be mentioned here that many iron‐containing reference materials were used on NF to prove that the observed catalytic properties originate from the deposited catalyst. The activity trends of the iron‐containing materials where the same on FTO and NF. The obtained films on FTO and NF show a fully intact FeSi phase (Figures S8–S14, Supporting Information). Furthermore, as no standardized procedure is established in the OER literature and as the testing conditions (mass loading, electrode substrate, testing protocol, etc.) can starkly influence the activity and stability of a catalyst,^[^ ^44^ ^]^ we took great effort to also synthesize and characterize various Co, Ni, FeNi, Ru, Ir, and Pt reference materials (Figures S15–S40, Supporting Information). All these materials were loaded by the same method and with the same loading on FTO and NF, respectively.

**Figure 3:**
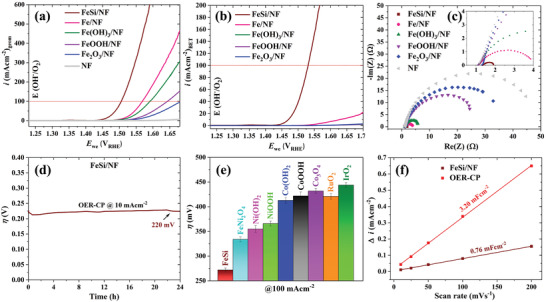
Electrocatalytic OER activities of FeSi and iron‐based references on NF electrodes in 1 m aqueous KOH electrolyte. a) Polarization curves at a scan rate of 1 mV s^−1^. b) The LSVs of (a) normalized by the BET surface. The BET values are 2.1, 27.4, 185.2, 83.1, and 30.1 m^2^ g^−1^ for FeSi, Fe, Fe(OH)_3_, FeOOH, and Fe_2_O_3_, respectively. c) EIS spectra measured at 1.50 V_RHE_. The spectra were fitted (straight lines) using a Randles circuit with a constant phase element (see Table S2, Supporting Information, for fitting values). d) Chronopotentiometry experiment of FeSi/NF conducted at 10 mA cm^−2^. e) Bar diagram (with error bars) displaying OER overpotentials at 100 mA cm^−2^ of various Co‐, Ni‐, FeNi‐, Ru‐, and Ir‐based catalysts. f) *C*_dl_ determination before and after 24 h OER at 10 mA^−2^.

**Figure** **3** shows the electrocatalytic investigations of FeSi on NF (FeSi/NF) and the reference materials. Linear scanning voltammetry (LSV) reveals that the current densities of 10/100/500 mA cm^−2^ could be reached at the overpotentials (η) of 219 ± 3/281 ± 8/354 ± 15 mV which is 60 mV lower than for the most active iron‐based reference material (Figure 3a,b, for diagrams with error bars, see Figures S41 and S42, Supporting Information). Considering the large particle size of FeSi and the resulting low BET surface area compared to the nanoparticle reference compounds, the superior activity of FeSi is even more remarkable (Figure 3b). Further, the activity trend is confirmed by *R*_ct_ values of the Nyquist plots (Figure 3c) obtained by electrochemical impedance spectroscopy (EIS) (see Table S2, Supporting Information, for all fitting values). A current density of 10 mA cm^−2^ could be yielded for 24 h at a steady η of 220 mV (Figure 3d) revealing a good long‐term stability. Further, for FeSi/NF, a Tafel slope of merely 39 mV dec^−1^ was measured (Figure S43, Supporting Information) which is typical for OER active iron sites.^[^ ^13^ ^]^ Figure 3e shows that also the reference catalysts based on Co, Ni, FeNi, Ru, Ir, and Pt required a higher η under identical conditions (Figures S44–S46 and Table S3, Supporting Information). All activity and stability measurements were also performed on FTO and the same trend was obtained (Figures S47–S51 and Table S4, Supporting Information). In Table S5, Supporting Information, an η and stability comparison to previously reported catalysts is presented confirming the excellent electrocatalytic properties of FeSi.

To investigate potential reasons for the superior activity of FeSi/NF, additional characterizations were performed. First, the OER Faradaic efficiency was determined to be relatively high with 96 ± 4% (Table S6, Supporting Information). Further, we measured the double layer capacitance (*C*_dl_) of FeSi and all iron‐based compounds (Figures S52 and S53, Supporting Information). As expected from the large particle size of FeSi, its *C*_dl_ is lower than of all other catalysts in the as deposited state. However, after electrocatalysis, the *C*_dl_ is more than four times larger than before, indicating a surface transformation (Figure 3f). To investigate the electron transport properties, we performed four‐point probe conductivity measurements of the deposited films (Table S7, Supporting Information). These measurements revealed that FeSi before OER is at least two and after 24 h OER still more than one order of magnitude more conducting than the other iron‐based reference materials.

**Figure 4:**
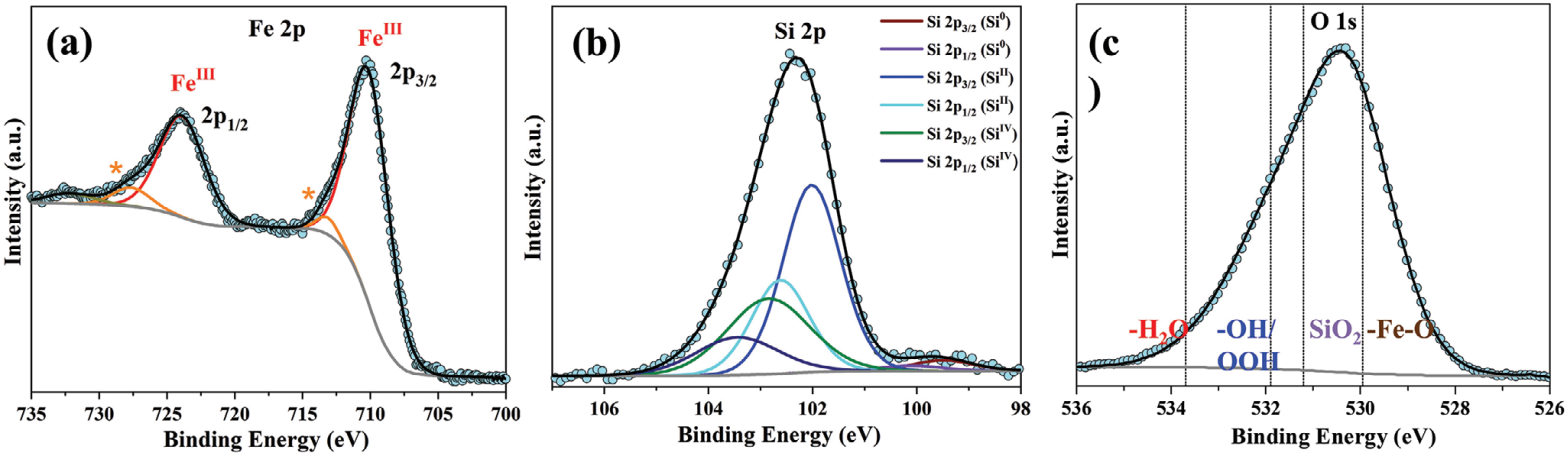
High‐resolution XPS spectra of a FeSi sample after 24 h OER at 10 mA cm^−2^. a) Fe 2p spectrum revealing the presence of Fe^III^ (* denotes the satellite speaks). b) Deconvoluted Si 2p spectrum showing Si^II^ and Si^IV^ with a minute amount of residue Si^0^. c) O 1s spectrum with previously observed binding energies of various oxygen species (dotted lines; see Figure S63, Supporting Information, for the direct comparison of the O 1s spectra before and after OER).^[^ ^50^, ^51^, ^52^ ^]^

The redox potentials for the oxidation of Fe^0^ and Si^0^ are much lower than the one for water oxidation.^[^ ^45^, ^46^ ^]^ Therefore, it is expected that at least a surface modification of the FeSi takes place as also indicated by the larger *C*_dl_ after the OER reaction.^[^ ^12^ ^]^ Additionally, FeSi activates during the OER, as can be seen in the first hours of the CP curves (Figure 3d and Figure S50, Supporting Information). We have also studied this activation by CV (Figure S54b,c, Supporting Information). To understand the structural changes causing the activation, we performed an extensive characterization of FeSi after 24 h at 10 mA cm^−2^. PXRD reveals the presence of crystalline FeSi after OER and no additional new reflections can be observed in the diffractogram (Figure S55, Supporting Information). SEM/EDX investigations uncover an unchanged morphology with an equal distribution of Fe and Si in the particles but the amount of Si is reduced by around 30% (Figures S56–S59, Supporting Information; for the determination of the iron to silicon ratio by various methods, see Table S1, Supporting Information). This ratio was the same when the sample was kept at 100 mA cm^−2^ for 24 h (Table S1, Supporting Information). TEM analysis shows that an amorphous, porous phase has formed on top of the particles and SAED confirms that a crystalline core is present (Figures S60 and S61, Supporting Information). Fourier transform infrared spectroscopy uncovers the emergence of various new bands after the OER (Figure S62, Supporting Information). These bands could be assigned to silicate compounds, interlayer water, and carbon dioxide as well as to FeO*_x_* H*_y_* species.^[^ ^47^, ^48^, ^49^ ^]^ The deconvoluted Fe 2p XPS data after OER shows only Fe^III^ and no Fe^II^ or Fe^0^ anymore (Figures 1f and **Figure** **4**a). Consistently, the Si 2p spectrum reveals the oxidation of Si^0^ to Si^II^ and Si^IV^ during OER (Figure 4b). Additionally, the O 1s spectra before and after OER show the appearance of a pronounced oxygen peak coherent with the formation of silicon oxide, iron oxide, and iron (oxy)hydroxide species (Figure 4c, and Figure S63, Supporting Information).^[^ ^50^, ^51^, ^52^, ^53^ ^]^

To gain more information on the structural transformation of FeSi during the OER, we performed in situ Raman spectroscopy and XAS on samples freeze quenched (−196 °C) during OER conditions at 1.65 V_RHE_ after 24 h.^[^ ^32^, ^54^ ^]^ The pristine FeSi exhibit no Raman active transitions above noise level in the investigated range under our measurement conditions (200–1300 cm^−1^; Figure 2a). The in situ spectrum contains three major bands (Figure 2a). The two bands at 784 and 1096 cm^−1^ are typical for ν(Si—OH) and ν(Si—O^−^) stretching vibrations, respectively, as previously observed in alkaline silicate solutions and the band at 952 cm^−1^ is a typical feature of SiO*_x_* phases.^[^ ^55^ ^]^ The two bands at 473 and 558 cm^−1^ do not fit to typical iron oxyhydroxide phases such as α, β, γ‐FeOOH, or ferrihydrites.^[^ ^56^ ^]^ However, they closely resemble the Raman features observed for nickel‐, cobalt‐, and iron‐containing layered double hydroxides that form during the OER and can be assigned to the δ(Fe—O) (473 cm^−1^) and ν(Fe—O) (558 cm^−1^) vibration modes of edge sharing [FeO_6_] octahedra (insets of Figure 2a).^[^ ^30^, ^57^, ^58^ ^]^ The band at 240 cm^−1^ and the one at around 640 cm^−1^ are most likely caused by the frozen electrolyte and the FTO substrate, respectively.^[^ ^59^, ^60^, ^61^ ^]^ The XANES spectrum of the pristine FeSi powder shows a slow rise in absorption and no edge maximum as expected due to the soft silicon ligands (Figure 2b). The edge of the in situ sample has a steeper rise and could be reproduced by a linear combination of the FeSi and a FeOOH edge in a 1:1 ratio (Figure 2b). Based on crystallographic data, the EXAFS spectrum of pristine FeSi could be successfully simulated (Figure 2c and Figure S64 and Table S8, Supporting Information). For the in situ EXAFS spectrum, in analogy to the XANES modeling, two phases in a 1 to 1 ratio were used for the simulation (Figure 2c and Figure S64 and Table S9, Supporting Information). One is the FeSi phase of the pristine material and the other one is a Fe^III^O*_x_* H*_y_* phase. This Fe^III^ phase is comprised of [FeO_6_] octahedra which are either connected through shared edges (70%) such as in layered double hydroxides or by shared corners (30%, Figure 2c inset, and Table S9, Supporting Information). Recently, Wang et al. successfully used the same approach for the description of various FeO*_x_* H*_y_* phases with low crystallinity.^[^ ^62^ ^]^ A subtraction of the FeSi EXAFS spectrum FT amplitude from the in situ one yields a plot with three peaks (Figure 2c, dotted green line). The three structural motives Fe—O, Fe—Fe edge sharing, and Fe—Fe corner sharing can be assigned to these peaks and their respective distances (green polyhedra, Figure 2c, and Table S9, Supporting Information). No new Fe–Si distance could be identified by our EXAFS analysis.

The FeSi/FTO and FeSi/NF electrodes activate in the first 2–4 h during CP and during the first 20 CVs (Figure 3d and Figures S50 and S54b,c, Supporting Information). After this, the activity remains unaltered; however, the catalyst transformation continues, as we found that only 10% of the silicon leached when the OER activation just finished compared to 30% after 24 h (see Table S1, Supporting Information). Therefore, we conclude that only around one‐third of the newly formed oxidic Fe(III) contributes significantly to the OER and that the Fe(III) forming after the activation is completed does barely participate in the OER.

Summarizing our structural investigations, after 24 h OER, around 50% of the FeSi transformed into a new iron(III) phase. This new phase is structurally disordered (PXRD amorphous) and consists of [FeO_6_] octahedra which are connected via edge (70%) and corner sharing (30%). Even though around 50% of the FeSi transformed, only 30% of the silicon leached. Therefore, the oxidic iron(III) phase still contains 40% of the initial silicon. Surface sensitive XPS and near surface sensitive Raman confirm the presence of oxygenated silicon species. EXAFS cannot identify new Fe–Si distances which indicate that the silicon is inside the structure in an unordered way such as in interlayer spaces or other cavities. From the silicon Pourbaix diagram, H_2_SiO_4_^2−^ is the thermodynamically stable species under OER conditions.^[^ ^46^ ^]^ However, under alkaline conditions, the solution chemistry of silicates is complex and oligomers or even polymers can form.^[^ ^47^, ^55^ ^]^ Further, XPS shows the presence of silicon(II), such species are most likely still bonded to surrounding iron atoms and might not have direct access to the electrolyte which impedes their further oxidation. The newly formed silicon‐containing oxidic iron(III) phase shows a remarkable OER activity especially when the huge particle size (up to 40 µm) and the resulting low BET surface area (Figure 3b and Figure S51, Supporting Information) are considered. The reasons for this superior activity are: i) it contains Fe as the catalytic center (Tafel slope 39 mV dec^−1^) which is considered to be the most active 3d element for OER;^[^ ^14^ ^]^ ii) not only the surface Fe sites are OER active but around one‐third of the newly formed Fe(III) participates probably due to its amorphous nature and the silicon leaching;^[^ ^32^ ^]^ iii) it has a sufficiently high conductivity which is probably caused by its unique structure and the presence of oxidic silicon(II) and (IV) species; iv) the PXRD amorphous structure formed by the partial silicon leaching is disordered and contains more edge sites than crystalline layered phases;^[^ ^63^ ^]^ and v) it contains a conducting FeSi core which could facilitate electron transfer to the OER active iron sites but as the newly formed oxidic Fe(III) phase is comparably large, this effect might not be as decisive as in other nanostructured core–shell particles.^[^ ^26^ ^]^ Interestingly, the structural transformation of FeSi was found to be different to the previously investigated group 14 intermetallic iron‐containing catalyst (see Table S10, Supporting Information, for comparison with FeSn_2_).

**Figure 5:**
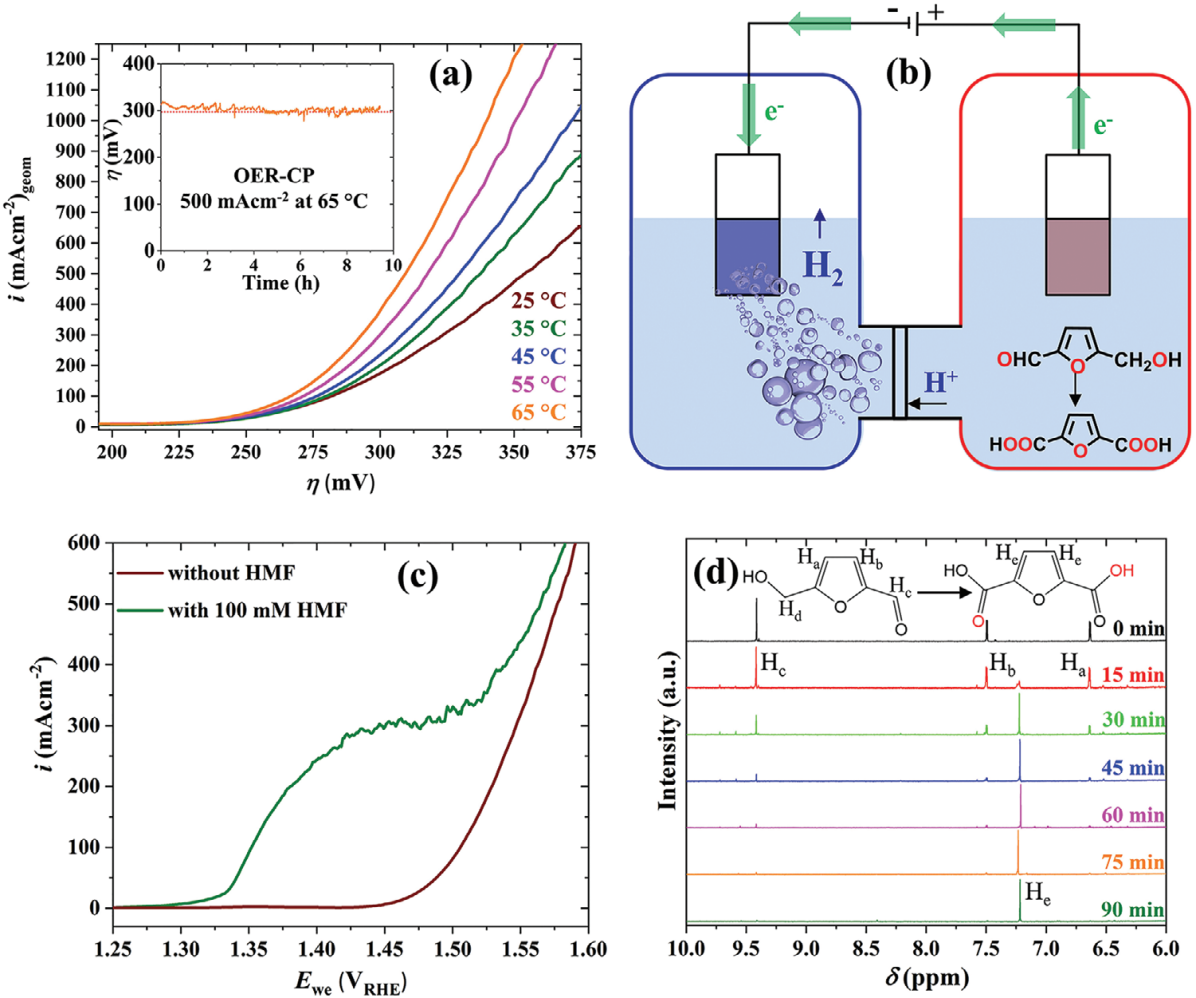
a) LSV of FeSi/NF at different temperatures at a scan rate of 1 mV s^−1^. The inset shows a CP measurement of FeSi/NF at 500 mA cm^−2^ and at 65 °C. b) Anodic HMF oxidation coupled with cathodic hydrogen evolution. c) LSVs of FeSi/NF in 1 m KOH with and without HMF addition. d) ^1^H NMR tracking of the bulk HMF oxidation at 20 mA cm^−2^ in 1 m KOH solution.

After conclusively characterizing our system, we decided to apply it under industrially relevant OER conditions which include elevated temperatures and large current densities.^[^ ^34^, ^35^, ^36^ ^]^ First, we performed LSVs at different temperatures up to 65 °C and could observe a significant enhancement of the OER activity (10/100/500 mA cm^−2^ at 1.408/1.467/1.509 V_RHE_ at 65 °C; **Figure** **5**a and Table S11, Supporting Information). To investigate the stability, we performed a CP measurement at 500 mA cm^−2^ at 65 °C (inset Figure 5a). We want to point out four fundamental aspects that we found neglected in most of the previous reports on OER at elevated temperatures: i) the reversible OER potential (*E*_rev_ in V) is a function of the temperature (*T* in Kelvin) and can be approximated by^[^ ^64^ ^]^

(1) $$\begin{array}{l} E_{\text{rev}}=1.5184-(1.5421\times 10^{-3}\times T\text{) + (}9.523\times 10^{-5}\times T\times \ln T)\\ \text{+ (}9.84\times 10^{-8}\times T^{2}) \end{array}$$

ii) the Nernst equation is also temperature dependent and thus the term 2.303*RT*/*F* (*R* is the ideal gas constant and *F* the Faraday constant) deviates from the 0.059 observed at 25 °C and must be adjusted;^[^ ^65^ ^]^ iii) when the temperature of the reference electrode changes, also the reference potential changes which must be considered depending on the used reference electrode (e.g., for Hg/HgO in 1 m NaOH, we fitted [deviation below 2 mV between 25–90 °C] experimental data from Rondinini et al.:^[^ ^66^ ^]^ *E*_Hg/HgO_ **= 93.23 mV* *+* *1.092*T *mV K^−1^ **−* *0.003613*T* ^2^ mV K^−^^2^); iv) the pH value of 1 m KOH solution is a function of temperature (e.g., 13.15 at 40 °C).^[^ ^67^ ^]^ Due to these various factors, it is most suitable to calibrate the reference electrode with a RHE at each temperature in 1 m KOH as it has been done for this work.^[^ ^68^ ^]^ For FeSi at 65 °C, the potential versus RHE to reach 10 mA cm^−^² is reduced by around 50 mV compared to 25 °C leading to an improved efficiency in respect to the heating values of the produced gases (Table S11, Supporting Information). This reduction can be partly (30 mV) explained by the intrinsic change of the thermodynamics of the OER (*E*_rev_ = 1.196 V at 65 °C; Table S11, Supporting Information); the remaining 20 mV overpotential reduction is most likely of kinetic origin as described in the Arrhenius equation.^[^ ^37^, ^69^ ^]^ Furthermore, at 500 mA cm^−^² the potential versus RHE reduces by 80 mV. At this current density, additionally, mass transport effects like diffusion or gas bubble detachment could be relevant and facilitated by the increased temperature.

Additional to the OER, we choose to apply our cheap, earth‐abundant catalyst for the economically viable, selective oxidation of HMF to FDCA in 1 m KOH (see Scheme S1, Supporting Information, for a reaction pathway).^[^ ^70^, ^71^ ^]^ This oxidation process can be combined with the hydrogen evolution reaction leading to the formation of two value‐added products in one electrochemical cell: H_2_ at the cathode and FDCA and the anode (Figure 5b).^[^ ^26^ ^]^ For the HMF oxidation, we used FeSi electrodes that were first exposed to OER conditions to form the active oxidic iron(III) phase. With these electrodes, we investigated whether the addition of HMF affects the current response in an LSV (Figure 5c). We found that in the presence of HMF, an oxidation takes place prior to the OER onset which substantiates the capability of FeSi to selectively oxidize HMF. This result is consistent with the observation that OER catalysts can oxygenate alcohols in alkaline solution by forming electrophilic *OH species through the adsorption of OH^−^.^[^ ^72^ ^]^ Motivated by this result, we performed a bulk electrolysis of HMF at 20 mA cm^−2^ in 1 m KOH with Pt wire as hydrogen evolving cathode. The oxidation of HMF was tracked by ^1^H NMR spectroscopy every 15 min (Figure 5d). After 90 min, only FDCA and no residual HMF could be found (Figure 5d and Figure S65, Supporting Information). Only trace amounts of impurities could be observed after around 90 min, when a charge of 103.96 C was passed by the potentiostat, which yields a Faradaic efficiency of 94 ± 3% (see Supporting Information for details) revealing the potential suitability of FeSi for the selective HMF oxygenation to FDCA. During CA experiments, we found that the current density is barely affected when the potential is changed in the range of 1.36–1.425 V_RHE_. The most important criteria to obtain high oxygenation rates are the substrate concentration, the electrode surface area, and the stirring speed indicating that HMF mass transport is the limiting phenomenon.

## 3 Conclusion

Here, we have introduced a cheap approach for the formation of highly effective electrocatalysts based on some of the Earth‐crust's most abundant elements: O, Fe, and Si. We have successfully demonstrated the application of our system for economically viable processes. Further, the extensive in and ex situ characterization provides conceptual insights reaching beyond the class of materials investigated herein. We believe that further improvements in catalytic activity could be achieved by the synthesis of highly dispersed FeSi nanoparticles through various approaches other than high‐temperature solid‐state methods. In this regard, our future work will focus on low‐temperature molecular methods such as the single‐source precursor approach.^[^ ^73^ ^]^

## Conflict of Interest

The authors declare no conflict of interest.

## Supporting information

Supporting Information

## Data Availability

Research data are not shared.
