# Supplementary material for: Evolving Highly Active Oxidic Iron(III) Phase from Corrosion of Intermetallic Iron Silicide to Master Efficient Electrocatalytic Water Oxidation and Selective Oxygenation of 5‐Hydroxymethylfurfural
Source: Adv Mater. 2021 May 28;33(27):2008823. doi: 10.1002/adma.202008823 (PMC11468827; doi:10.1002/adma.202008823)
Supplement: Supplementary file 1 — Supporting Information [file ADMA-33-2008823-s001.pdf]

© 2021 Wiley-VCH GmbH

ADVANCED MATERIALS

Supporting Information

for *Adv. Mater.,* DOI: 10.1002/adma.202008823

Evolving Highly Active Oxidic Iron(III) Phase from Corrosion of Intermetallic Iron Silicide to Master Efficient Electrocatalytic Water Oxidation and Selective Oxygenation of 5-Hydroxymethylfurfural

J. Niklas Hausmann, Rodrigo Beltrán-Suito, Stefan Mebs, Viktor Hlukhyy, Thomas F. Fässler, Holger Dau, Matthias Driess, and Prashanth W. Menezes**

Copyright WILEY-VCH Verlag GmbH & Co. KGaA, 69469 Weinheim, Germany, 2018.

Supporting Information

Evolving Highly Active Oxidic Iron(III) Phase From Corrosion of Intermetallic Iron Silicide to Master Efficient Electrocatalytic Water Oxidation and Selective Oxygenation of 5-Hydroxymethylfurfural (HMF)

J. Niklas Hausmann, Rodrigo Beltrán-Suito, Stefan Mebs, Viktor Hlukhyy, Thomas F. Fässler, Holger Dau, Matthias Driess, Prashanth W. Menezes**

J. Niklas Hausmann, Rodrigo Beltrán-Suito, Prof. Dr. Matthias Driess, Dr. Prashanth W. Menezes
Department of Chemistry: Metalorganics and Inorganic Materials, Technische Universität Berlin, Straße des 17 Juni 135, Sekr. C2, 10623 Berlin, Germany.
*E-mail: matthias.driess@tu-berlin.de
` prashanth.menezes@mailbox.tu-berlin.de

Dr. Stefan Mebs, Prof. Dr. Holger Dau
Fachbereich Physik, Freie Universität Berlin, Arnimallee 14, 14195 Berlin, Germany.

Dr. Viktor Hlukhyy, Prof. Dr. Thomas F. Fässler
Department Chemie, Technische Universität München, Lichtenbergstraße 4, 85747 Garching, Germany

Table of Contents

Contents	Page number
1. Chemicals and Materials	3
2. Instrumentations	3-6
3. Synthesis of the catalysts	7-8
4. Electrophoretic deposition on NF and FTO	9
5. Electrochemical measurements	9-11
6. Characterization of as-prepared FeSi	12-17
7. Characterization of as-deposited FeSi/FTO	18-21
8. Characterization of as-deposited FeSi/NF	22-24
9. Characterization of Fe-references	25-34
10. Characterization of noble and non-noble metal-based materials	35-38
11. Electrochemical OER activity on NF	38-41
12. Electrochemical OER activity of FTO	43-45
13. Comparison of OER activity of FeSi with FeNi-based catalysts	46
14. Electrochemical characterization of NF	47-50
15. Four-point probe resistivity of Fe-based materials	51
16. *Ex situ* post-characterization after OER-CP (24 h)	52-60
17. *Quasi in situ* XAS spectroscopy	61-63
18. Comparison to $FeSn_2$	64-65
19. Overpotential of FeSi at various temperatures	66
20. Electrochemical oxygenation of HMF	67
21. References	67-69

Chemicals and Materials

All chemical reagents (analytical grade) were used as received without any further purification. Deionized water was used to carry out all the experiments. 1 M aqueous potassium hydroxide (KOH; Fe<0.05 ppm determined by ICP-AES), iron(III) chloride, nickel nitrate, ($Ni(NO_3)_2.6H_2O$), cobalt nitrate ($Co(NO_3)_3$), ferric nitrate ($FeNO_3.xH_2O$), nickel (II) acetate tetrahydrate ($Ni(CH_3COO)_2·4H_2O$), cobalt(II) acetate tetrahydrate ($Co(CH_3COO)_2·4H_2O$), sodium borohydride (NaBH4), cetyltrimethylammonium bromide (CTAB), ammonium oxalate ($(NH_4)_2C_2O_4·2H_2O$), and 20% Pt on Vulcan XC72 were obtained from Sigma Aldrich whereas commercial ruthenium oxide (RuO_2; 99%), iridium oxide (IrO_2; 99%), cobalt oxide (Co_3O_4), iron granules (99.98 %), silicon pieces (99.999 %) and 5-Hydroxymethyl-2-furaldehyde (97%) were purchased from Alfa Aesar. The electrode substrate nickel foam (NF) and fluorine-doped tin oxide (FTO, resistivity 8−12 Ω/sq) were obtained from Recemat BV and Sigma Aldrich, respectively.

Instrumentations

X-ray diffraction

Powder X-ray diffraction (PXRD) patterns were measured on a Bruker AXS D8 advanced automatic diffractometer equipped with a position-sensitive detector (PSD) and curved germanium (111) primary monochromator using Cu-$K_{\alpha 1}$ radiation (λ = 1.5418 Å). The structural models of as-prepared products were constructed using the DIAMOND program version 3.0. The PXRD of the FTO deposited electrodes were measured using Bragg-Brentano geometry under air, using a Rigaku *SmartLab 3 kW* diffractometer (Rigaku Corporation, Japan) with Cu-$K_{\alpha 1}$ (λ = 1.5418 Å) radiation. Data acquisition was carried out using the *SmartLab Guidance* software package (Rigaku Corporation, Japan; Version 2.1.0.0.

The sample of intermetallic FeSi was characterized with powder X-ray diffraction (PXRD) on a STOE Stadi-P with Cu-$K_{\alpha 1}$ source (λ = 1.54058 Å), curved Ge(111)-monochromator and DECTRIS MYTHEN 1K detector. Phase analysis was performed using the structure model from ICSD-database. Rietveld analysis has been done with the Fullprof Suite (Figure S2). Peak profile shape was described by a pseudo-Voigt function; the background of the diffraction pattern was fitted using a linear interpolation between selected data points in non-overlapping regions. Scale factor, lattice parameters, fractional coordinates of atomic sites and their isotropic displacement parameters, profile shape parameters, and half-width (Caglioti) parameters were varied during the Rietveld refinement.

Elemental analyses

Inductively coupled plasma atomic emission spectroscopy (ICP-AES) was carried out on a Thermo Jarrell Ash Trace Scan analyzer. The presented materials were digested in aqua regia HCl:HNO_3 3:1 v/v (nitric acid, SUPRA-Qualität ROTIPURAN® Supra 69%, and hydrochloric acid, SUPRA-Qualität ROTIPURAN® Supra 30%), and the average of three reproducible independent experiments were presented. The digestion volume (2.5 mL) was diluted with Milli-Q water up to 15 mL. For the post-OER investigations, the electrolyte solutions were analyzed as obtained. Calibration curves were prepared for both iron and silicon with

concentrations between 1 mgL^{-1} and 100 mgL^{-1} from standard solutions (1000 mgL^{-1} single-element ICP-Standard Solution ROTI®STAR).

Electron microscopy

To gather information on the morphology, and the surface structures, scanning electron microscopy (SEM) was performed on an LEO DSM 982 microscope integrated with EDX (EDAX, Apollo XPP). Data handling and analyses were attained with the software package EDAX.

The microstructure investigations of the materials were achieved by transmission electron microscopy (TEM), which was explored on an FEI Tecnai G2 20 S-TWIN transmission electron microscope (FEI Company, Eindhoven, Netherlands) equipped with a LaB_6 source at 200 kV acceleration voltage. For the analysis of the films after electrocatalysis, the films were scraped from the electrode substrate and transferred onto a carbon-coated copper grid. EDX analyses were achieved with an EDAX r-TEM SUTW detector (Si (Li) detector), and the images were recorded with a GATAN MS794 P CCD camera. The SEM and TEM experiments were conducted at the Zentrum für Elektronenmikroskopie (ZELMI) of the TU Berlin.

Fourier-transform infrared spectroscopy (FT-IR)

FT-IR was examined using a BIORAD FTS 6000 FT-IR spectrometer under attenuated total reflection (ATR) conditions. The data were recorded in the range of 500–4000 cm^{-1} with an average of 32 scans at 4 cm^{-1} resolution. For investigations after OER-CP, the films were scratched off from the FTO substrate.

Brunauer–Emmett–Teller (BET) specific surface area

The BET surface area surface areas of the as-prepared materials were determined on a Quantachrome Autosorb-1 apparatus. Nitrogen adsorption/desorption isotherms were measured at -196˚C after degassing the sample at 120˚C overnight. The BET surface areas (S_{BET}) were calculated from the adsorption data in a relative pressure ranging from 0.01 to 0.1.

Gas chromatography (GC) for Faradaic efficiency (FE)

Gas chromatography (GC) was used to calculate the FE of the oxygen evolution reaction (OER) that was performed in a closed (gas-tight) electrochemical cell. An Agilent 7890A gas chromatograph was used to determine the oxygen content in the headspace of the electrochemical cell. The gas chromatograph was furnished with a carboxen-1000 column and a thermal conductivity detector (TCD). The carrier gas was argon (Ar).

Four-point probe resistivity measurements

A Signature Pro4 System measured the resistivity with Keithley 2400 source-measure unit (SP4-40045TBY) using a four-point probe resistivity technique. The spacing between tungsten carbide tips was 1.016 mm with a radius of 0.245 mm, and a spring pressure was 45 grams. High loadings (2 mg/cm^2) of the materials were electrophoretically deposited on FTO electrodes to estimate the specific resistivity of each synthesized material, and the average results are presented.

X-ray photoelectron spectroscopy (XPS)

The XPS measurements were carried out on a Kratos Axis Ultra X-ray photoelectron spectrometer (Kratos Analytical Ltd., Manchester, U.K.) using an Al K_{α} monochromatic radiation source (1486.7 eV) with 90° takeoff angle (normal to analyzer). The vacuum pressure in the analyzing chamber was kept at 2×10^{-9} Torr. The XPS spectra were collected for C 1s, O 1s, Ni 2p, and Ge 3d levels with pass energy 20 eV and step 0.1 eV. The binding energies were calibrated relative to the C 1s peak energy position as 285.0 eV. Data analyses were carried out using Casa XPS (Casa Software Ltd.) and the Vision data processing program (Kratos Analytical Ltd.).

Resonance Raman (RR) spectroscopy

Quasi in situ and *ex situ* RR spectra were recorded using the 458 nm emission of an Argon ion laser (Innova 70, Coherent) for excitation and a confocal Raman spectrometer (Lab Ram HR-800 Jobin Yvon) equipped with a liquid-nitrogen cooled charge-coupled device (CCD) camera for data acquisition. The typical laser power at the sample was 0.5 mW. Measurements were performed using a Linkam Cryostage THMS600 cryostat. The temperature of the films was kept at 80 K throughout the measurements. The measurements were conducted at three different parts of the film and were consistent with the attained peak positions. For the *quasi in situ* experiments, the required current potential for 10 $mAcm^{-2}$ (1.65 $V_{RH,}$ CA, 24 h) was applied in analogy to the electrochemical experiments, and the films were freeze-quenched using liquid N_2 under vigorous Ar gas flow and stored in liquid N_2.

Quasi *in situ* X-ray absorption spectroscopy (XAS)

To get insights on the local atomic and electronic structure extended X-ray absorption fine-structure (EXAFS), and near-edge structure (XANES) studies were carried out under *quasi in situ* conditions. The XAS spectra (XANES/EXAFS) were recorded at the BESSY II synchrotron radiation source operated by the Helmholtz-Zentrum Berlin. The measurements were performed at the KMC-3 bending-magnet beamline at 20 K in a helium-flow cryostat (Oxford-Danfysik). The incident beam energy was selected by a Si(111) double-crystal monochromator. The measurements at the iron K-edge were performed in fluorescence mode (K[illegible] fluorescence energy region selected) using a 13 element silicon drift detector (Rayspec). Over 20 spectra were averaged for each compound in order to improve the signal-to-noise ratio. Averaged spectra were background-corrected and normalized using in-house software. The extracted spectra were weighted by k^3 and simulated in k-space (E_0 = 7112 eV). The XANES edge position was determined by the integration method.[2] All EXAFS simulations were performed using in-house software (SimXLite, programmed by Dr. Petko Chernev) after calculation of the phase functions with the FEFF program (version 8.4, self-consistent field option activated).[3] The data range used in the simulation of the EXAFS spectra was k = (2-14) $Å^{-1}$. The EXAFS simulation was optimized by a minimization of the gg sum obtained by the summation of the squared deviations between measured and simulated values (least-squares fit). The fit was performed using the Levenberg-Marquardt method with numerical derivatives. The error ranges of the fit parameters were estimated from the covariance matrix of the fit. Cosine windows covering 10% of the low k-side and 10% of the high k-side of the spectra were applied for the calculation of the Fourier transforms. Further details are given elsewhere.[1,4]

FeSi samples for XAS experiments were prepared on FTO substrates in analogy to the electrochemical experiments. The samples were electrochemically treated in OER chronoamperometric conditions (1.65 V_{RHE}, CA, 24 h) at a potential yielding a current density of around 10 $mAcm^{-2}$ in aqueous 1 M KOH solution. During the electrochemical measurement, the samples were freeze-quenched using liquid N_2 under vigorous argon gas flow and stored in liquid N_2 until XAS measurements were conducted.

Synthesis of the catalysts

Synthesis of intermetallic FeSi

Starting materials for the syntheses of FeSi were commercially available elements with high purity: iron granules (Alfa Aesar, 99.98 %) and silicon pieces (Alfa Aesar, 99.999 %). Stoichiometric amounts of the iron and silicon (1:1) were melted in an arc-furnace (Mini Arc Melting System, MAM-1, Johanna Otto GmbH) placed in an argon-filled glovebox (MBraun 20 G, argon purity 99.996 %). The pellet has been flipped over and remelted three times to ensure homogeneity. The resulting pellet was ground with an agate mortar.

Synthesis of Fe-nanoparticles [5]

Iron (Fe) nanoparticles were prepared by reduction of iron(III) chloride following a reported procedure. Typically, $FeCl_3.6H_2O$ (0.27 g, 1 mmol) was added to a solution of polyethylene glycol (PEG 4000, 2 g) in 110 mL of ethylene glycol and the mixture was stirred vigorously in a 3 necked round bottom flask at about 150 ºC under a rapid and constant flow of N_2. After complete dissolution of the solid, the temperature was gradually increased to about 170 ºC under reflux and the hot well-stirred mixture was reduced by a slow addition (through septum) of a suspension of $NaBH_4$ (0.7 g, 18.4 mmol) in ethanol (~10 mL) over a period of 15-20 minute. The reflux under steady N_2 flow was continued for an additional 30 minutes and thereafter the reaction mixture was allowed to cool down to room temperature. The solid was separated by ultracentrifugation and was washed thoroughly with ethanol and finally with acetone and dried at 60 ºC (yield was about 75%).

Synthesis of $Fe(OH)_3$[6]

Iron(III) hydroxide has been prepared by the precipitation method. To an aqueous solution of ferric nitrate ($FeNO_3.xH_2O$; 205 mg, 0.507 mmol), three equivalents of KOH (1.6 mmol; 1.6 mL of 1 M KOH) was added dropwise while stirring the solution vigorously at room temperature. After an hour of constant stirring, the brown precipitate was isolated by centrifugation, and washed thoroughly with water, and dried at 60ºC overnight in air.[2]

Synthesis of FeOOH[6]

500 mg of $FeSO_4.7H_2O$ (Merck Millipore) (1.80 mmol) was dissolved on 10 mL of deionized water. 4 mL of 1 M NaOH (4 mmol) was added dropwise and stirred for 30 min at room temperature. The dark brown suspension was then heated gently up to 45 °C and 2 mL of 30% H_2O_2 solution was then added dropwise. The resulted orange suspension was allowed to stir at the same temperature for another 18 h. The final brown precipitate was centrifuged out and washed with deionized water three times, then dried at 60 °C overnight in air.

Synthesis of Fe_2O_3

The iron oxide (Fe_2O_3) nanoparticles were purchased from Sigma Aldrich and used directly for the characterization and the preparation of electrodes.

Synthesis of $Co(OH)_2$[6]

50 mL of 0.1 mol L^{-1} NaOH solution was added dropwise into 80 mL of 0.05 mol L^{-1} $Co(NO_3)_3$ solution. The solution was stirred and maintained at 45 °C for two hours which forms a pink precipitate, which was then washed with deionized water (3×50 mL) and dried in air at 60 ºC.

Synthesis of CoOOH[6]

The as-synthesized $Co(OH)_2$ was dispersed in 30 mL 4 M KOH solution which was then slowly heated up to 45 ºC. To this, 2 mL of 30% H_2O_2 solution was then added dropwise and was kept at the same temperature for 18 h. The final brown precipitate was filtered and washed with deionized water three times (3×50 mL), then dried at 60 °C overnight in air.

Synthesis of Co_3O_4[7]

Micro-emulsions containing cetyltrimethylammonium bromide (CTAB, 2.0 g) as a surfactant, 1-hexanol (20 mL) as co-surfactant, and hexane (35 mL) as the lipophilic phase were synthesized and were mixed separately with an aqueous solution of 0.1 M $Co(CH_3COO)_2{\cdot}4H_2O$ and $(NH_4)_2C_2O_4{\cdot}2H_2O$. Both micro-emulsions were mixed slowly and stirred overnight. The white precipitate then obtained was then centrifuged and washed with 1:1 mixture of chloroform and methanol (200 mL) and subsequently dried at 60 ˚C for 12 hours to form cobalt oxalate ($CoC_2O_4{\cdot}2H_2O$). The as-prepared $CoC_2O_4{\cdot}2H_2O$ precursor was taken in an alumina crucible and heated in dry synthetic air (20% O_2, 80% N_2) at 400 ˚C for 8 hours (2 ˚C/min) and cooled down naturally to ambient temperature to form pure Co_3O_4 phase.

Synthesis of $Ni(OH)_2$[8]

291 mg of $Ni(NO_3)_2.6H_2O$ was dissolved in 10 mL of deionized water and to this solution, 15 mL of NaOH (0.1 M) was added dropwise and stirred continuously for 30min. A bright green solid product was separated, which was then washed and centrifuged thrice with deionized water (3×50 mL), once with acetone (1×50 mL), before drying at 60 °C overnight in air.

Synthesis of NiOOH[6]

The as-prepared $Ni(OH)_2$ was dispersed in 30 mL 4 M KOH solution which was then heated slowly up to 45 °C. An excess of $K_2S_2O_8$ was then added and maintained at the same temperature for 18 h, until the complete change of color to black was obtained. The black solid was washed and centrifuged three times with deionized (3×50 mL), once with acetone (1×50 mL), then dried at 120 °C overnight in air.

Synthesis of $FeNi_2O_4$[9]

1.988 g $FeCl_2{\cdot}4H_2O$ and 4.977 g $Ni(OAc)_2.4H_2O$ were dissolved in a minimum amount of ethanol and stirred for 30 minutes. The solvent was then evaporated at room temperature to obtain the dried powder that was calcinated overnight at 600 °C in oxygen to yield $FeNi_2O_4$.

Electrophoretic deposition (EPD) on substrates

The investigated materials were deposited on both, NF and FTO, electrophoretically, by applying a potential difference of 10 V in a mixture of iodine and acetone on a 1 × 1 cm^2 area. The detailed mechanism involving electrophoretic deposition has been described elsewhere.[10] The electric charge on the catalyst in acetone is insufficient for EPD as very small amounts of free ions exist in acetone, and therefore, large potentials are required for EPD.[10] When iodine is used as the dispersant, it can react with acetone through the keto-enol tautomerism to produce protons through the following equation.

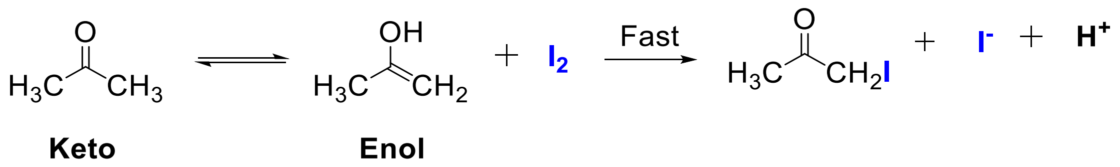

The released protons are adsorbed on the surface of the suspended particles by making them positively charged. The applied electric field induces the positively charged particles to migrate towards and deposit on the cathode.[11] For the typical deposition protocol, 30 mg of the catalyst powder was suspended in 10 ml acetone, and 3 mg of iodine was then added. This solution was agitated in an ultrasonic bath for 30 min. Before EPD, the empty electrodes were weighed using an analytical balance, and the weights were noted. Thin uniform films were achieved by applying a potential of 10 V for 30-180 s by stirring the solution continuously at room temperature. After each EPD, the increase in weight of the electrodes was monitored carefully. For each catalyst, the EPD time was adjusted that a loading of ~0.8 $mgcm^{-2}$ and ~0.4 $mgcm^{-2}$ on NF and FTO was obtained, respectively. The mass loading was reproducible within the margins of an experimental error (±0.1 mg).

Electrochemical measurements

A typical electrocatalytic run was carried out in a standard three-electrode (working, counter, and reference) electrochemical cell in 1 M aqueous KOH (40 ml) with a potentiostat (SP-200, BioLogic Science Instruments) controlled by the EC-Lab v10.20 software package. The electrodes (NF/FTO) with deposited catalysts served as the working electrodes, Pt wire (0.5 mm diameter × 230 mm length; A-002234, BioLogic) as a counter, and Hg/HgO as the reference electrode (CH Instruments, Inc.).

Cyclic voltammetry (CV), linear sweep voltammetry (LSV), chronoamperometric (CA), and chronopotentiometric (CP) experiments were carried out with an applied *iR* compensation of 85%. To do so, prior to the measurements, the uncompensated resistance (R_u) was determined by impedance spectroscopy at 100 MHz and at a potential of 1.175 V_{RHE} with an amplitude of 10 mV. The obtained values were in the range of 9-14 Ω for FTO and 1.0-1.4 Ω for NF. The potential was then corrected by 85% of R_u by the potentiostat during the measurement. The electrocatalytic measurements were performed at least thrice to obtain reliable data and we have presented the error bars wherever necessary. The potentials presented in this work were referenced to the reversible hydrogen electrode (V_{RHE}) in 1 M aqueous KOH (pH = 13.8) using:

$$E(\mathrm{RHE}) = E(\mathrm{Hg/HgO}) + 0.098\ \mathrm{V} + (0.000198 \times T \times \mathrm{pH})\ \mathrm{V\ K^{-1}}.$$

The steady-state Tafel plots were determined by potentiostatic measurements employing stepwise changes of the potential by 15 mV. The current density at each potential was measured for a constant potential for 300 s and the average current values were used for the determination of Tafel plots. The Tafel slope was calculated according to the Tafel equation

$$\eta = b\log i + a$$

where η is the overpotential, b is the Tafel slope, i is the current density, and a is a variable proportional to the logarithm of the exchange current density.[12]

The double-layer capacitances (C_{dl}) of the catalyst films were obtained from their CV (cycled between 0.85 and 0.95 V_{RHE}) at a potential range, where no apparent Faradaic process occurred.[13] Half of the potential difference at 0.90 V_{RHE} were plotted as a function of the scan rate and from the slope, the double layer capacitance C_{dl} was attained.

Electrochemical impedance spectroscopy (EIS) was recorded at 1.55 V_{RHE} for FTO and 1.5 V_{RHE} for NF to obtain the Nyquist plots. The amplitude of the sinusoidal wave was determined in a frequency range of 100 kHz to 1 mHz. The plots were fitted using a simple Randles circuit with a constant phase element instead of an ideal capacitor.[14]

The OER experiments at elevated temperatures were performed in a temperature-controlled closed cell. Around 1.5 cm of the 12 cm long Hg/HgO reference electrode was inside the electrolyte. The rest remained outside at room temperature. For all temperatures, the potential of the Hg/HgO reference electrode was checked against a reversible hydrogen electrode constructed with Pt wire as counter and working electrode and the Hg/HgO electrode as the reference electrode. The RHE potential was determined by the intersection (i = 0 mA/cm^2) of CVs performed with a scan rate of 1 mV/s. It is important that the inside temperature of the Hg/HgO electrode during the calibration vs the RHE and during the electrochemistry is checked and in the best case, it is the same. Otherwise, a correction must be performed which could be done using $E_{Hg/HgO}$ = 93.23 mV + 1.092T mV/K - 0.003613T^2 mV/K^2, as mentioned in the main text.

Bulk electrolysis of 5-hydroxymethylfurfural (HMF)

The LSV investigations were performed in a three-electrode setup analogously to the one of the OER studies. The experiments to determine the Faradaic efficiency were carried out in a two-electrode cell setup, in which the cathode and anode compartments are separated by porous g-4 glass frit. A NF with electrophoretically deposited FeSi (0.5 x 2 cm^2) was used as the anode and a platinum coil as the cathode. Both compartments were filled with 4 mL of 1 M KOH solution and to the anode compartment, 21.9 mg HMF were added. Then a CP experiment with 20 mA was started.

^{1}H NMR (Nuclear Magnetic Resonance) analysis of the organic compound

The reaction mixture was analyzed by ^{1}H NMR spectroscopy in a Bruker AV500 instrument. An ^{1}H NMR sample was prepared by taking a 100 µL aliquot of the reaction solution from the working electrode compartment, 8.25 µl 10 M HCL to neutralize the mixture, and 350 µL D_2O. Processing and plotting of the spectra was performed using MestReNova. A sharp peak at 4.7 ppm appeared for the H_2O from the aqueous reaction mixture and was used as a reference for the chemical shifts of the other proton signals.

Calculation of the Faradaic efficiency for the HMF Oxidation

The Faradaic efficiency was calculated based on the weight of the HMF and the charge passed by the potentiostat. The point of full conversion was determined by ^{1}H NMR spectroscopy when exclusively peaks belonging to 2,5-furandicarboxylic acid (FDCA) could be observed.
The required charge for full conversion ($Q_{100\%\ \mathrm{FE}}$) was calculated using:

$$Q_{100\%\ \mathrm{FE}} = \frac{m_{\mathrm{HMF}} \times F \times n_e}{M_{\mathrm{HMF}}}$$

where F is the Faraday constant (96 485 C mol^{-1}), n_e is the number of electrons required for the oxidation process (6), m_{HMF} is the weight of HMF used for the reaction (21.9 mg) and M_{HMF} is the molecular weight of HMF (126.1 g mol^{-1}). This gives $Q_{100\ \%\ \mathrm{FE}}$ = 100.54 C. When the purity of the HMF (97% Alfa Aesar) is considered this reduces to 97.52 C. After 90 min, ^{1}H NMR spectroscopy revealed only trace amounts of other organic substances than FDCA. At this time, the potentiostat measured a passed charge of 103.96 C. The quotient of these charges yields a Faradaic efficiency of 94% after 90 min. The error of ±3% is based on the inaccuracy of NMR.

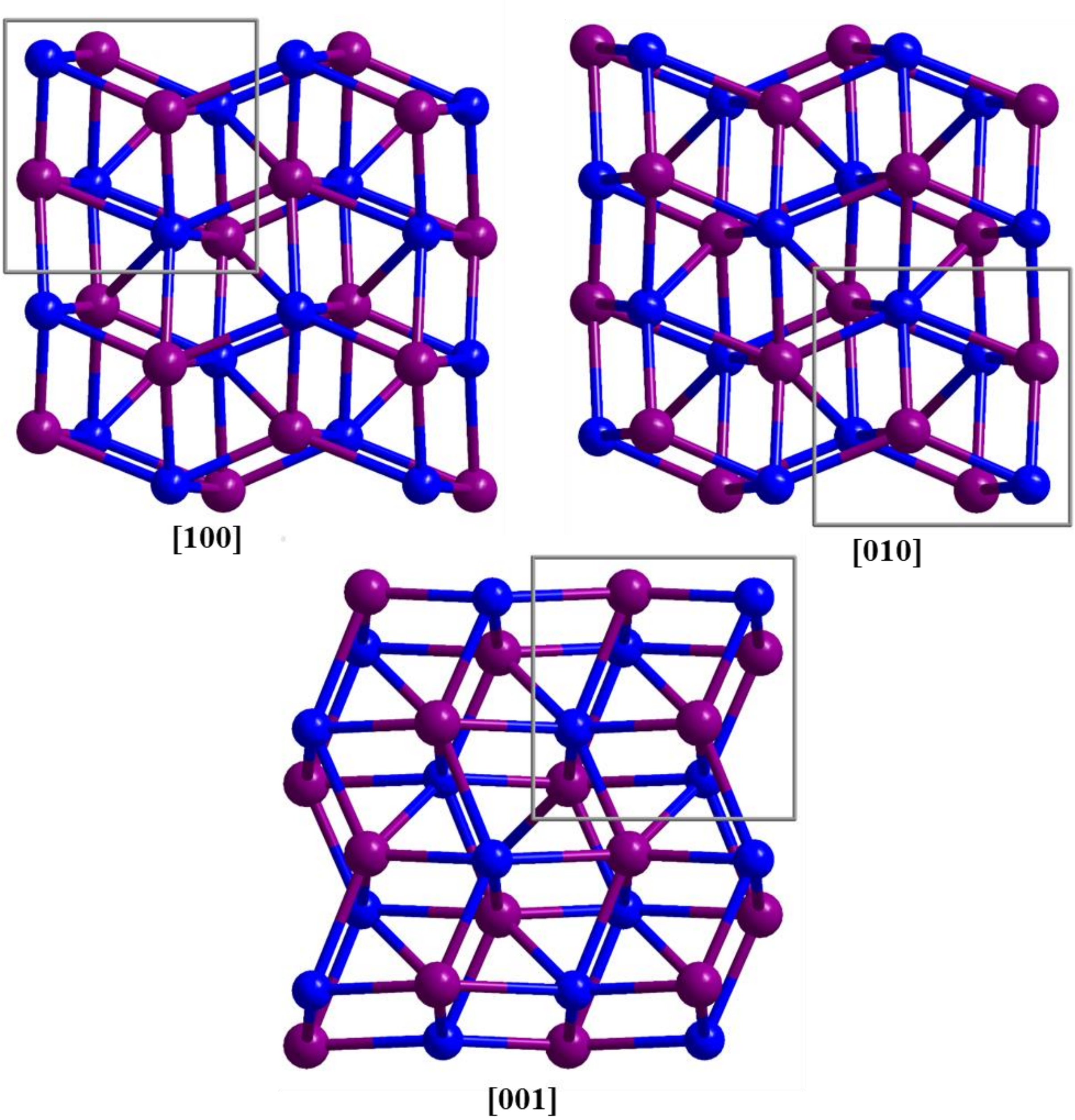

Figure S1. The crystal structure of FeSi (purple: iron; blue: Si) on crystallographic [100], [010] and [001] direction. The FeSi crystallizes in the cubic system with the space group $P2_13$ (No. 198) with the unit cell parameters a = 4.48798(4) Å, Z = 4, and V = 90.4 Å^3. The FeSi belongs to B20 structures with eight atoms per unit cell (see also Figure 1 in the main text).

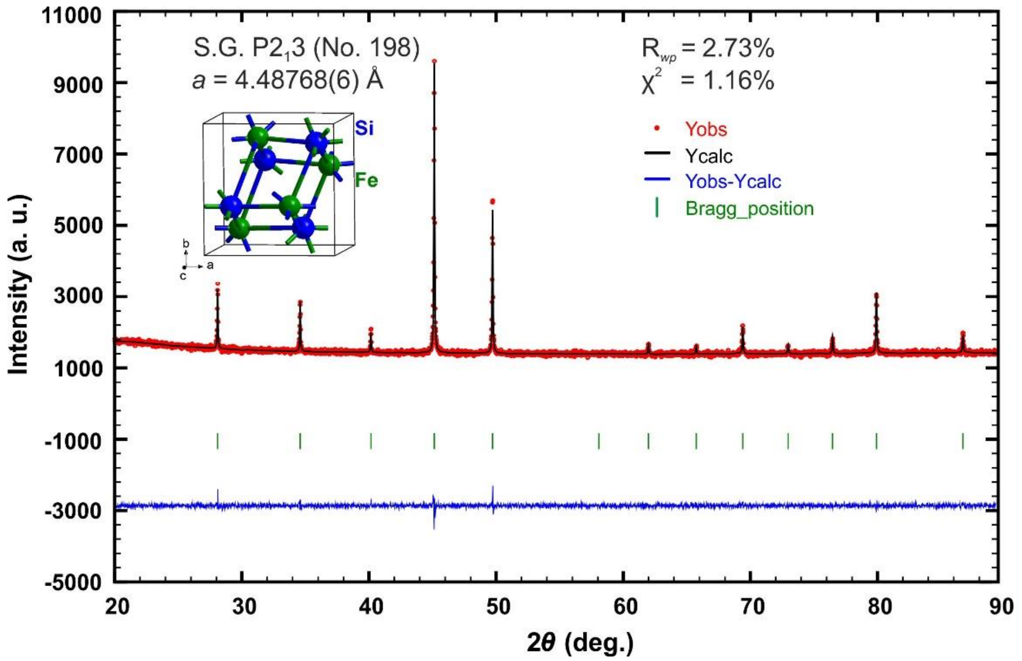

Figure S2. Rietveld refinement results for the PXRD data of the as-synthesized FeSi which was prepared through an arc-melting technique using elemental iron and silicon. The phase-purity of the product and its elemental composition were further identified by HRTEM, EDX, ICP-AES, elemental mapping, XPS, Raman, and XAS studies (see main text for a detailed analysis). The BET surface area of FeSi was 2.1 m^2g^{-1}.

Table S1. Determination of the iron to silicon ratio in FeSi powder, as deposited on FTO and after OER-CP (24 h). The ratios were obtained by ICP-AES and EDX analysis. Each experiment was conducted thrice independently, and the average value is presented.

	Fe:Si (Theo.)	Fe:Si (EDX)	Fe:Si (ICP-AES)
FeSi powder	1:1	1:1.04	1:1.01
FeSi deposited	1:1	1:1.01	-
FeSi OER-CP 3 h 10 mA/cm²		1:0.89	
FeSi OER-CP 24 h 10 mA/cm²	-	1:0.72	1:0.71
FeSi OER-CP 24 h 100 mA/cm²		1:0.71	1:0.70

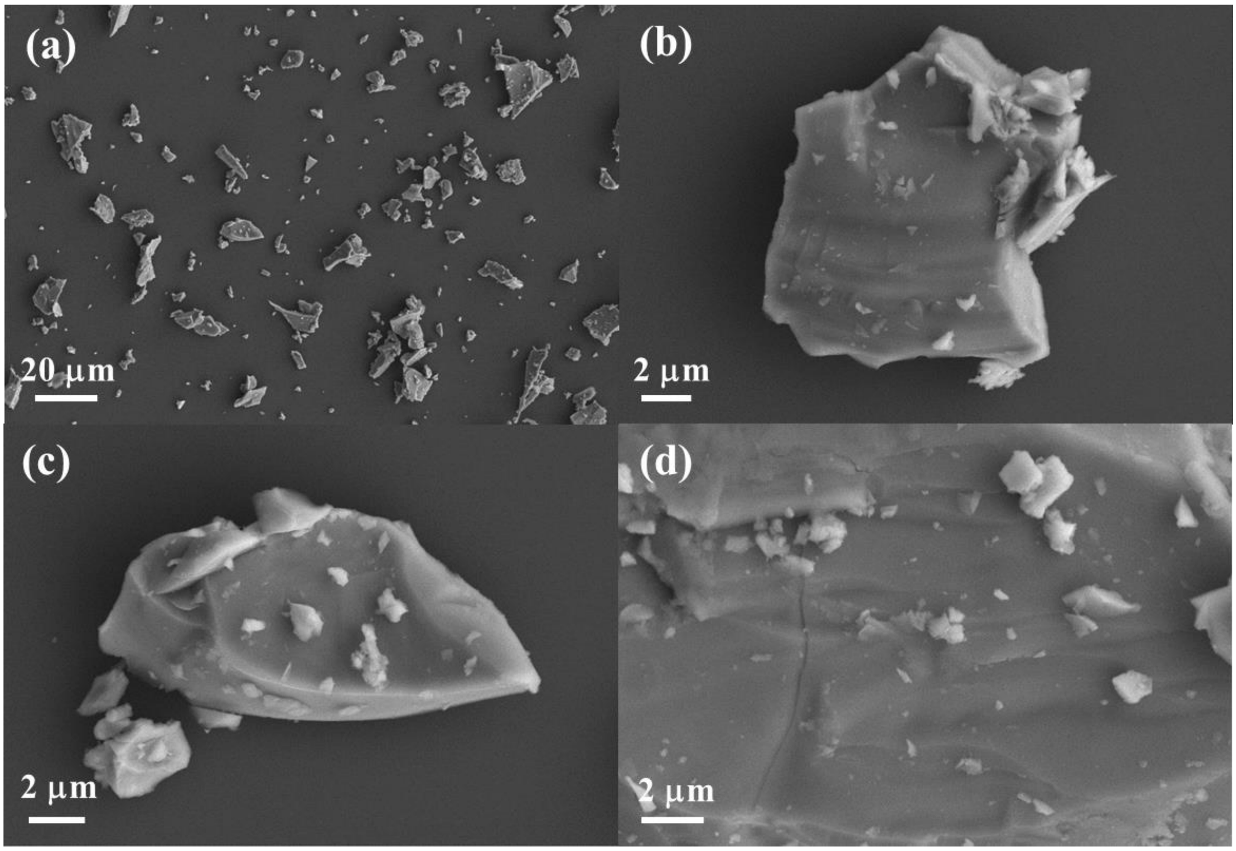

Figure S3. SEM images (a-d) of the as-synthesized FeSi powder at different magnifications. The morphology of FeSi showed irregularly shaped particles with varying sizes of 1-40 μm.

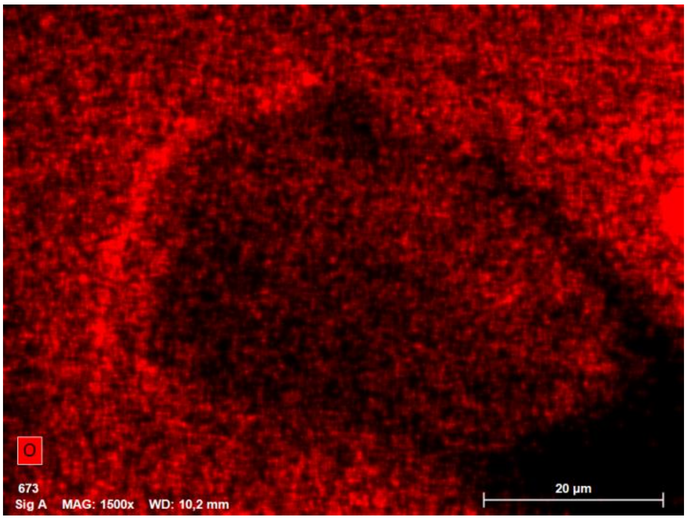

Figure S4. EDX mapping of oxygen on the FeSi crystals (for the SEM image and Fe/Si mapping refer to Figure 1 in the main text). The measured oxygen content was <1 %. The EDX spectrum is shown in Figure S4.

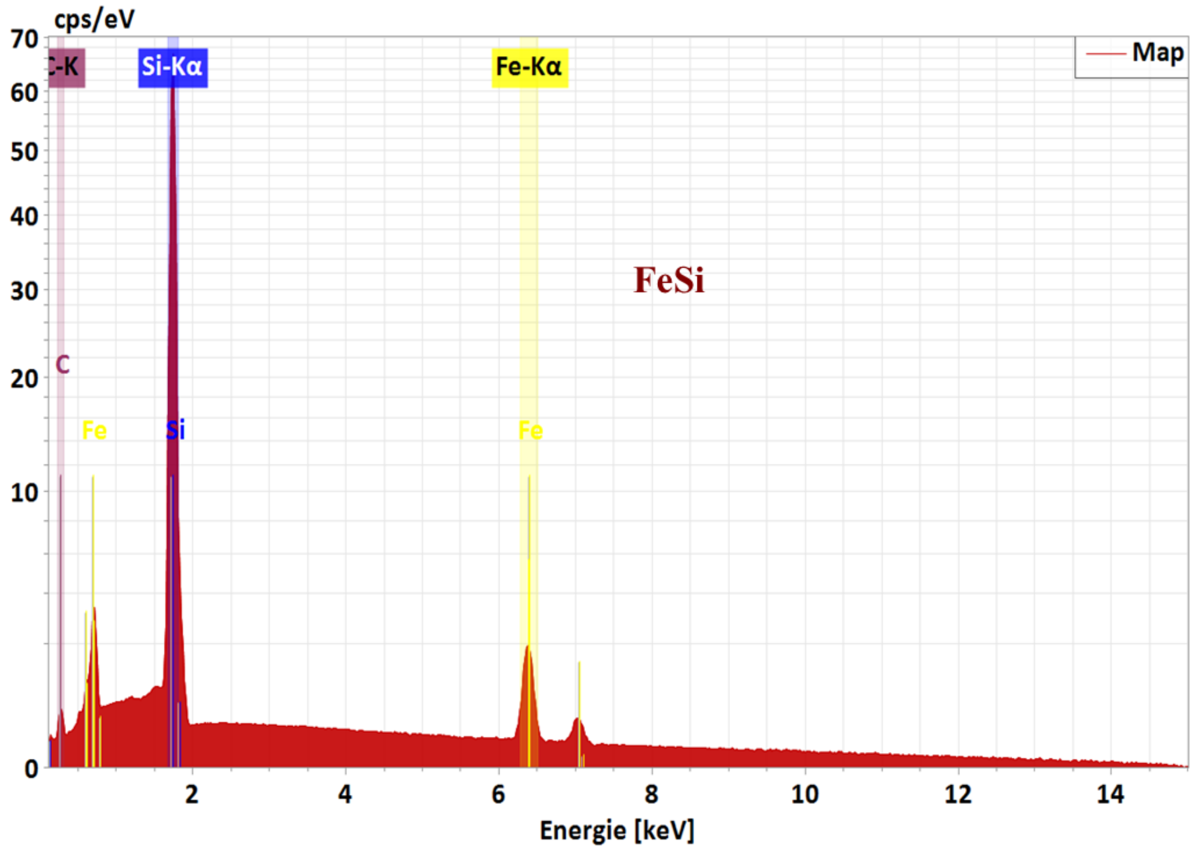

Figure S5. The EDX mapping spectrum of the as-prepared FeSi powder that confirms the presence of Fe and Si.

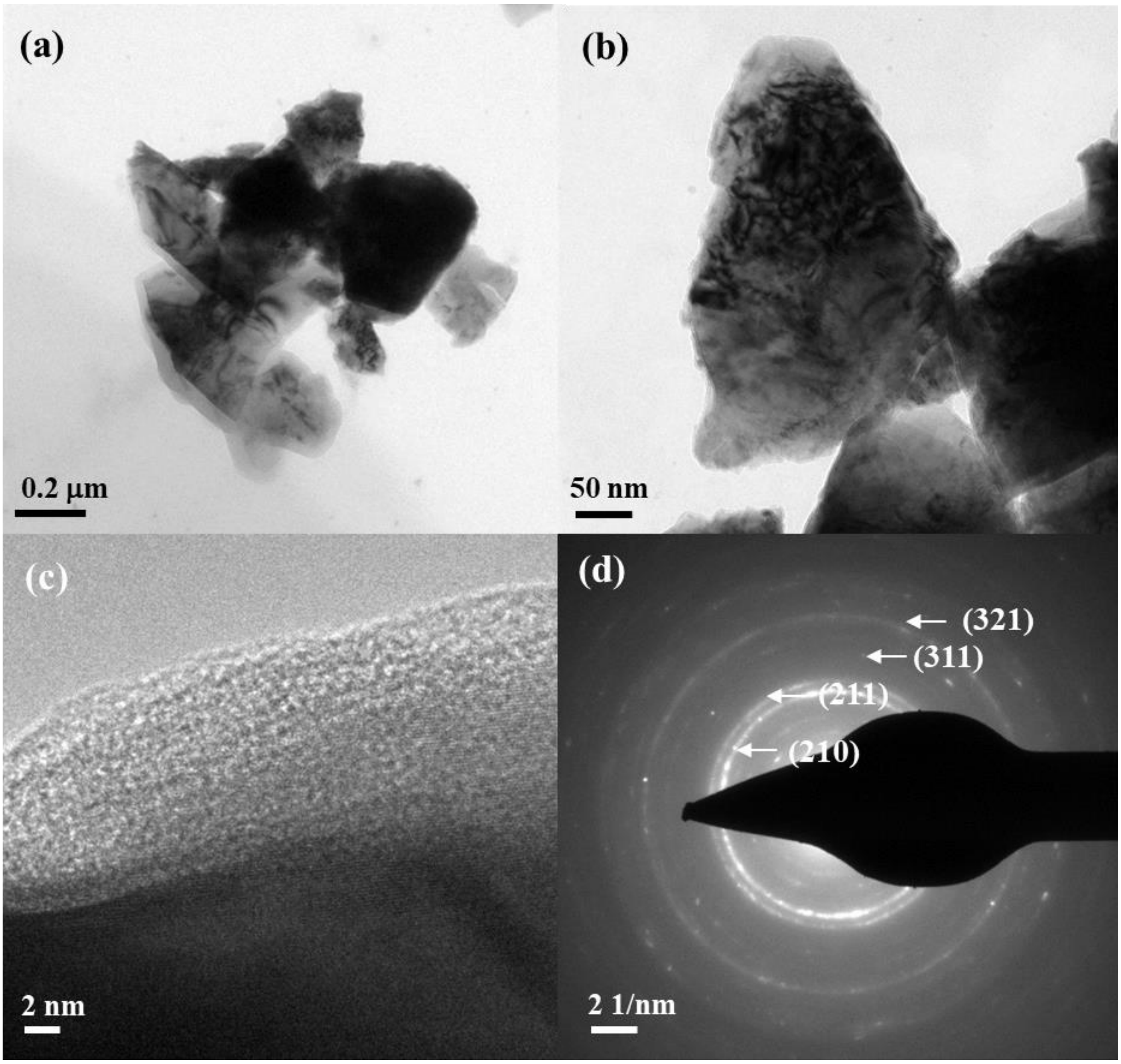

Figure S6. TEM images (a, b) and high-resolution TEM (HR-TEM) images (c) of the as-prepared FeSi reveal irregularly shaped particles with varying sizes. The selected area diffraction pattern (SAED, d) indicates that the particles were crystalline. The SAED pattern display diffraction rings corresponding to the crystallographic planes (210), (211) (311), and (222) at d = 0.20, 0.18, 0.13, and 0.12 nm, which is also consistent with the PXRD (JCPDS 38-1397) pattern confirming the phase purity of the product.

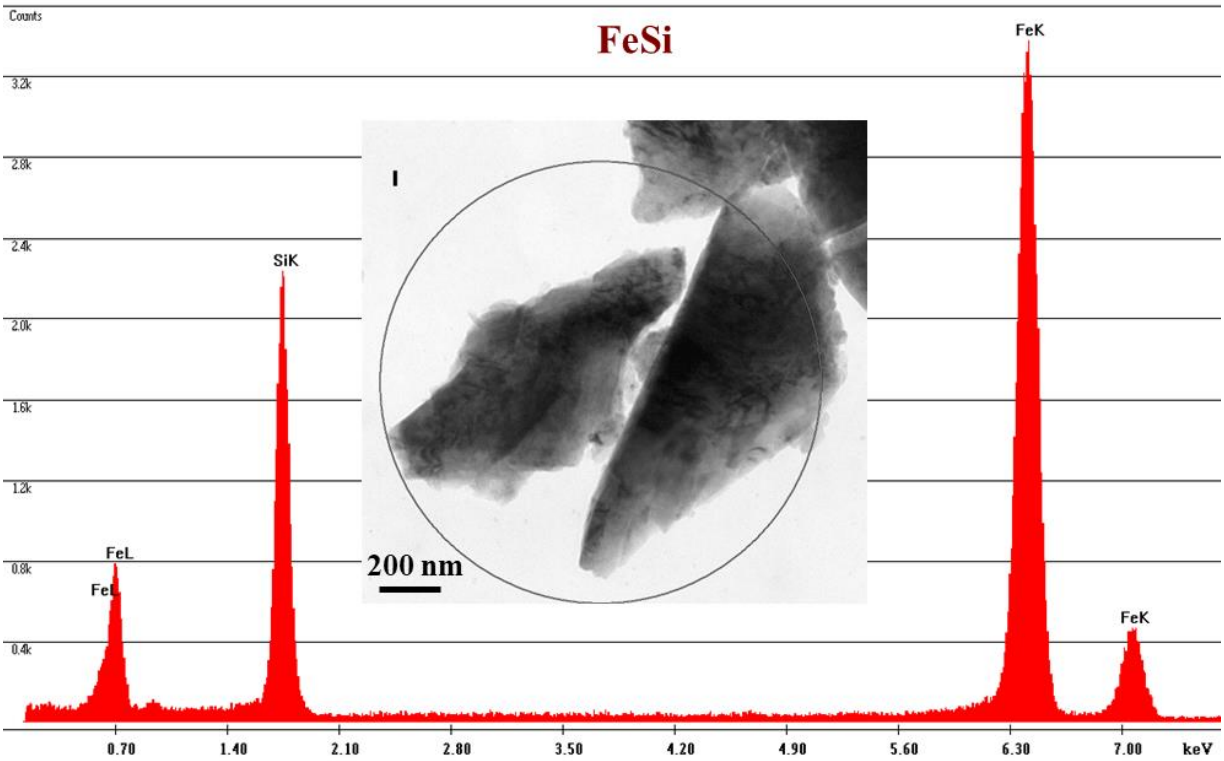

Figure S7. EDX analysis of the as-synthesized FeSi powder evidencing the presence of Fe and Si. The inset shows the region used for the EDX. For the measurement, a carbon film on 300 mesh Cu-grid was used.

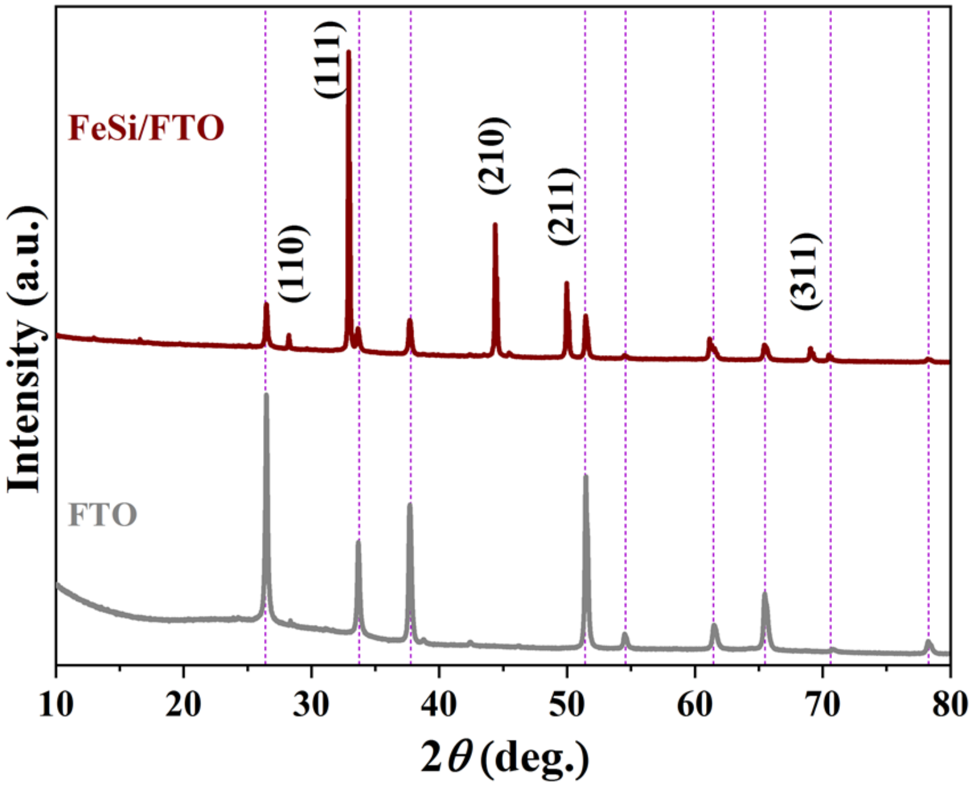

Figure S8. PXRD pattern of the electrophoretically deposited intermetallic FeSi film on FTO (FeSi/FTO) confirming the chemical stability of the structure (JCPDS 38-1397). Besides, the diffraction pattern of FTO is also provided and the peaks responsible for FTO are highlighted with a pink dotted line.

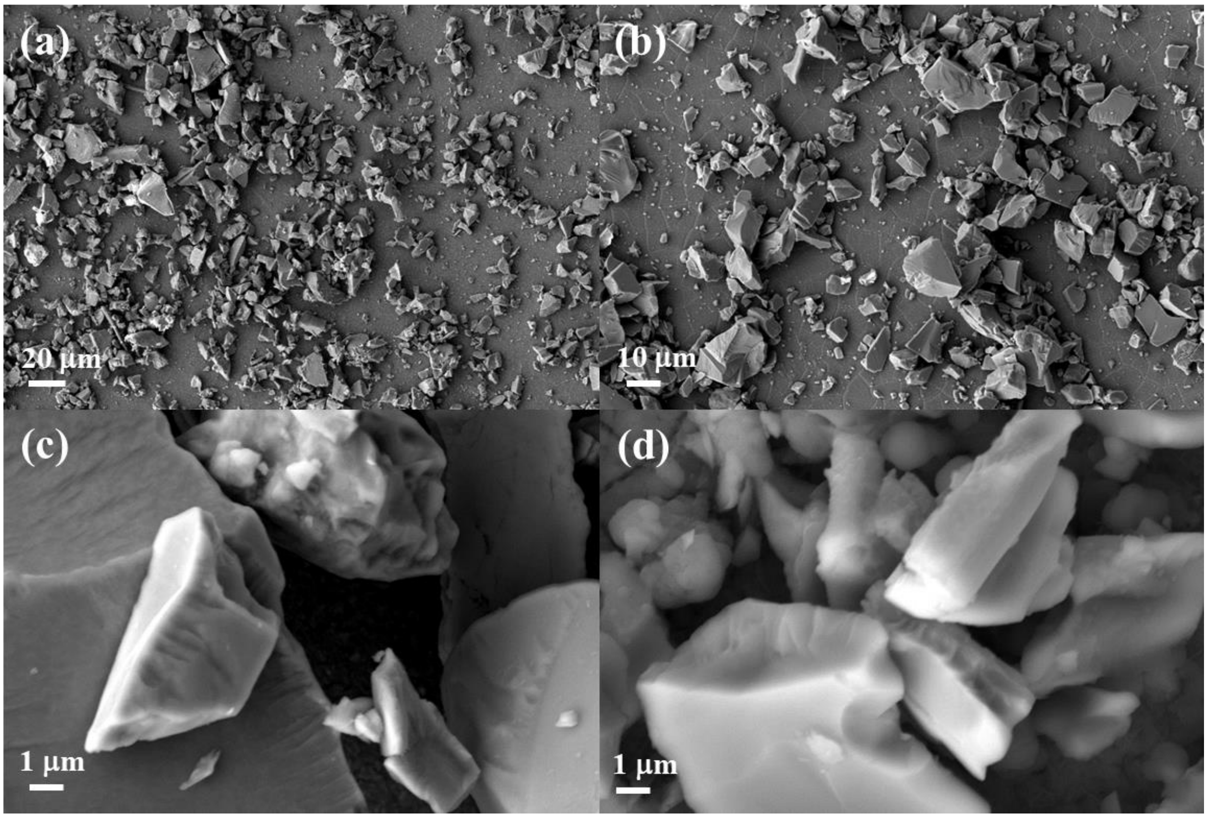

Figure S9. SEM images (a-d) of the electrophoretically deposited FeSi film on FTO substrate (FeSi/FTO) at different magnifications showing particles that are 1-40 μm large.

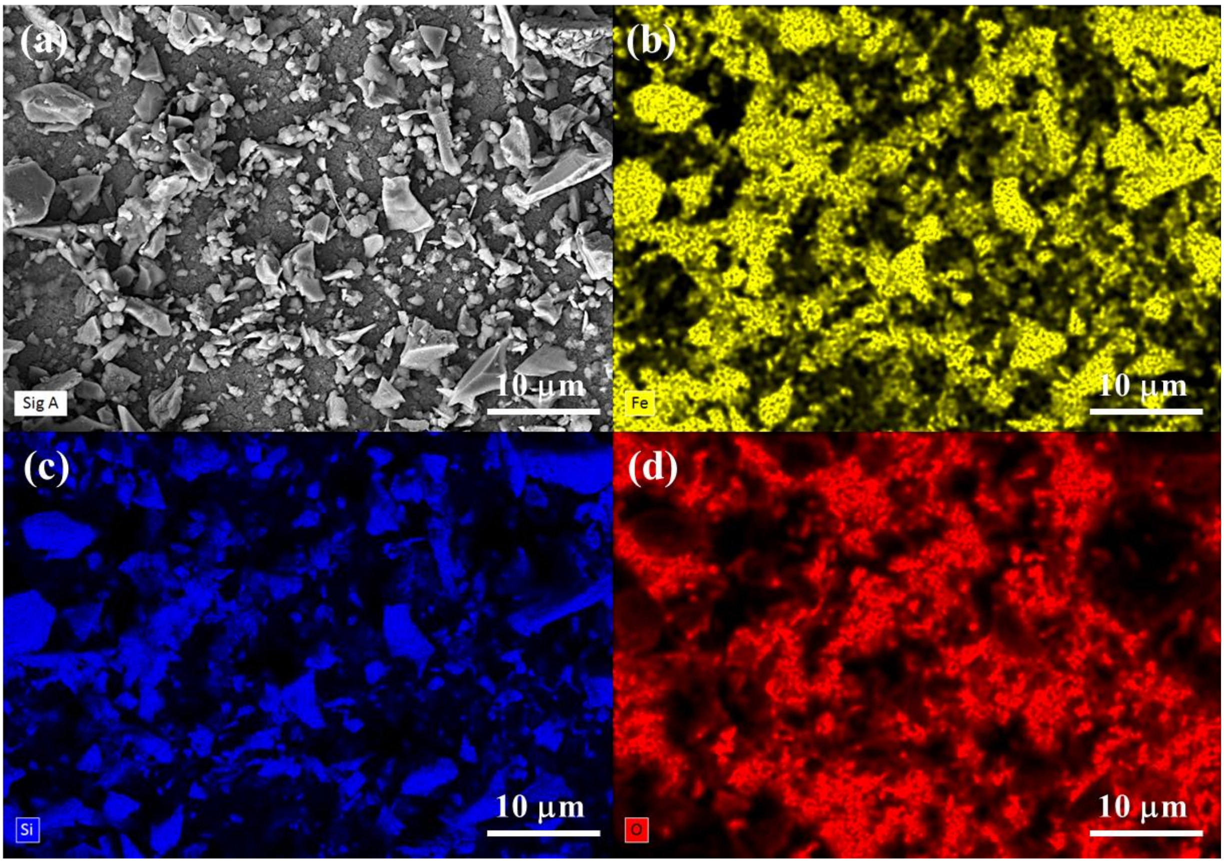

Figure S10. SEM image (a) and the EDX mapping (b-d) performed on the electrophoretically deposited FeSi/FTO film showing a homogenous distribution of Fe (yellow) and Si (red) in the structure. The EDX spectrum is shown in Figure S11.

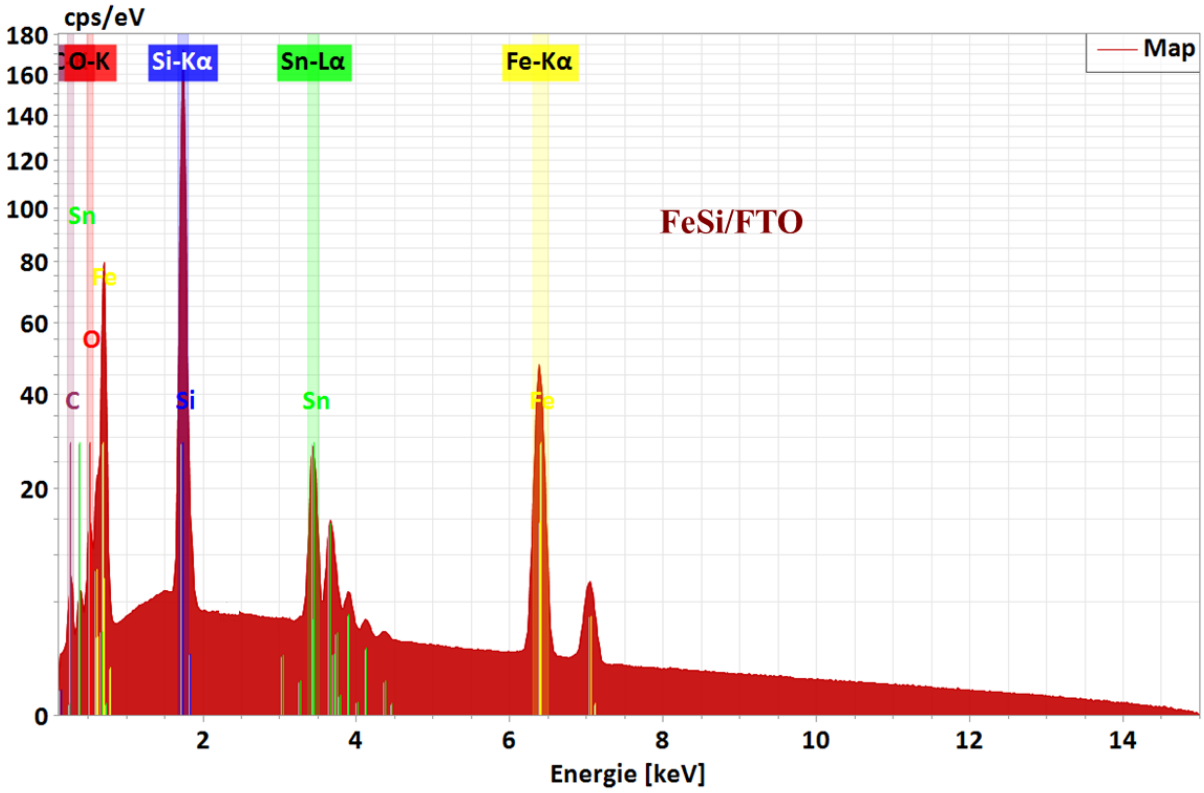

Figure S11. The EDX mapping spectrum of as-deposited FeSi/FTO film. Sn and O signals arise from the FTO glass substrate electrode.

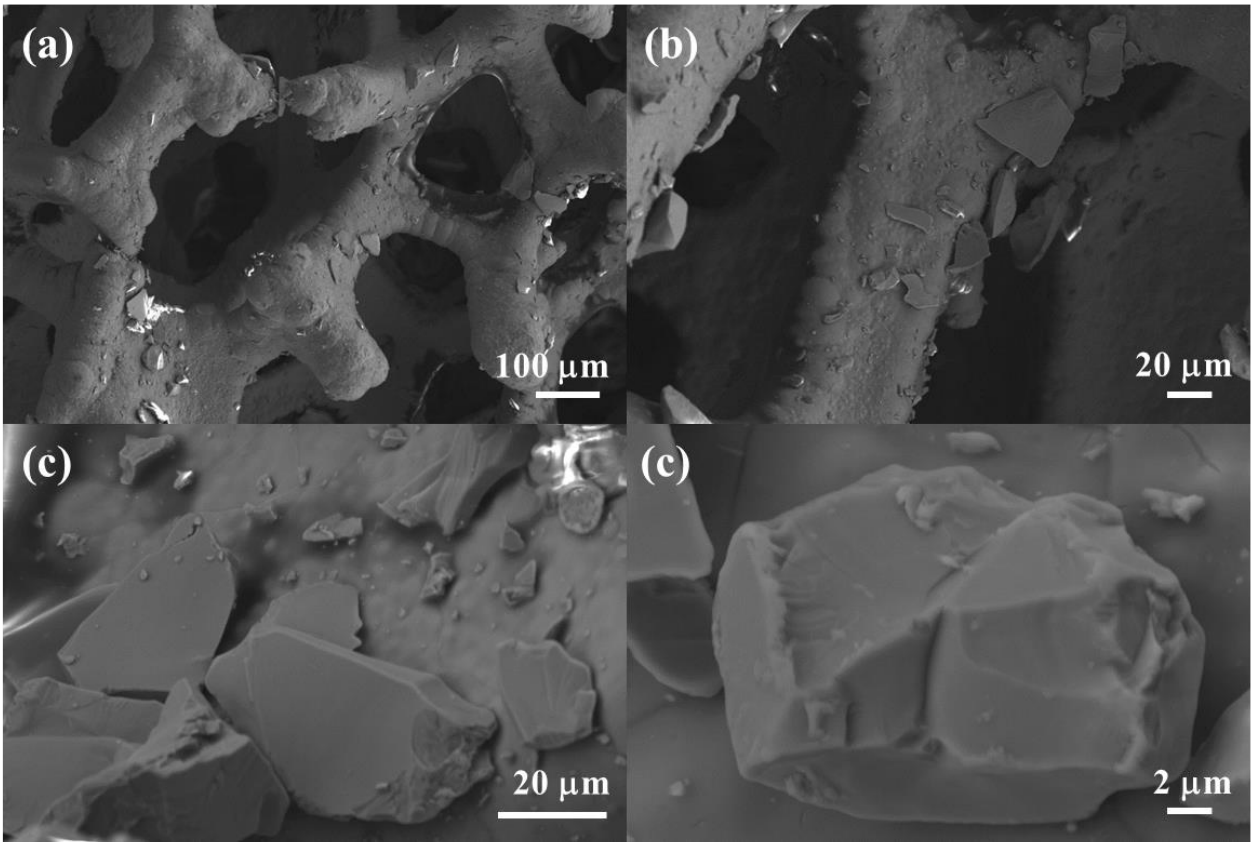

Figure S12. SEM images (a-d) of FeSi films on NF (FeSi/NF) at different magnifications.

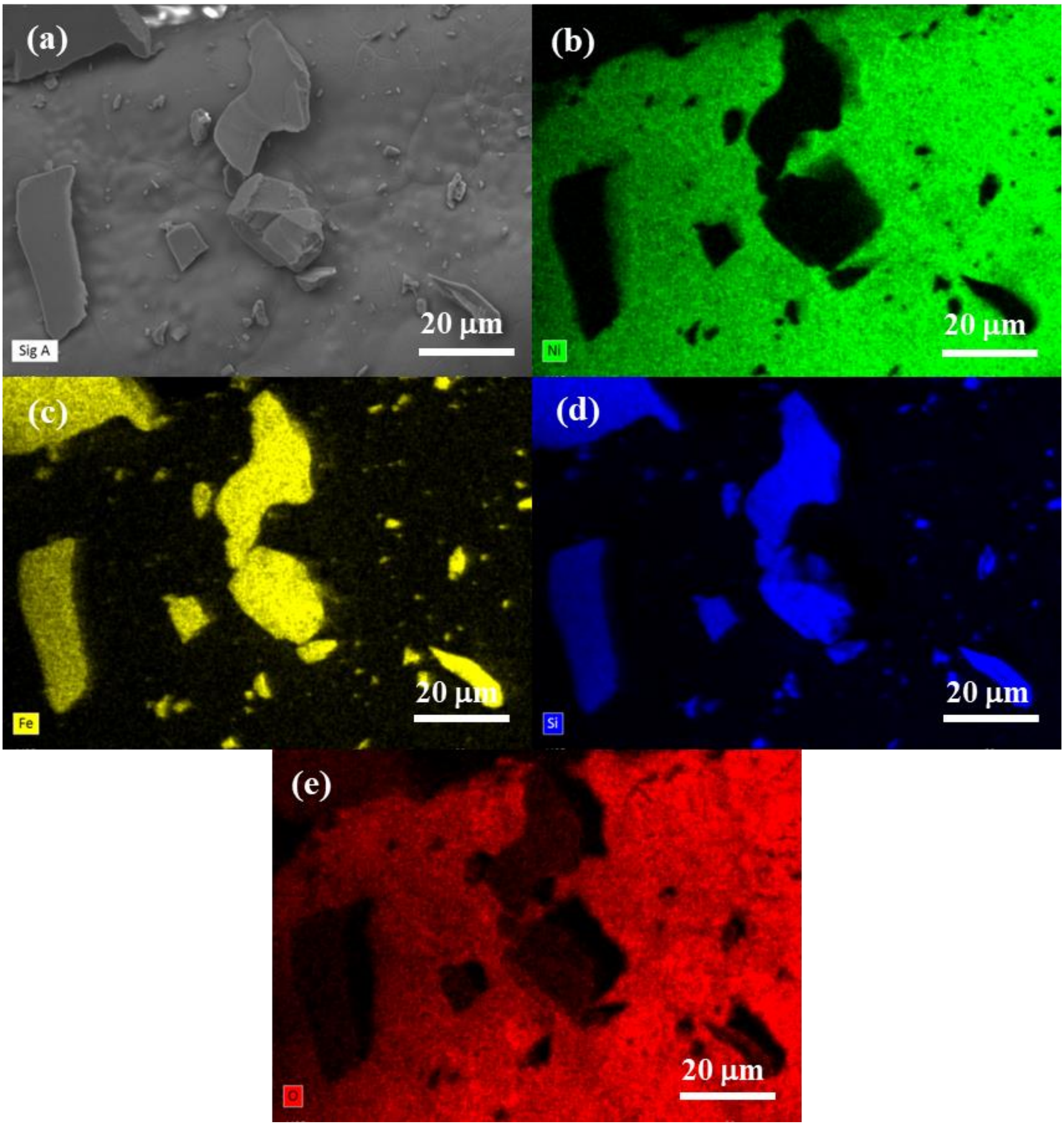

Figure S13. SEM image (a) and the respective EDX mapping of a FeSi/NF film (b-e). Nickel (b, green) is only present on the NF substrate. The mapping images exhibit homogenously distributed iron (c, yellow) and silicon (d, blue) within the FeSi particles. Notably, the results are also consistent with FeSi/FTO verifying the chemical stability of FeSi upon film deposition (oxygen content in FeSi particles <1%). The EDX spectrum is presented in Figure S14.

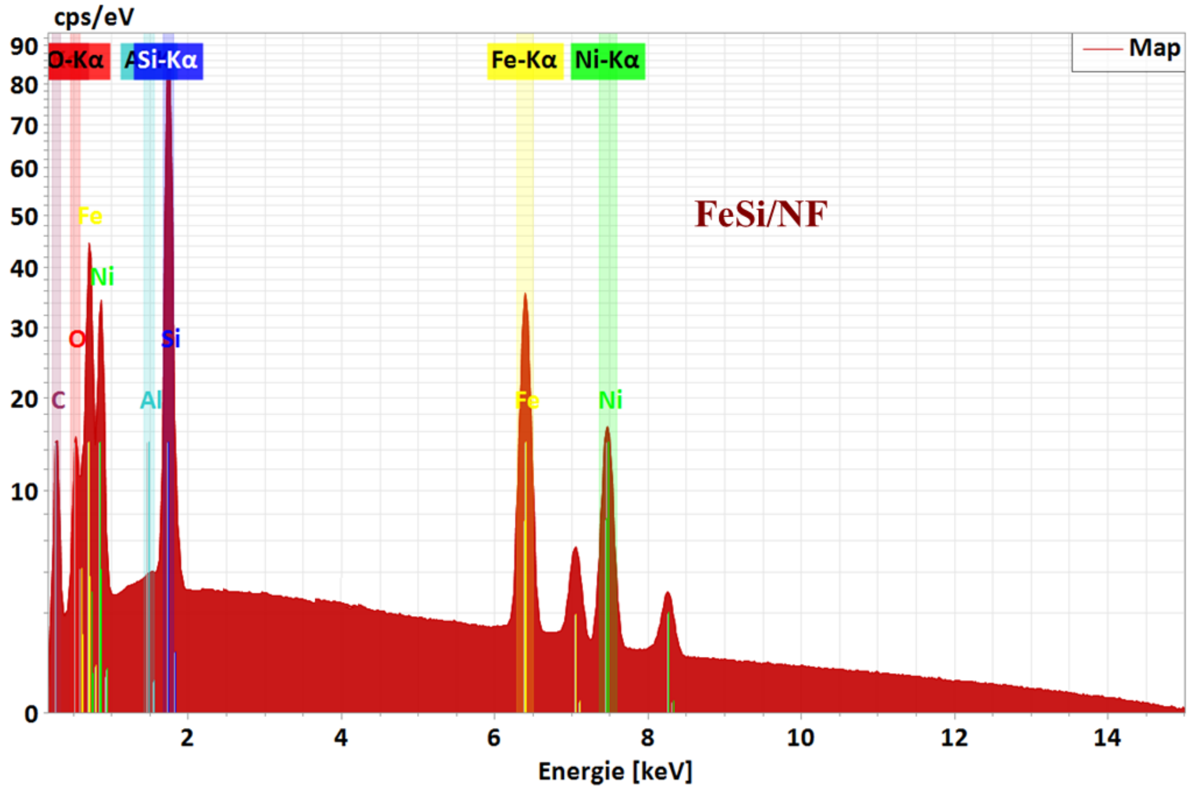

Figure S14. The EDX mapping spectrum of FeSi/NF. The peaks of Ni are from the nickel substrate while the Al peaks arise from the Al sample holder

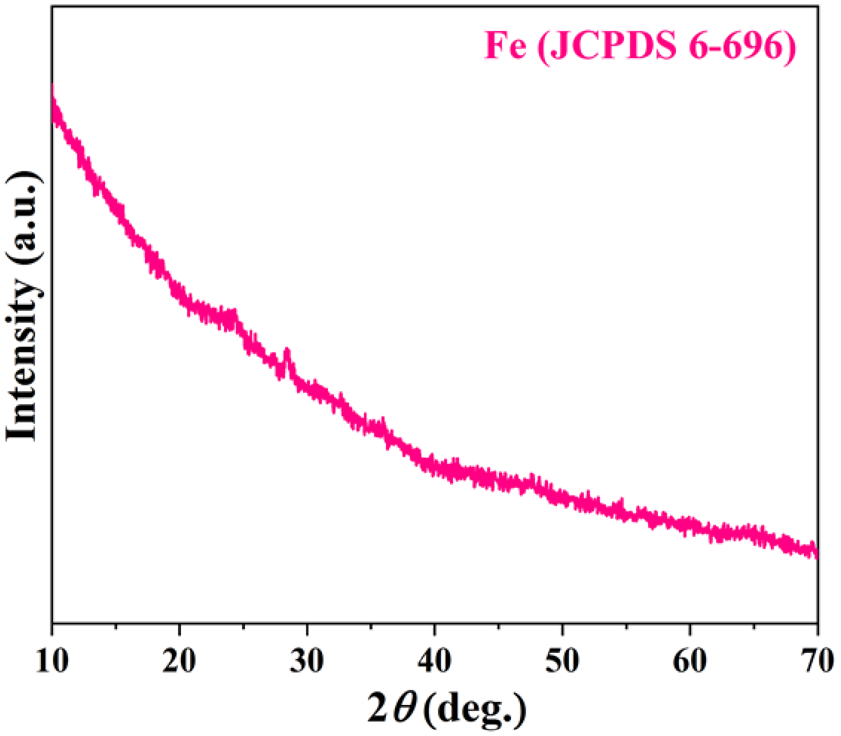

Figure S15. PXRD pattern of the as-synthesized iron nanoparticles that were prepared by a solution-based method (see synthesis of the catalysts).[5] The broad pattern indicates that the crystallites are small. The BET area of the Fe was found to be 27.4 m^2g^{-1}.

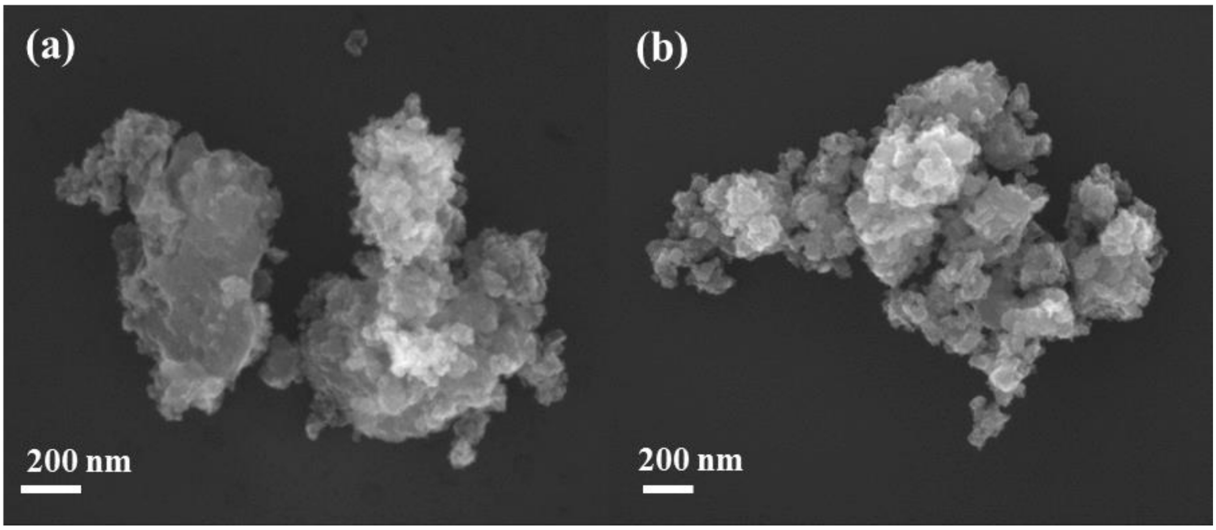

Figure S16. SEM images (a-b) of the as-prepared Fe nanoparticles.

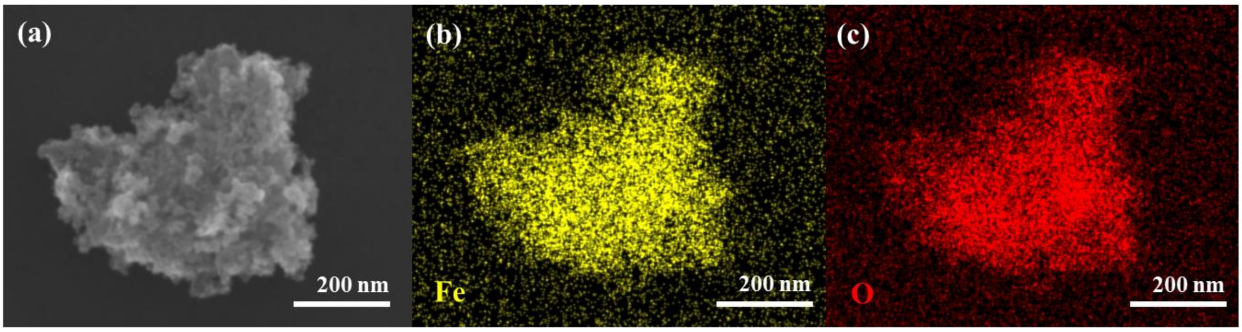

Figure S17. SEM image (a) and the EDX mapping (b-c) of the as-prepared Fe nanoparticles. The Fe is represented in color yellow. About 10% of oxygen (red) was also detected which could be attributed to the surface passivation of metallic Fe. The EDX spectrum is presented in Figure S19.

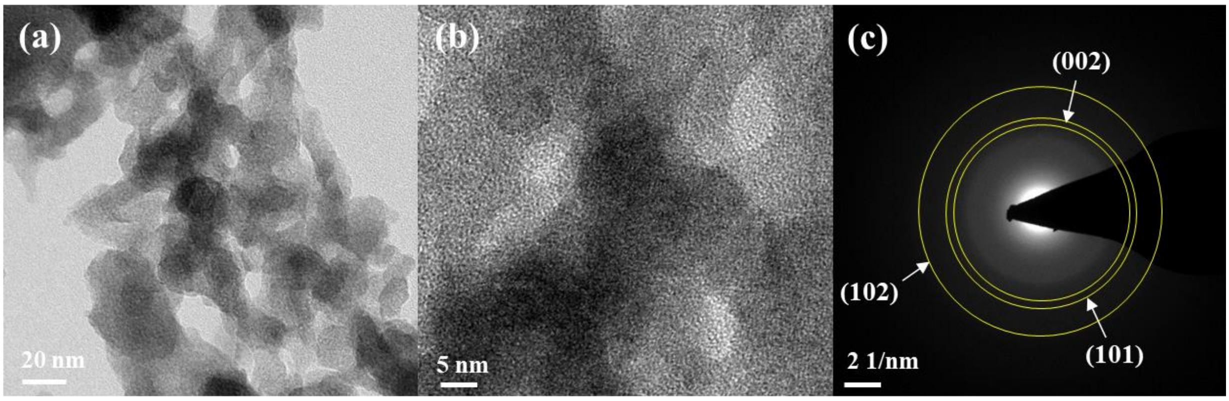

Figure S18. TEM (a), HRTEM (b), and SAED pattern (c) of the as-synthesized iron nanoparticles. Selected area electron diffraction (SAED) of the Fe displays weak diffraction rings corresponding to (002), (101), and (102) crystal planes. These planes are consistent with the formation of Fe^0 (JCPDS 34-529).[15]

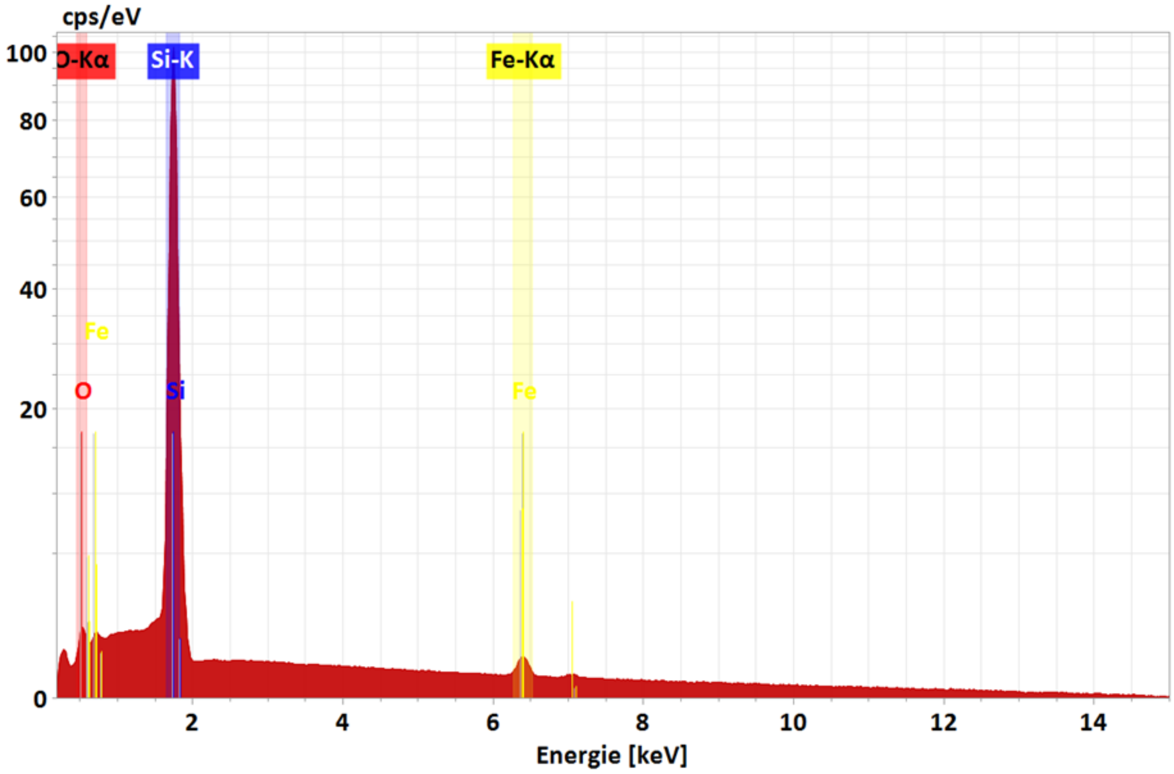

Figure S19. The EDX mapping spectrum of the as-prepared Fe nanoparticles. Si peaks arise from the Si wafer substrate.

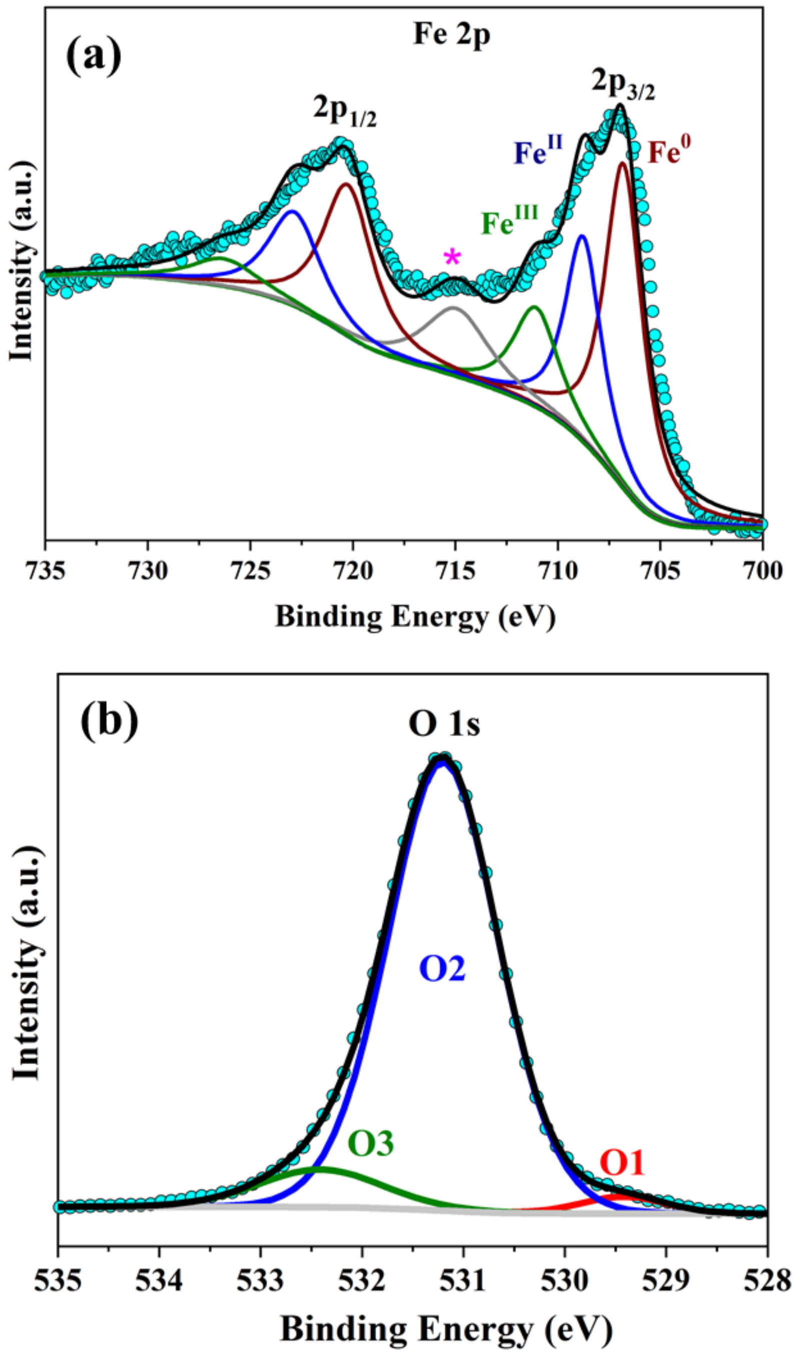

Figure S20. (a) Fe 2p XPS spectrum of the as-prepared Fe^{0} nanoparticles. The deconvoluted $2p_{3/2}$ and $2p_{1/2}$ peaks exhibit sharp peaks at 706.8 and 720.3 eV that could be ascribed to Fe^{0} while the peaks at higher binding energies are representative of the oxidized species of iron (Fe^{II} and Fe^{III}) that occur via surface passivation in air.[11,13,15] The O 1s spectrum of Fe nanoparticles showed mainly surface hydroxylation (O2) along with slight oxide (O1) species and adsorbed water (O3).[16]

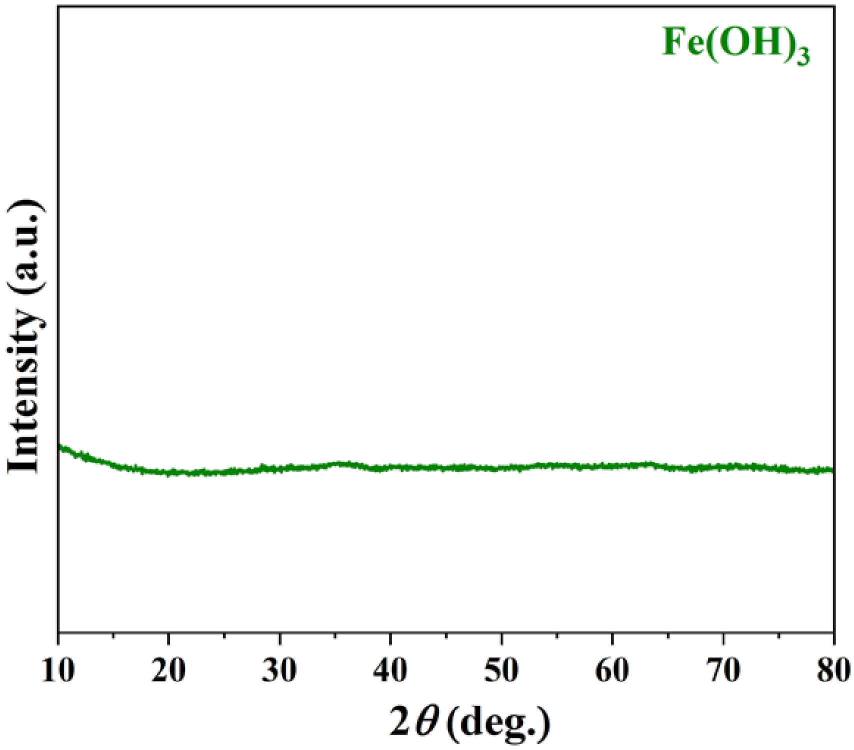

Figure S21. PXRD pattern of the as-synthesized $Fe(OH)_3$ was synthesized by the previously reported procedure. The BET surface area of the $Fe(OH)_3$ was found to be 185.2 m^2g^{-1}.

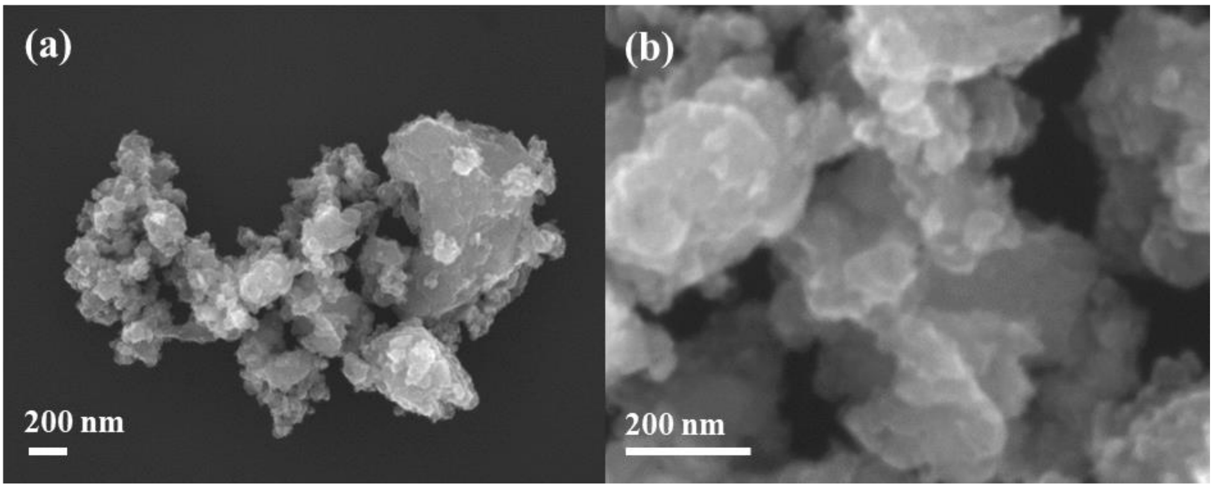

Figure S22. SEM images (a-b) of the as-prepared $Fe(OH)_3$ nanoparticles.

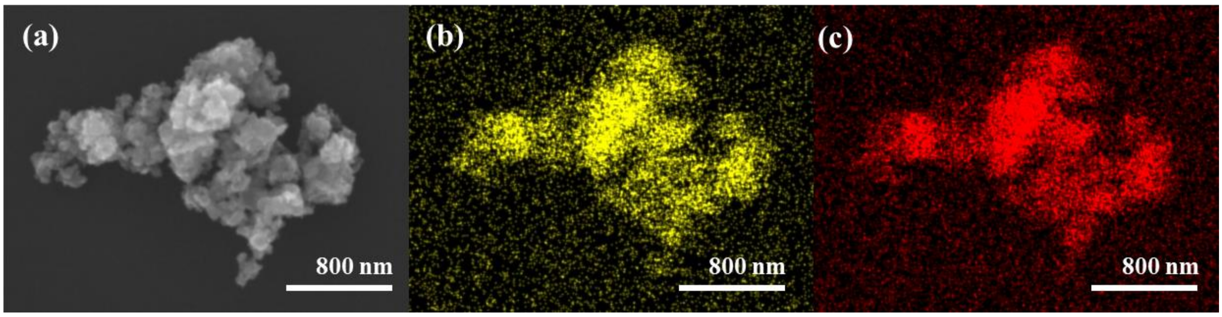

Figure S23. SEM image (a) and the EDX mapping (b-c) of the as-prepared $Fe(OH)_3$ nanoparticles. The mapping images displayed a homogenous distribution of Fe (yellow) and O (red). The EDX spectrum is presented in Figure S24.

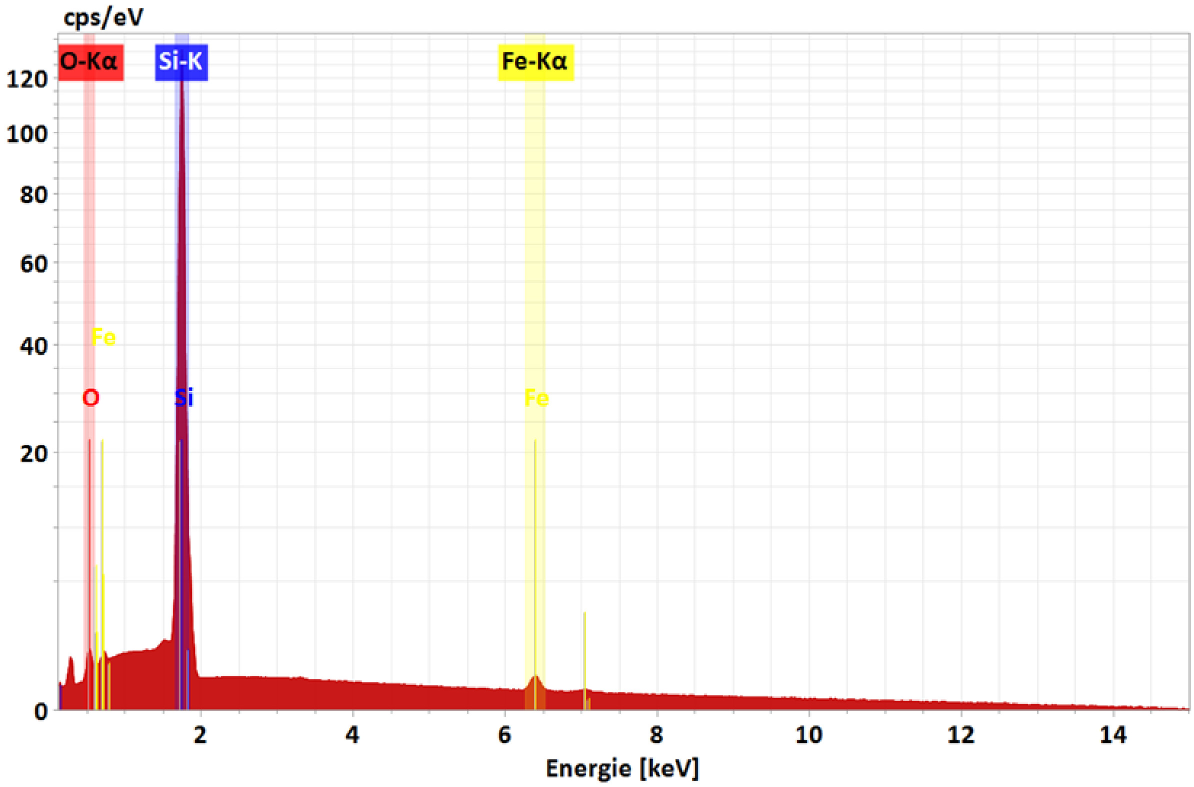

Figure S24. The EDX mapping spectrum of the as-prepared $Fe(OH)_3$ nanoparticles. Si peaks arise from the Si wafer substrate.

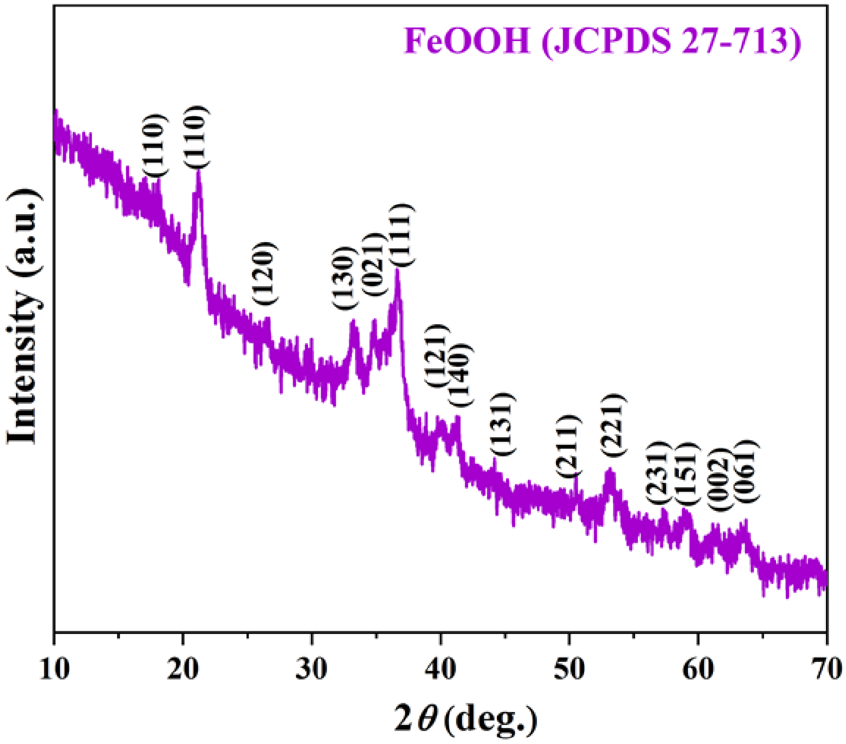

Figure S25. PXRD pattern and Miller indices of as-synthesized FeOOH (JCPDS 27-713). The BET surface area of the FeOOH was found to be 83.1 m^2g^{-1}.

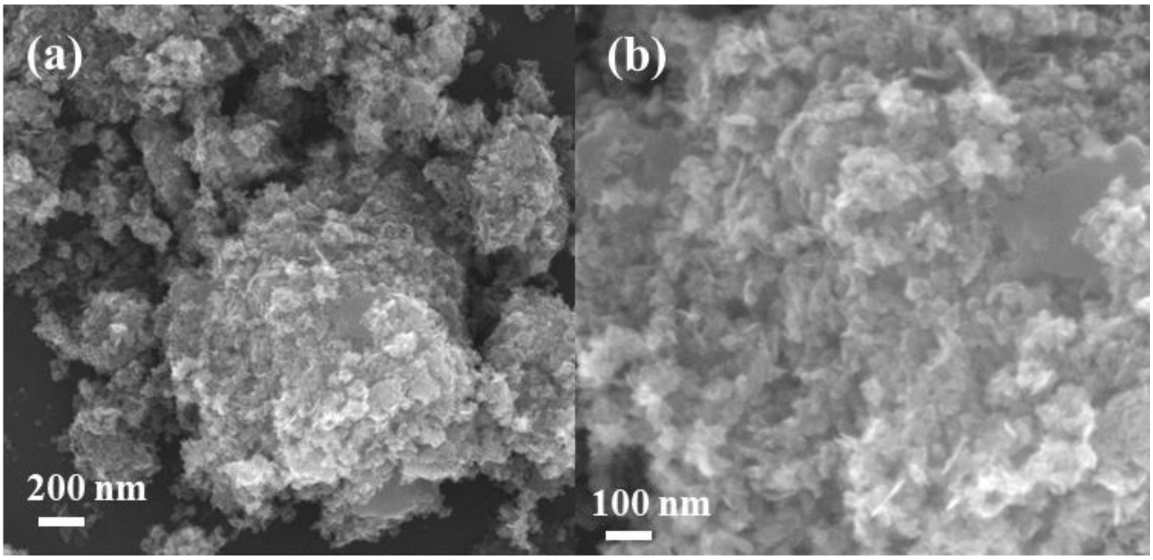

Figure S26. SEM images (a-b) of the as-prepared FeOOH nanoparticles.

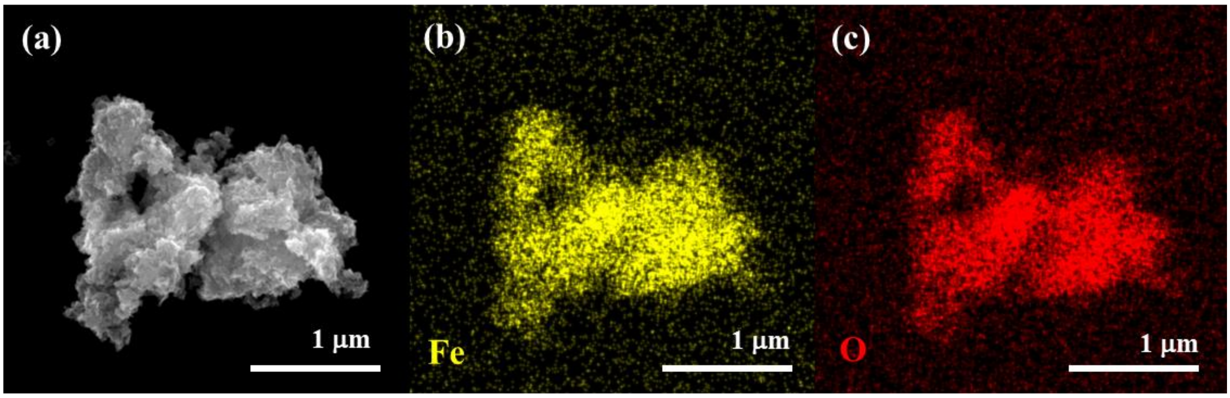

Figure S27. SEM image (a) and the EDX mapping (b-c) of the as-prepared FeOOH nanoparticles. The mapping images displayed a homogenous distribution of Fe (yellow) and O (red). The EDX spectrum is presented in Figure S28.

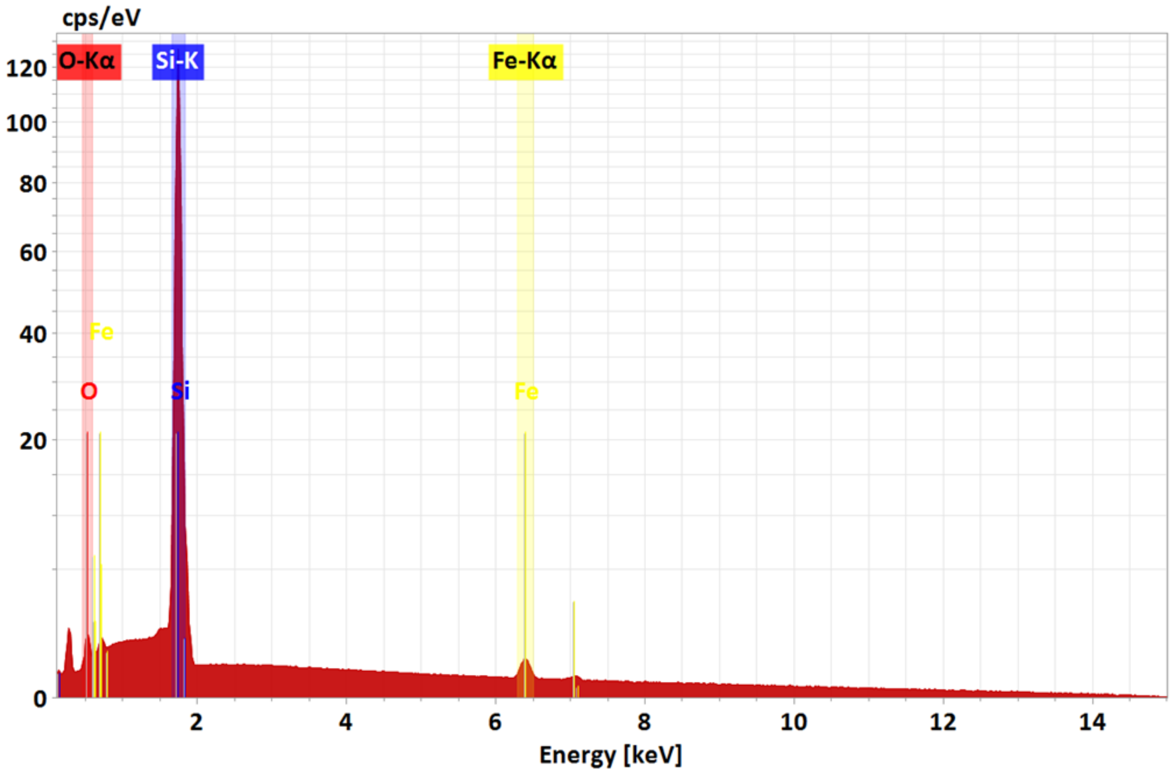

Figure S28. The EDX mapping spectrum of the as-prepared FeOOH nanoparticles. Si peaks arise from the Si wafer substrate.

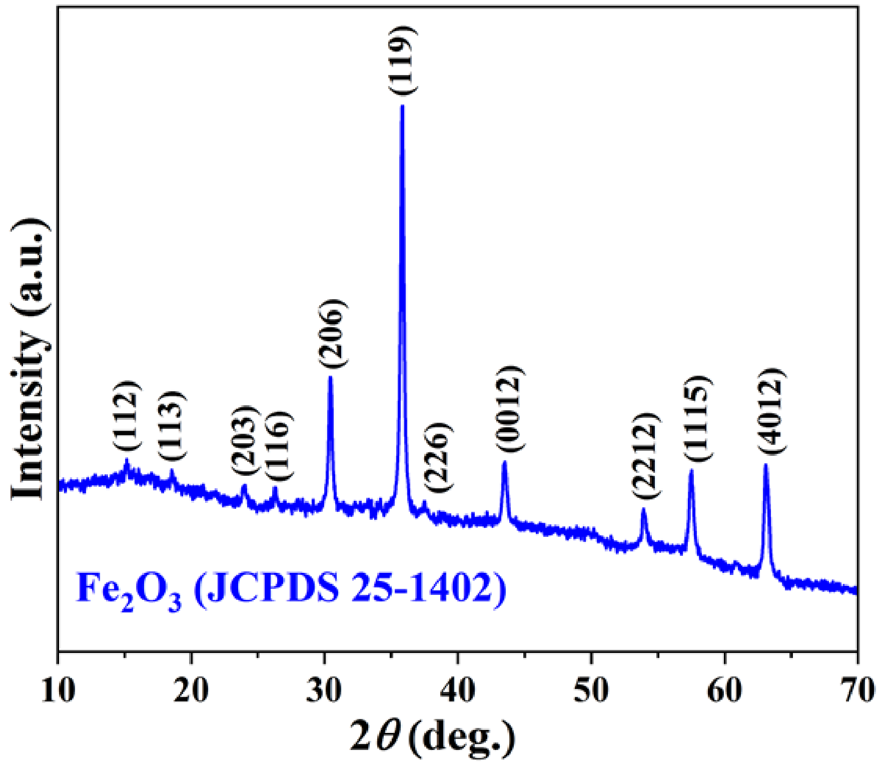

Figure S29. PXRD pattern and Miller indices of the as-synthesized Fe_2O_3 (JCPDS 25-1042). The BET surface area of the Fe_2O_3 was found to be 30.1 m^2g^{-1}.

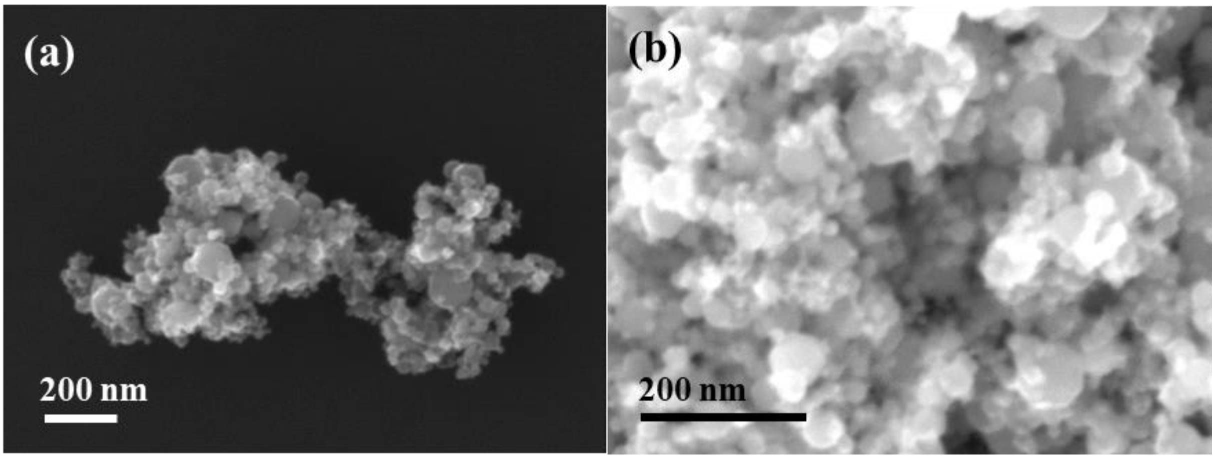

Figure S30. SEM images (a-b) of the as-prepared Fe_2O_3 nanoparticles.

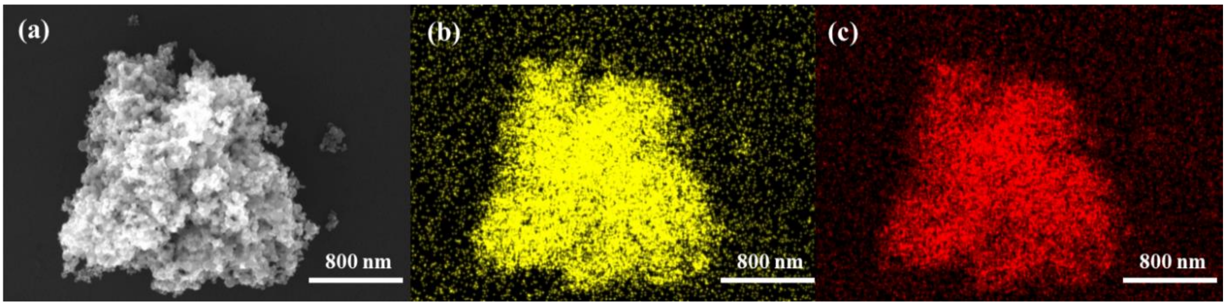

Figure S31. SEM image (a) and the EDX mapping (b-c) of the as-prepared Fe_2O_3 nanoparticles. The mapping images displayed a homogenous distribution of Fe (yellow) and O (red). The EDX spectrum is presented in Figure S32.

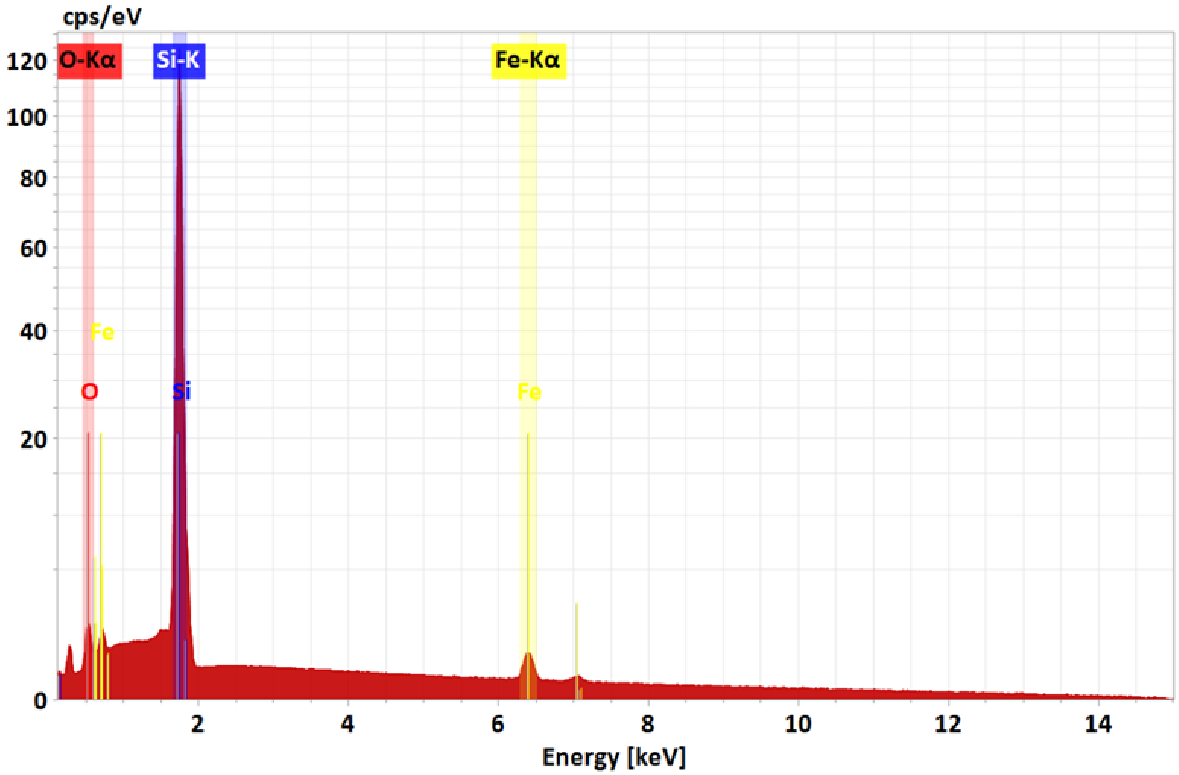

Figure S32. The EDX mapping spectrum of the as-prepared Fe_2O_3 nanoparticles. Si peaks arise from the Si wafer substrate.

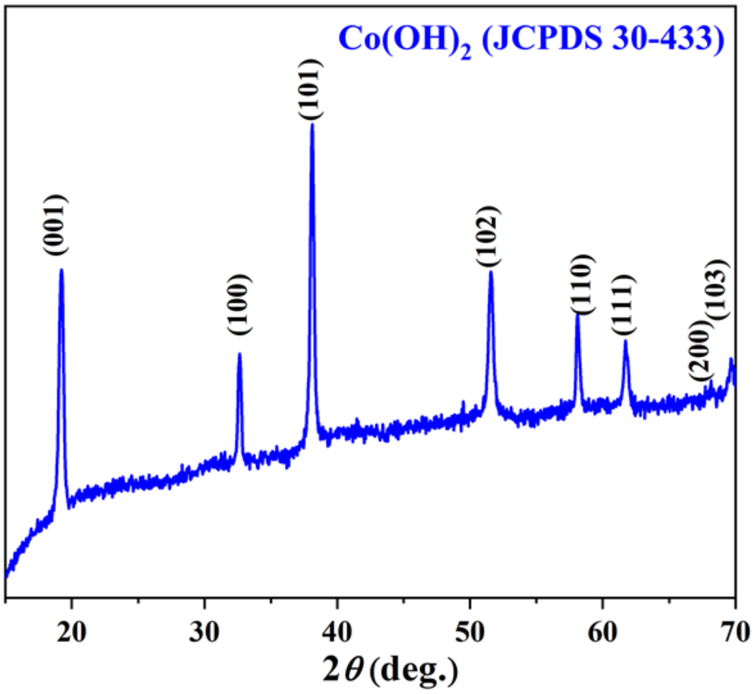

Figure S33. PXRD pattern and the Miller indices of the as-prepared $Co(OH)_2$ (JCPDS 30-433).

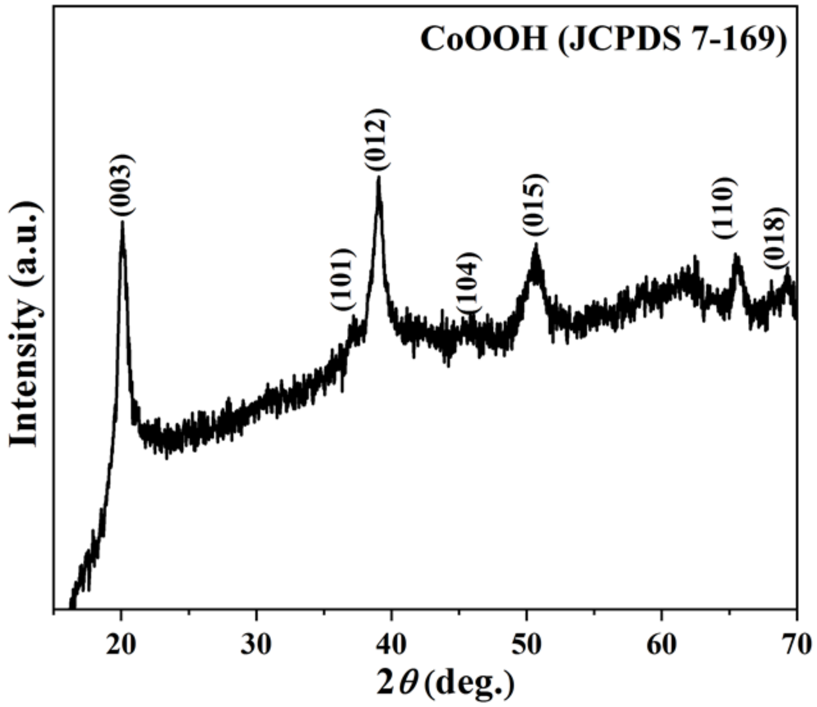

Figure S34. PXRD pattern and the Miller indices of the as-prepared CoOOH (JCPDS 7-169).

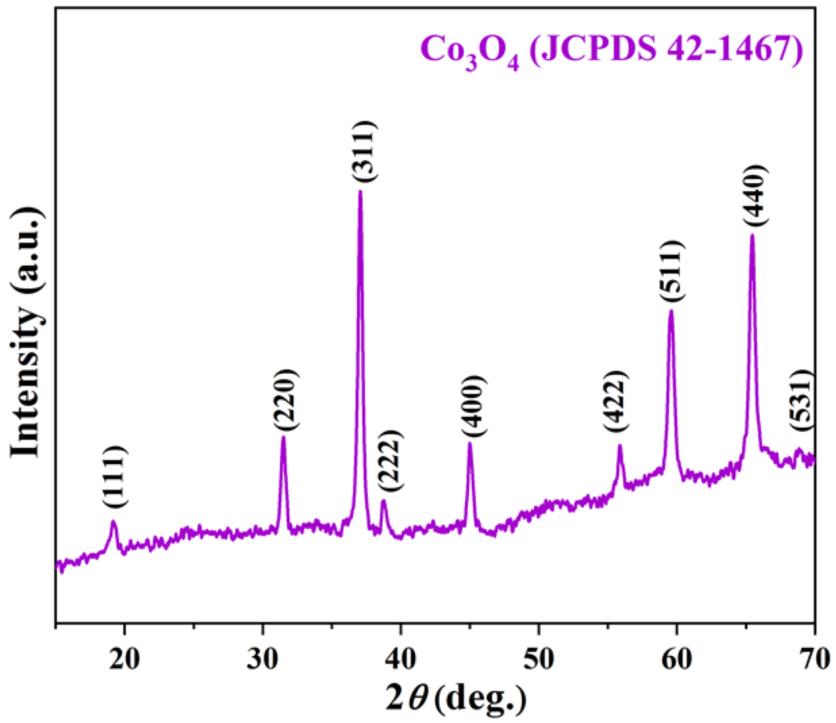

Figure S35. PXRD pattern and the Miller indices of the as-prepared Co_3O_4 (JCPDS 42-1467).

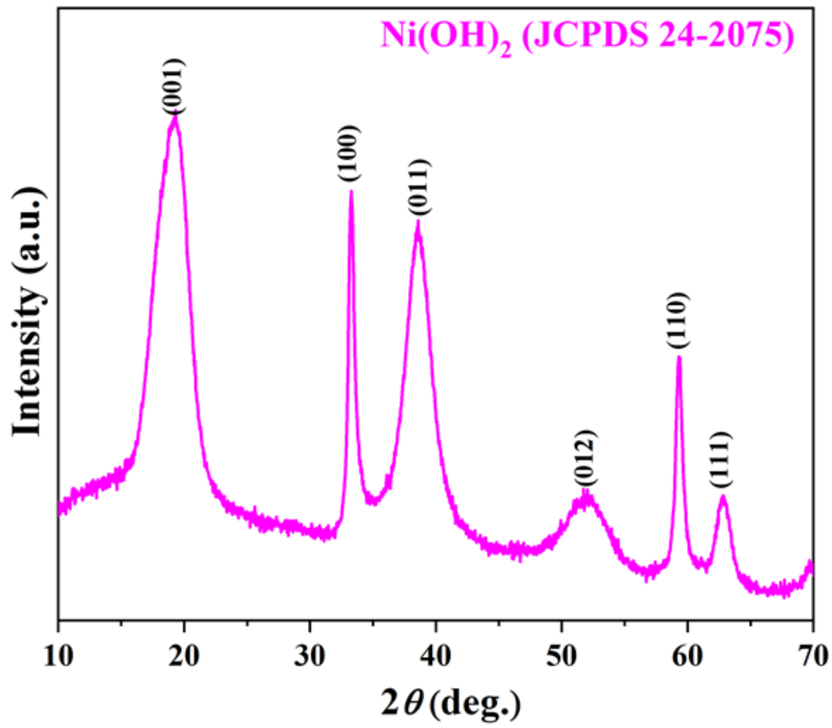

Figure S36. PXRD pattern and the Miller indices of the as-prepared $Ni(OH)_2$ (JCPDS 24-2075).

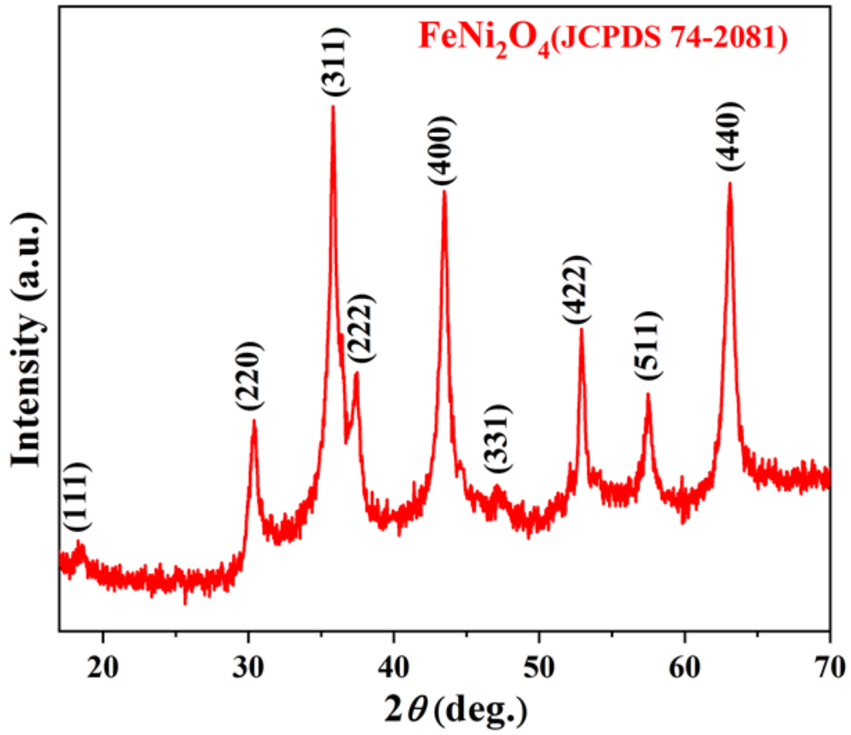

Figure S37. PXRD pattern and the Miller indices of the as-prepared $FeNi_2O_4$ (JCPDS 74-2081).

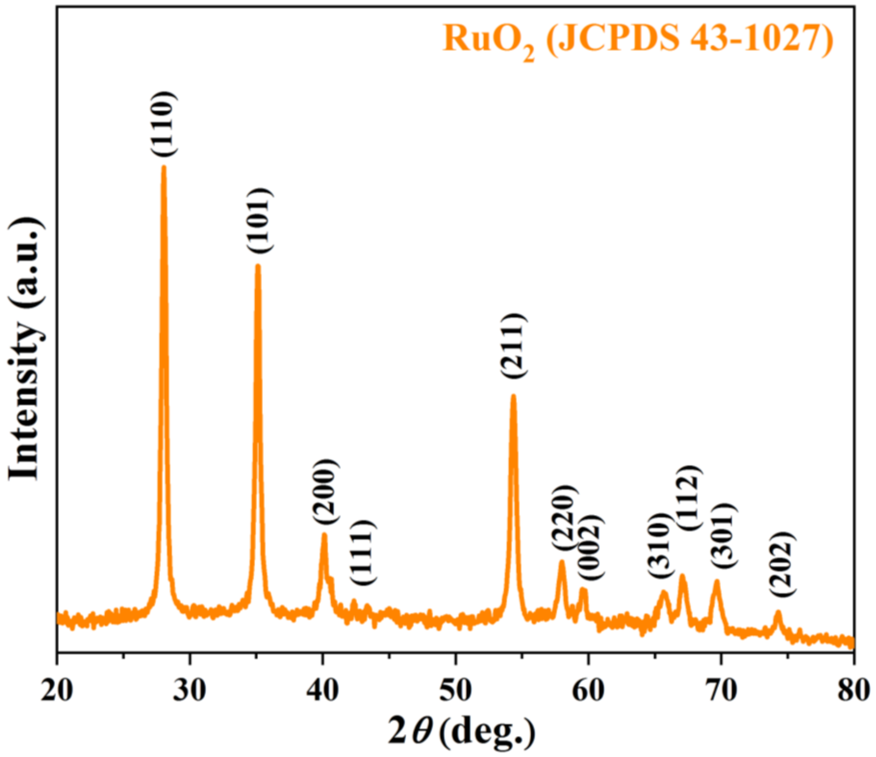

Figure S38. PXRD pattern and the Miller indices of the as-prepared RuO_2 (JCPDS 43-1027).

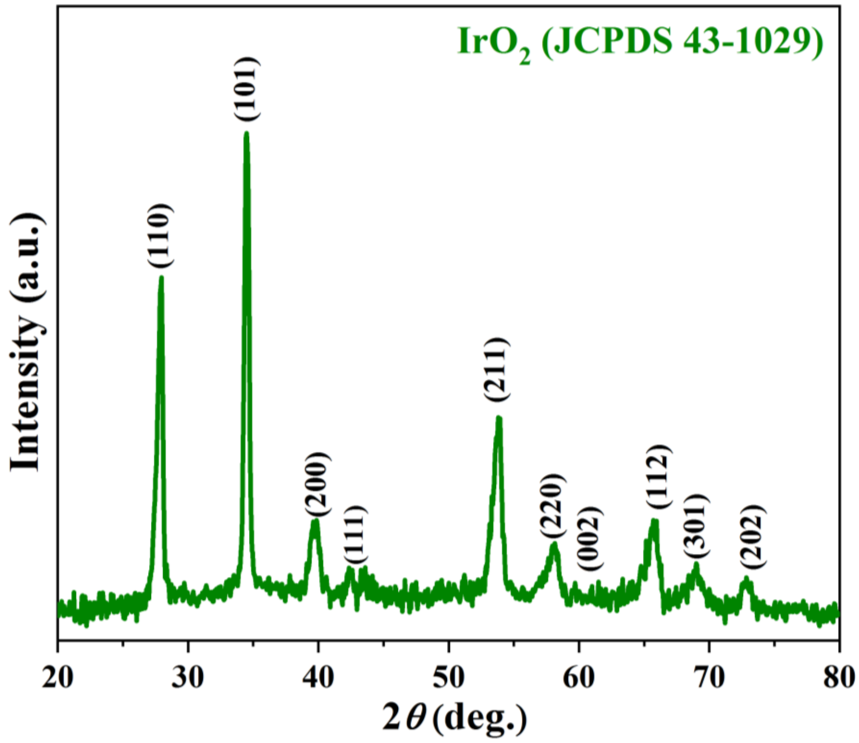

Figure S39. PXRD pattern and the Miller indices of the as-prepared IrO_2 (JCPDS 43-1029).

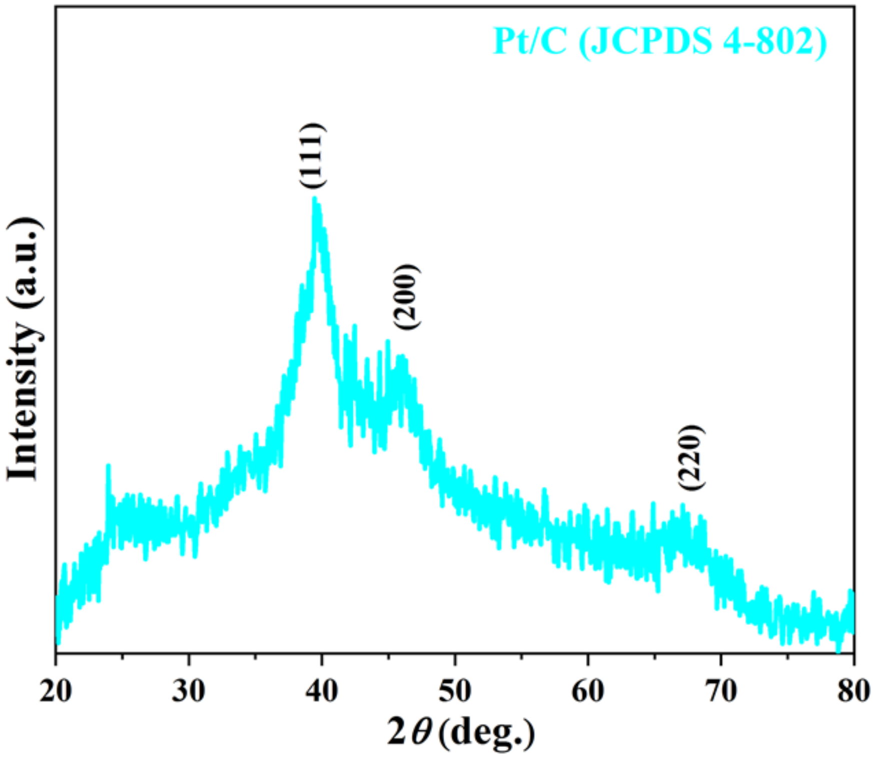

Figure S40. PXRD pattern and the Miller indices of the as-prepared Pt/C (JCPDS 43-802).

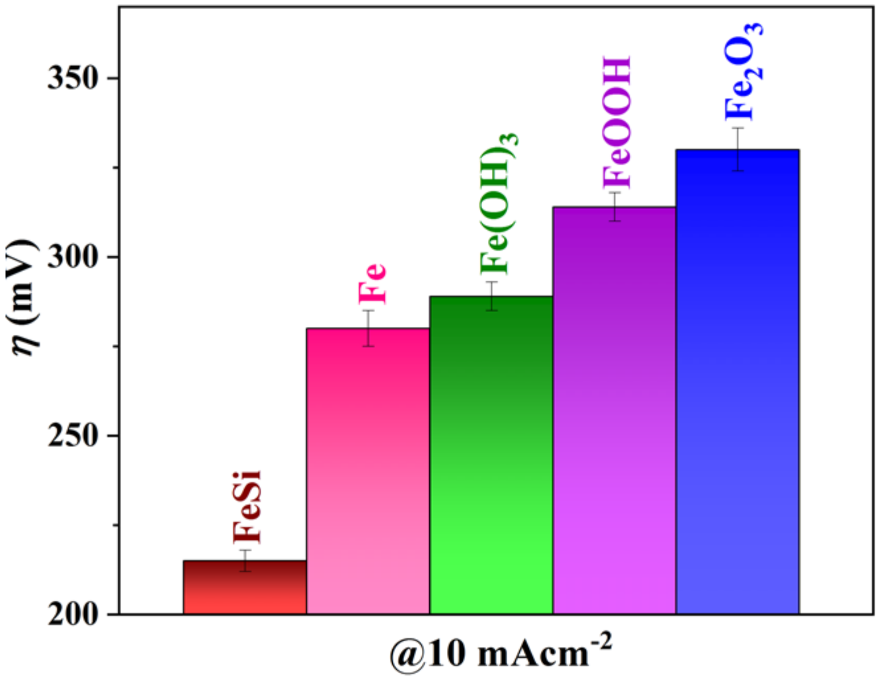

Figure S41. Bar diagram representing the overpotentials (with error bars) of FeSi and other reference Fe-based catalysts on NF at 10 $mAcm^{-2}$ for alkaline OER in identical conditions.

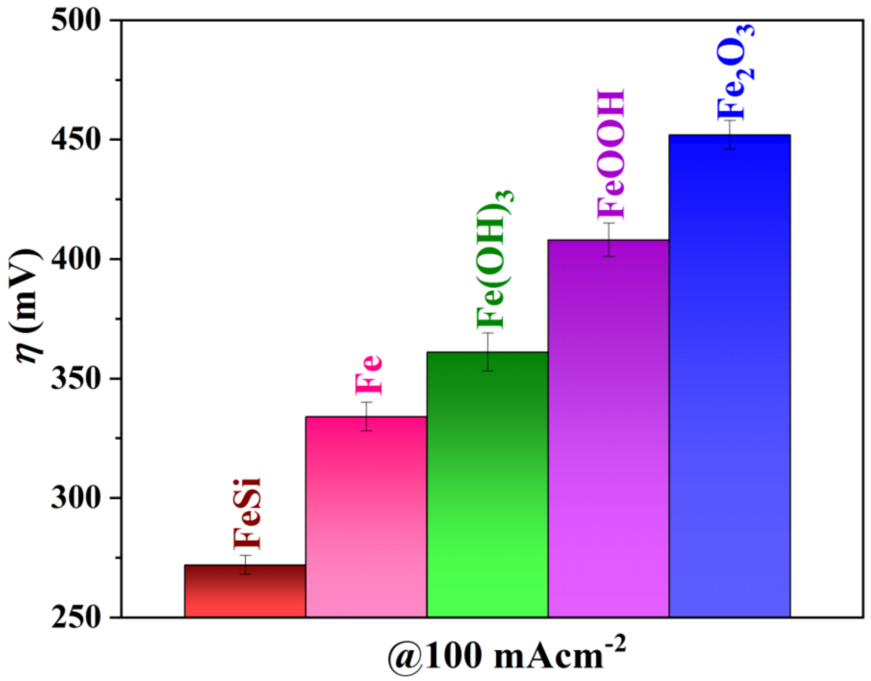

Figure S42. Bar diagram representing the overpotentials (with error bars) of FeSi and other reference Fe-based catalysts on NF at 100 $mAcm^{-2}$ for alkaline OER in identical conditions.

Table S2. Values of the impedance fits of FeSi and the iron based reference compounds deposited on NF.

Material	R_{ct} (Ω)	R_s (Ω)	Q ($F \times s^{(a_2-1)}$)	a_2
NF	47 ± 1	1.3 ± 0.3	0.15 ± 0.01	0.94 ± 0.07
FeSi/NF	0.6 ±0.2	1.2 ± 0.3	0.07 ± 0.04	0.8 ± 0.2
Fe/NF	3.5 ± 1	1.2 ± 0.5	0.07 ± 0.04	0.9 ±0.2
$Fe(OH)_3$/NF	6 ± 5	1.3 ± 0.7	0.088 ± 0.001	0.9 ± 0.1
FeOOH/NF	30 ± 3	1.3 ± 0.6	0.064 ± 0.001	0.86 ± 0.05
Fe_2O_3/NF	43 ± 3	1.3 ± 0.3	0.057 ±. 0.001	0.92 ± 0.06

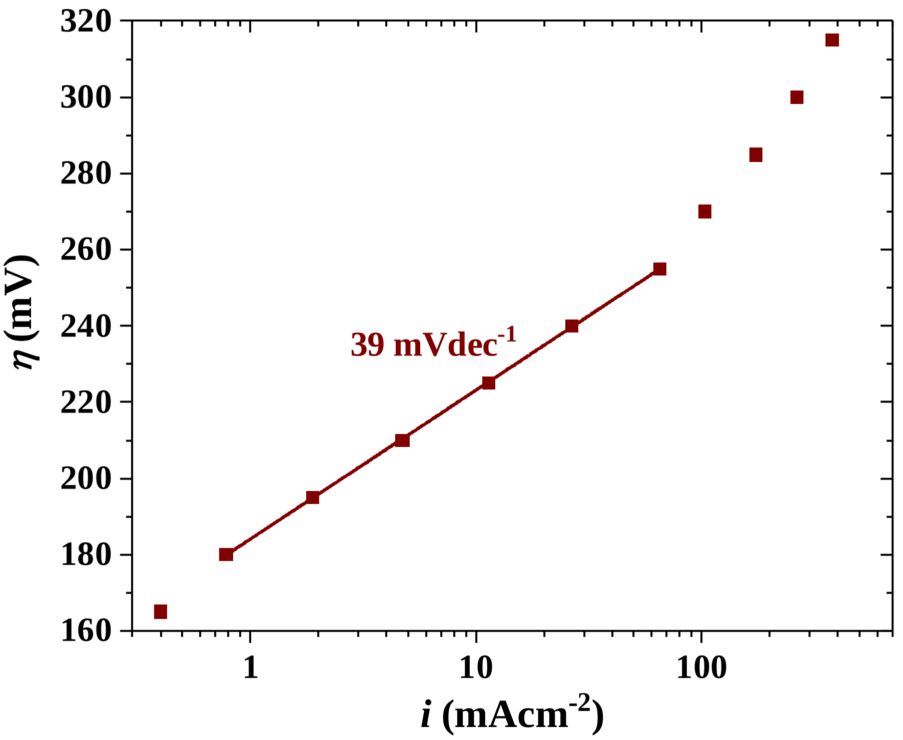

Figure S43. Tafel slope of FeSi/NF obtained from steady-state measurements applying a constant potential for 300 s with a step size of 15 mV.

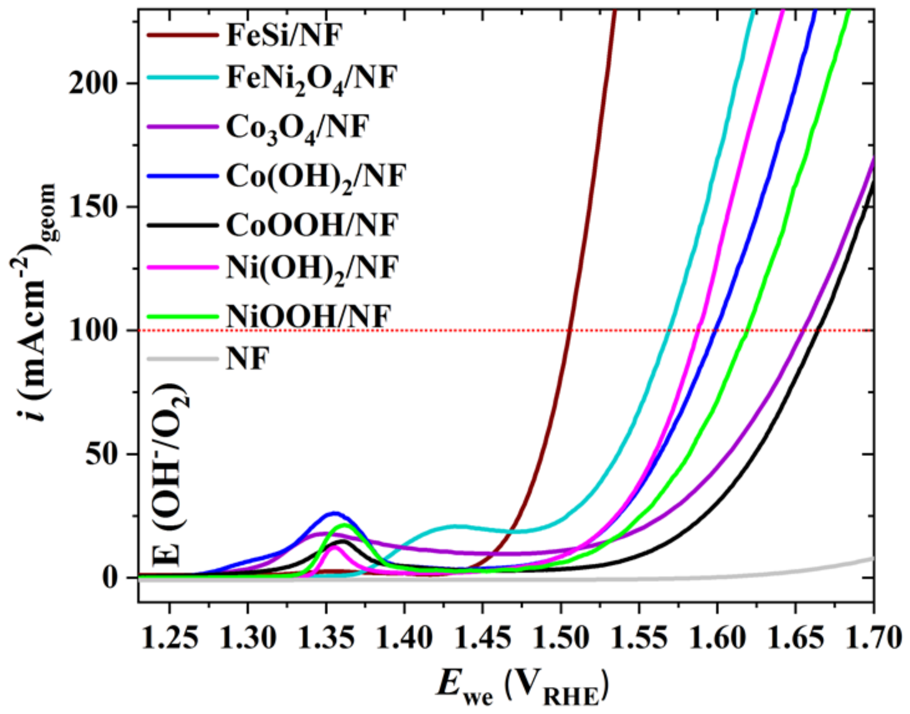

Figure S44. Comparison of LSV curves of FeSi/NF with benchmark non-noble-metal based catalysts on NF at a scan rate of 1 mVs^{-1} in identical conditions, which shows the superior performance of FeSi/NF with respect to the investigated Co- and Fe- and the best active FeNi-catalysts (see Table S3 and S5).

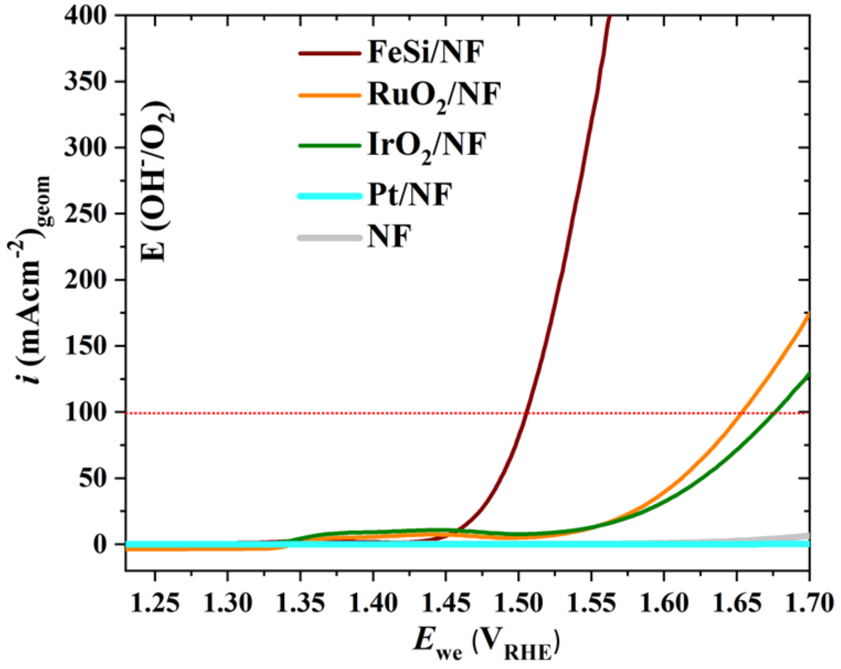

Figure S45. Comparison of LSV curves of FeSi/NF with noble-metal based RuO_2/NF and IrO_2/NF catalysts as well as with Pt/C/NF at a scan rate of 1 mVs^{-1}, which substantiated the better performance of FeSi/NF compared to the other investigated catalysts in identical conditions (see Table S3 and S5).

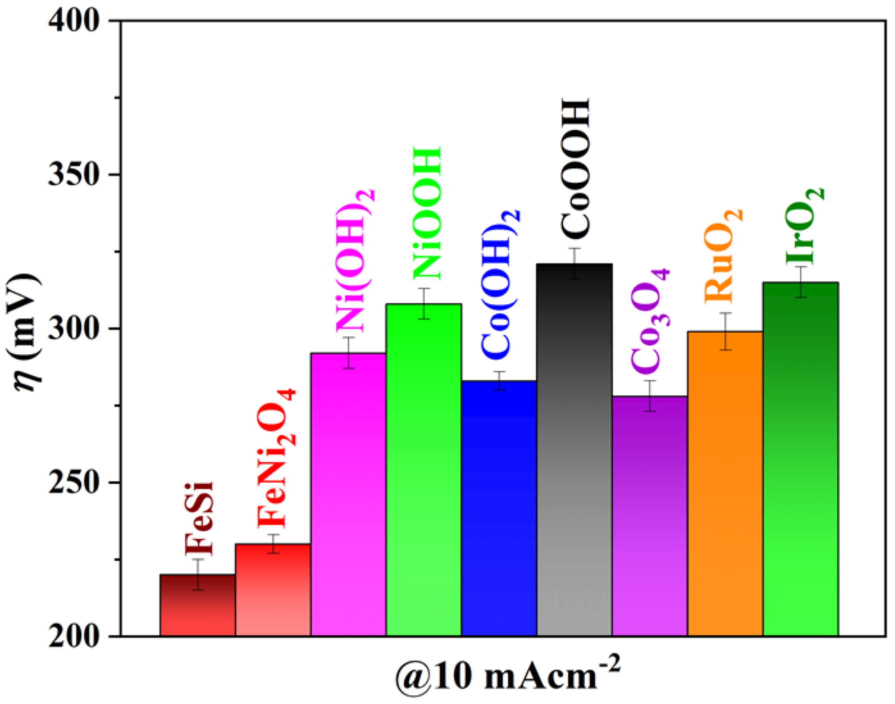

Figure S46. Bar diagram representing the overpotentials (with error bars) of FeSi and highly active transition metal-based as well as state-of-the-art noble metal-based electrocatalysts on NF at 10 mAcm^{-2} for OER in identical conditions.

Table S3. Comparison of OER overpotentials (η) of FeSi with other benchmark catalysts synthesized and tested using our three-electrode set-up in aqueous 1 M KOH in identical conditions.

Catalyst	i (mAcm^{-2})	η (mV) on FTO	η (mV) on NF
**FeSi**	**10**	**415±4**	**218±3**
Fe	10	484±5	279±4
$Fe(OH)_3$	10	578±5	288±3
FeOOH	10	617±7	315±6
Fe_2O_3	10	640±5	331±4
$Ni(OH)_2$	10	380±6	292±5
NiOOH	10	444±6	308±5
$Co(OH)_2$	10	383±5	283±3
CoOOH	10	406±4	321±5
Co_3O_4	10	380±4	278±5
$FeNi_2O_4$	10	334±3	230±3
IrO_2	10	450±3	315±5
RuO_2	10	357±5	299±6
NF	10	-	490±9
FTO	10	-	-

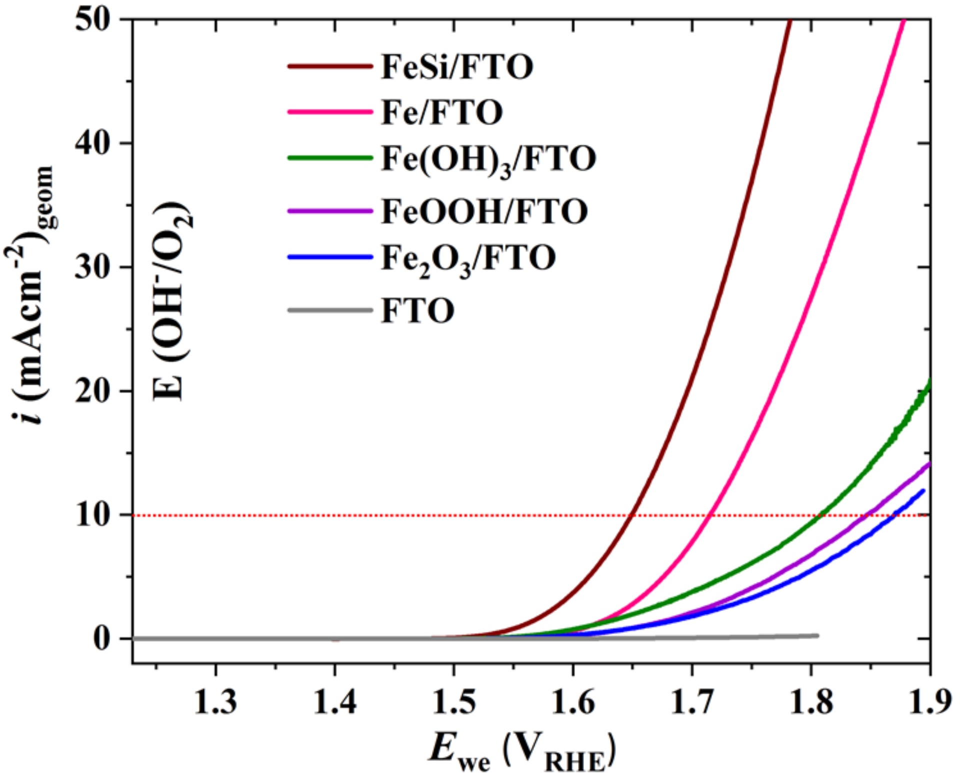

Figure S47. LSV polarization curves of FeSi/FTO, Fe/ FTO, $Fe(OH)_3$/ FTO, FeOOH/ FTO, Fe_2O_3/ FTO and FTO at a scan rate of 5 mVs^{-1} in 1 M aqueous KOH electrolyte. The same trend in OER activity in comparison to NF was observed.

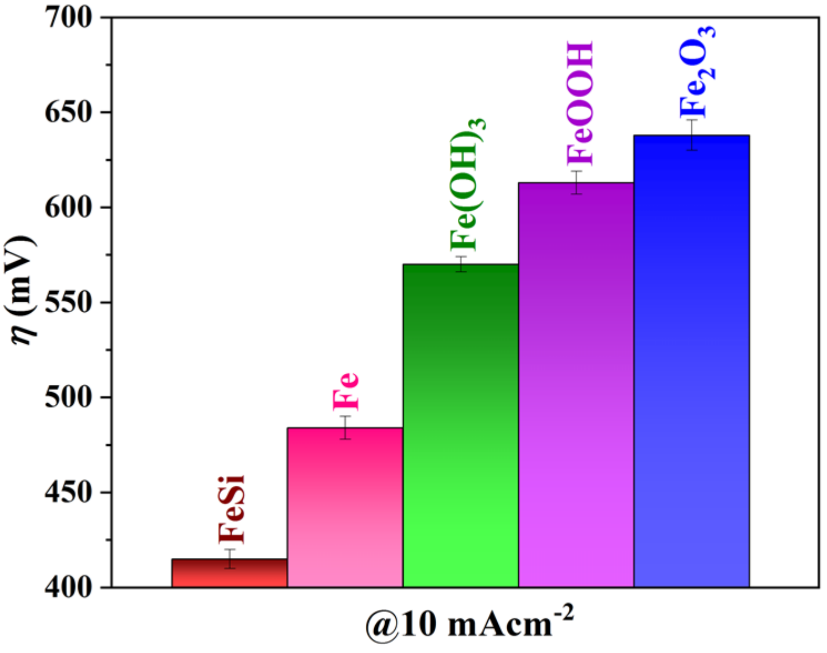

Figure S48. Bar diagram representing the overpotentials (with error bars) of FeSi and other reference Fe-based catalysts on NF at 10 $mAcm^{-2}$ for OER in identical conditions.

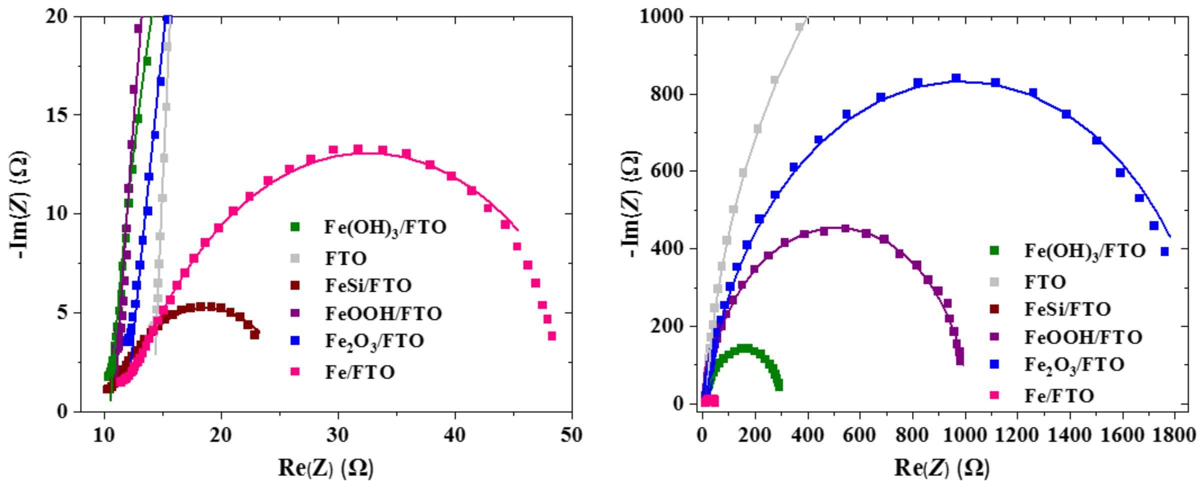

Figure S49. Nyquist plot constructed from EIS experiment of materials deposited on FTO at different scales attained from EIS at an anodic polarization potential of 1.55 V_{RHE}. The straight lines are the fits based on a Randles circuit with a constant phase element (see Table S4 for fitting values).

Table S4. Values of the impedance fit of FeSi and the iron-based reference compounds deposited on FTO.

**Material**	R_{ct} **(Ω)**	R_s **(Ω)**	$Q\ (F \times s^{(a_2-1)})$	a_2
FTO	3626.8 ±0.6	14.3 ±0.2	9.53 ± 0.01	0.96 ±0.05
FeSi/FTO	17 ±3	10 ±1	80 ±10	0.7 ±0.1
Fe/FTO	42 ±2	11.9 ±0.2	60 ±20	0.7 ±0.1
$Fe(OH)_3$/FTO	304 ±1	10.4 ±0.3	15 ± 5	0.93 ±0.05
FeOOH/FTO	1001.9 ±0.4	11.54 ±0.3	14 ± 1	0.95 ±0.05
Fe_2O_3/FTO	1951 ±2	11.4 ±0.3	28.5 ± 0.4	0.91 ±0.05

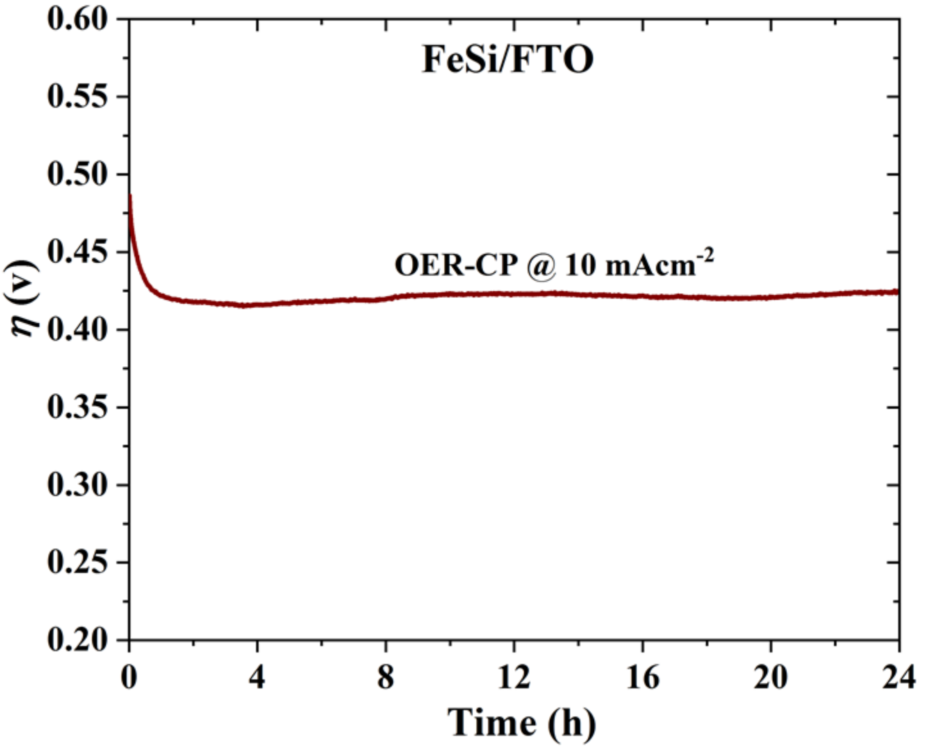

Figure S50. CP response of FeSi/FTO measured at a current density of 10 mAcm^{-2} depicting excellent stability of the electrocatalyst.

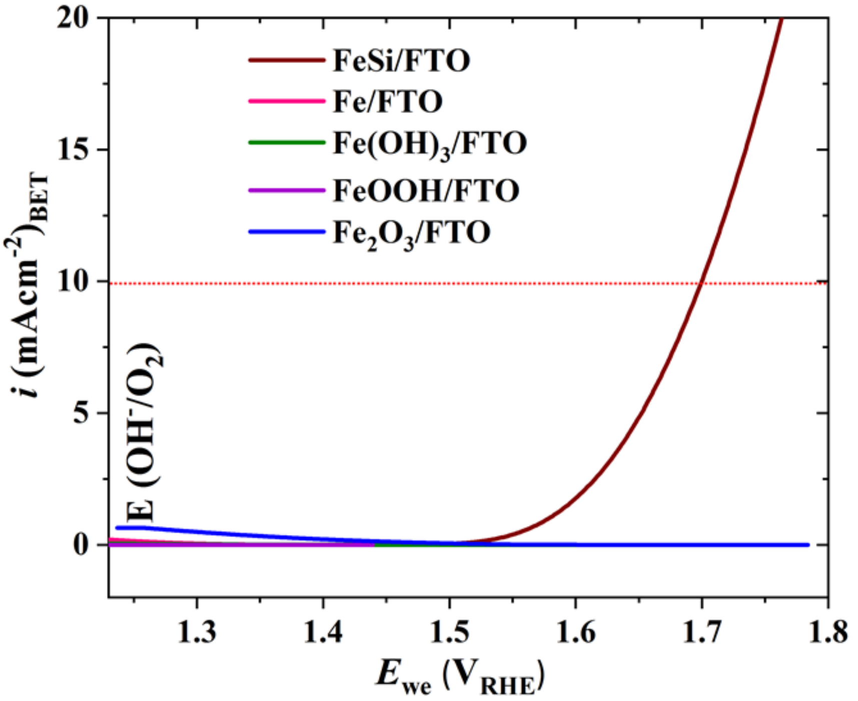

Figure S51. LSV normalized by BET surface of FeSi and other investigated reference Fe-based materials on FTO for alkaline OER.

Table S5. Comparison of OER overpotentials (η) of FeSi with other well-established NiFe-based catalysts in aqueous 1 M KOH.

Catalyst	j (mA·cm^{-2})	Substrate	Stability (h)	η (mV)	Reference
**FeSi**	**10**	**NF**	**24**	**218±3**	**This work**
**FeSi**	**10**	**FTO**	**24**	**415±4**	**This work**
NiFeOOH	10	NiFe	2	240	[17]
NiO_x-Fe	10	NF	18	215	[18]
NiFe LDH	10	NF	13	300	[19]
$Ni_3Fe_{0.5}V_{0.5}$	10	CFP	60	200	[20]
NiFe LDH	10	GC	1	210	[21]
NiFe LDH	10	HOPG	5	260	[22]
NiFe-LDH	10	NF	3	240	[23]
$FeNiO_xH_y$	10	NF	50	206	[24]
CuO@$NiFeOH_x$	10	Cu	16	230	[25]
NiFe-MOF	10	CFP	100	275	[26]
NiFe-LDH	10	CW	-	260	[27]
NiFe-LDH	10	NW	1.5	300	[28]
NiFe alloy	10	GC	2	298	[29]
NiFe-MOF	10	GC	5.5	230	[30]
NiFeTiOOH	10	GC	24	400	[31]
NiFeCo-LDH	10	CFP	10	288	[32]
NiFe alloy	10	GC	20	246	[33]
CNS-NiFe	10	Cu_2O/Cu	24	248	[34]
NiFe-LDH	10	CB	6	236	[35]
NiFe/$CoFe_2O_4$/Co_3S_4	10	CFP	48	233	[36]
$Ni(OH)_2(CO_3)$-Fe^{2+}	10	NF	36	277	[37]
$NiFeNiFe_2O_4$	10	NF	15	316	[38]
$NiFe_xSn$@$NiFe(OH)_x$	10	CFC	11	260	[39]
Co-NiFe-LDH	10	GC	20	278	[40]
$NiFe(Co_3)^{2-}$-LDH	10	NF	20	228	[41]
NiFe	10	NF	30	270	[42]
$NiFe_2O4$/NFM	10	Fe wire	10	234	[43]
NiFe15	10	NiFe sponge	24	280	[44]
NiO/C@NiFe-LDH	10	NF	10	299	[45]
NiFe-LDH@Ni_3S_2	10	NF	6	271	[46]
NiFe-HC	10	GC	12	330	[47]
Ni_1Fe_{10}-LDH@Ni_3S_2	10	NF	12	230	[48]
$NiFeS_2$	10	GC	24	230	[49]
NiFeS	10	GC	6	286	[50]
$Ni_{0.7}Fe_{0.3}S_{2+y}$	10	NF	24	210	[51]
$Ni_{0.7}Fe_{0.3}S_2$	10	NF	14	198	[52]
$Ni_xFe_{1-x}Se_2$	10	GC	24	195	[53]

GC = glassy carbon, CFP = carbon fiber paper, Au = gold, CC =carbon cloth, FTO = fluorine doped tin oxide, HOPG = highly-ordered pyrolytic graphite, NF = nickel foam, CW= carbon wire; CB = carbon black

Calculation of Faradaic efficiency (FE)

The FE of FeSi in 1M aqueous KOH towards OER was measured with FeSi/NF in a closed electrochemical cell (using Pt as a counter electrode). The cell and the electrolyte were first degassed with Argon for 60 min under constant stirring. Afterward, a constant current density of 50 mA cm^{-2} was applied for a specified time. At the end of electrolysis, the gaseous samples were drawn from the headspace by a gas-tight syringe and analyzed by a gas chromatography calibrated for O_2. Each injection was repeated at least three times, and the average value is presented.

The *FE* is calculated based on:

$$FE(O_2,\%) = \frac{4 \times F \times p \times V_{O_2}}{R \times T \times I \times t} \times 100\%.$$

F is the Faraday constant (96485 C/mol), *p* is the pressure (101 kPa), V_{O2} is the evolved volume of oxygen, *R* is the ideal gas constant (8.314 J/(mol K), *T* is the temperature (295.15 K), *I* is the current (the potentiostat was set to 50 mAcm^{-2} but the real current that was measured on average by the potentiostat was only 49.3 mAcm^{-2}), and *t* is the period of electrolysis (420 s).

Table S6. Calculation of Faradaic efficiency.

	j(mAcm^{-2})	*t* (s)	V_{O2} (mL)	*FE* (O_2,%)
**FeSi**	49.3	420	1.25± 0.05	96±4

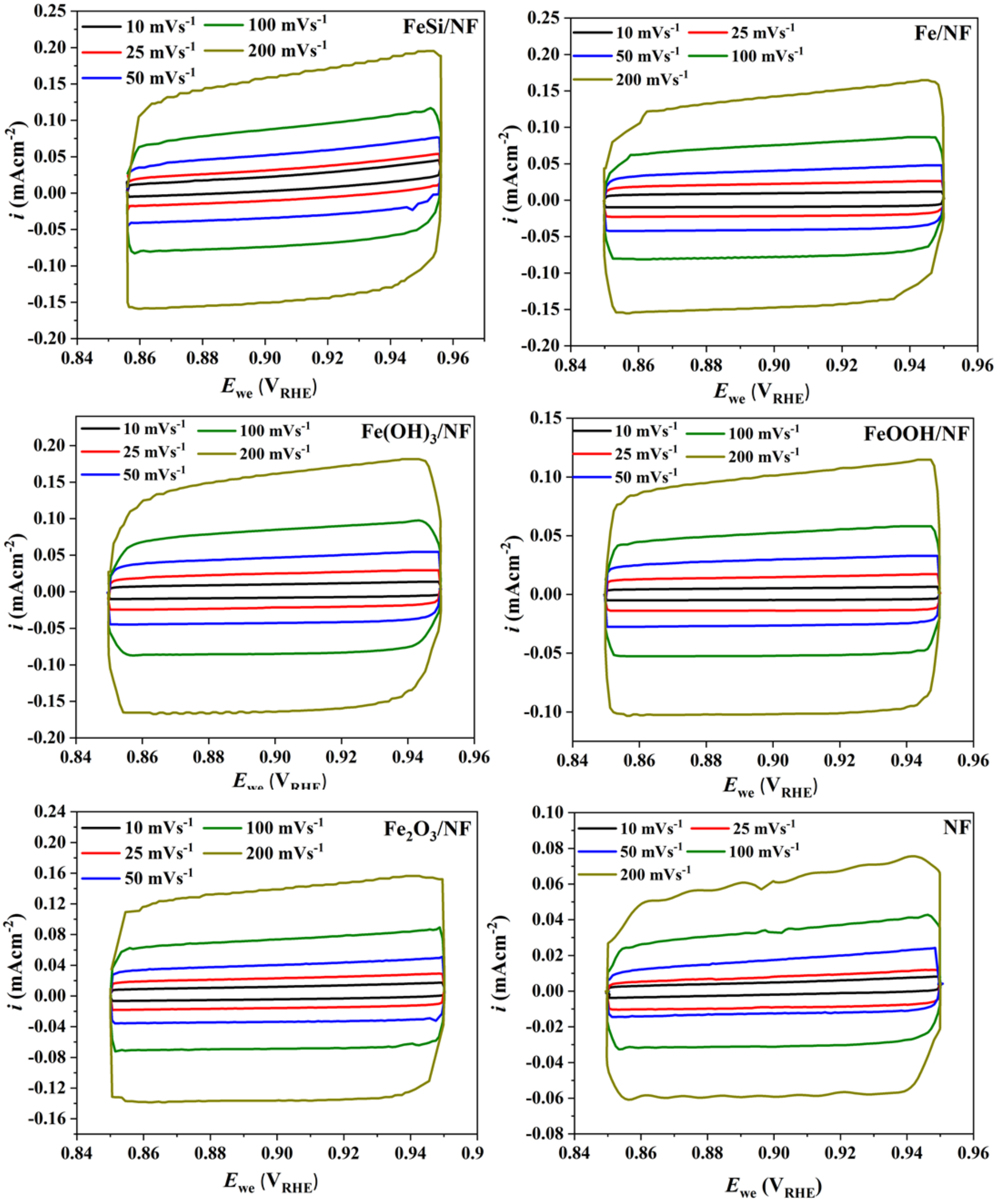

Figure S52. C_{dl} analysis of FeSi/NF, Fe/NF, $Fe(OH)_3$/NF, FeOOH/NF, Fe_2O_3/NF, and NF. The CV scans were conducted in a non-Faradaic potential range in 1 M aqueous KOH solution at a sweep rate of 10 mVs^{-1}, 25 mVs^{-1}, 50 mVs^{-1}, 100 mVs^{-1}, and 200 mVs^{-1}. Half of the differences in current density variation ($\Delta J = (J_{cathodic}$-$J_{anodic})/2$) at a potential of 0.90 V_{RHE} were plotted against the scan rate and fitted by linear regression allowing the determination of the C_{dl}.[13]

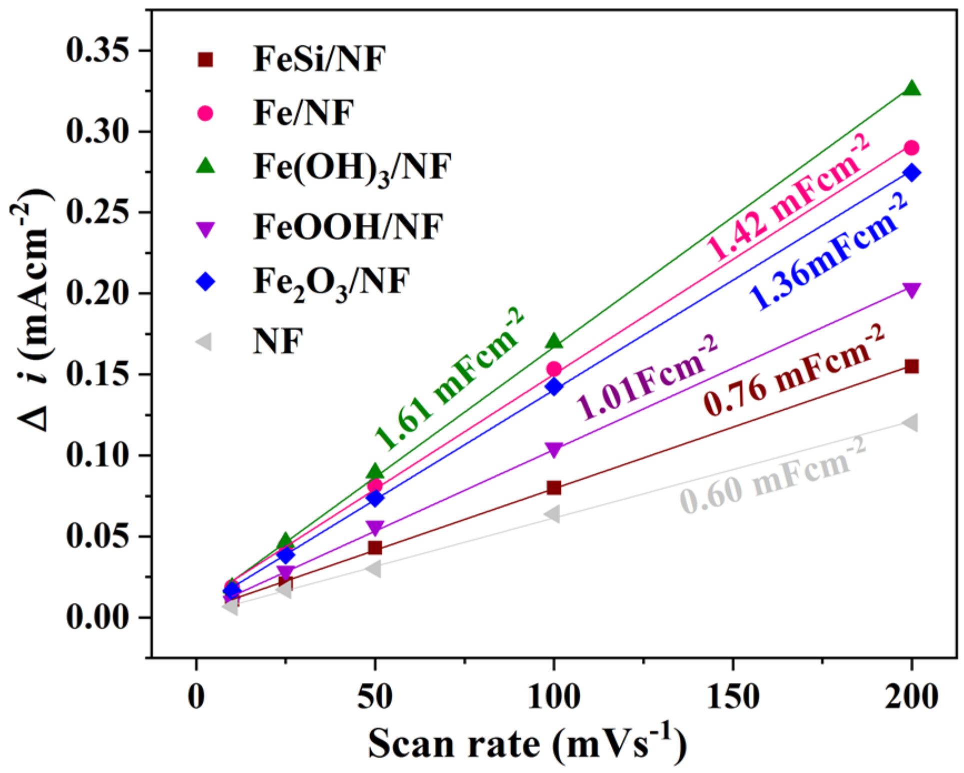

Figure S53. The C_{dl} plots of FeSi/NF, Fe/NF, $Fe(OH)_3$/NF, FeOOH/NF, Fe_2O_3/NF and NF. Current density differences at 0.90 V_{RHE} were plotted against the scan rate and fitted by linear regression to obtain the C_{dl}.[13]

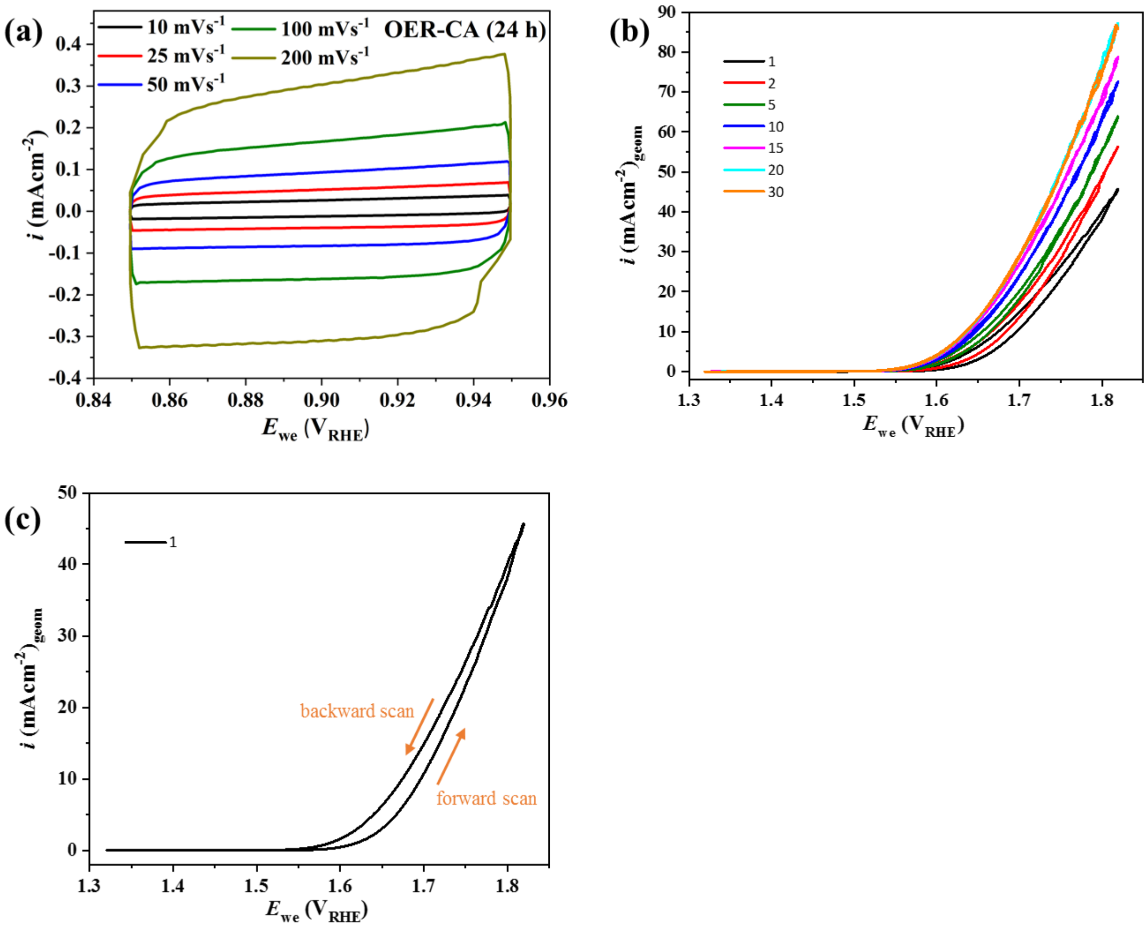

Figure S54. (a) C_{dl} analysis of FeSi/NF after 24 h of OER-CP at 10 mA/cm^2. The CV scans of FeSi/NF OER-CP (for as-deposited see Figure S35-S36) were conducted in a non-Faradaic potential range in 1 M aqueous KOH solution at a sweep rate of 10 mVs^{-1}, 25 mVs^{-1}, 50 mVs^{-1}, 100 mVs^{-1}, and 200 mVs^{-1}. Half of the differences in current density variation (ΔJ = ($J_{cathodic}$-J_{anodic})/2) at a potential of 0.90 V_{RHE} was plotted against the scan rate and fitted by linear regression allowing the determination of the C_{dl}.[13] A more than four times larger C_{dl} value, 3.20 mFcm^{-2}, was observed for FeSi/NF OER-CP than for FeSi/NF (0.76 mFcm^{-2}). This can be attributed to significant surface structural transformations and indicates the presence of higher amounts of accessible active sites (area) for OER.[10c,54] (b) CV of FeSi/FTO at a scan rate of 5 mV/s showing the activation of the material during the first 20 scans. (c) First CV revealing the absence of a reversible redox feature and that the backward scan yields a larger current response than the forward scan due to the activation of the catalyst during the forward scan.

Table S7. Specific resistivity ρ (Ω cm) of FeSi/FTO, Fe/FTO, $Fe(OH)_3$/FTO, FeOOH/FTO, Fe_2O_3/FTO, and FeSi after OER-CP (24 h) deposited on FTO, compared to the bare FTO recorded by a four-point probe resistivity measurement. The mass loading (2 mg/cm^2) was higher than in all other measurements to make sure that the FTO is fully covered by the iron-based materials. The errors are around 30% of the absolute values. The high resistivity of the Fe films is caused by the severe degree of oxidation and the small particle size and can be seen in Figure S15-S20.

Electrodes	ρ (Ω cm)
**FeSi/FTO**	**6.2**
**FeSi/FTO after OER-CP**	**100**
Fe/FTO	1×10^3
$Fe(OH)_3$/FTO	6×10^3
FeOOH/FTO	2×10^4
Fe_2O_3/FTO	2×10^4
FTO	7.1

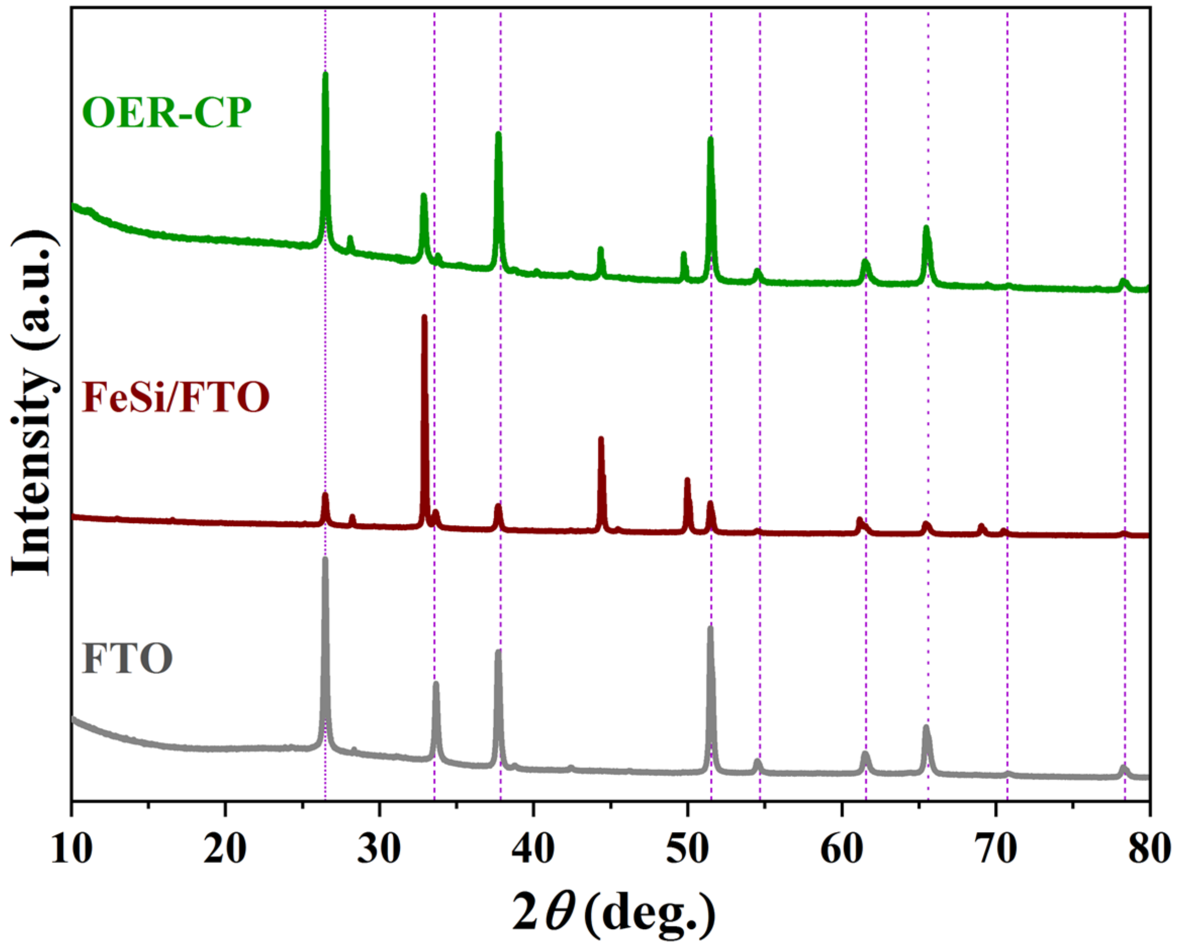

Figure 55. PXRD of a post-OER-CP (24 h) and an as-deposited FeSi/FTO film along with bare FTO as a reference. No significant difference in the reflections between as-deposited and OER-CP was observed suggesting that a significant amount of the material after OER is still intact. However, the possibility of the surface transformation of FeSi to amorphous FeO_xH_y (unidentifiable by X-ray diffraction) cannot be neglected. Therefore, the OER-CP films were further systematically characterized by SEM, TEM, EDX mapping, ICP-AES, XPS, *quasi in situ* and *ex situ* RR, and *quasi in situ* XAS analysis (see main text).

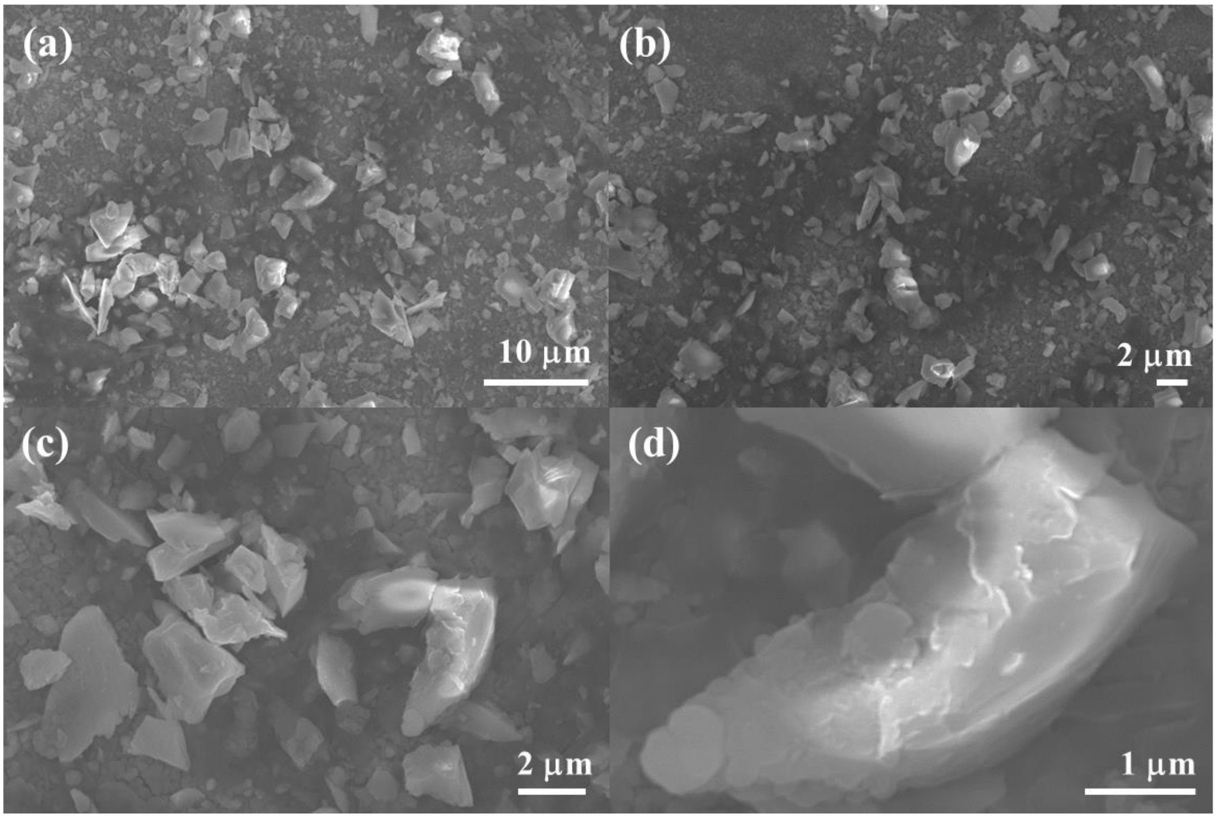

Figure S56. SEM images (a-d) of FeSi/FTO films after OER-CP (24 h) at various magnifications. The SEM images clearly indicated t a corroded surface in comparison to the initial FeSi phase.

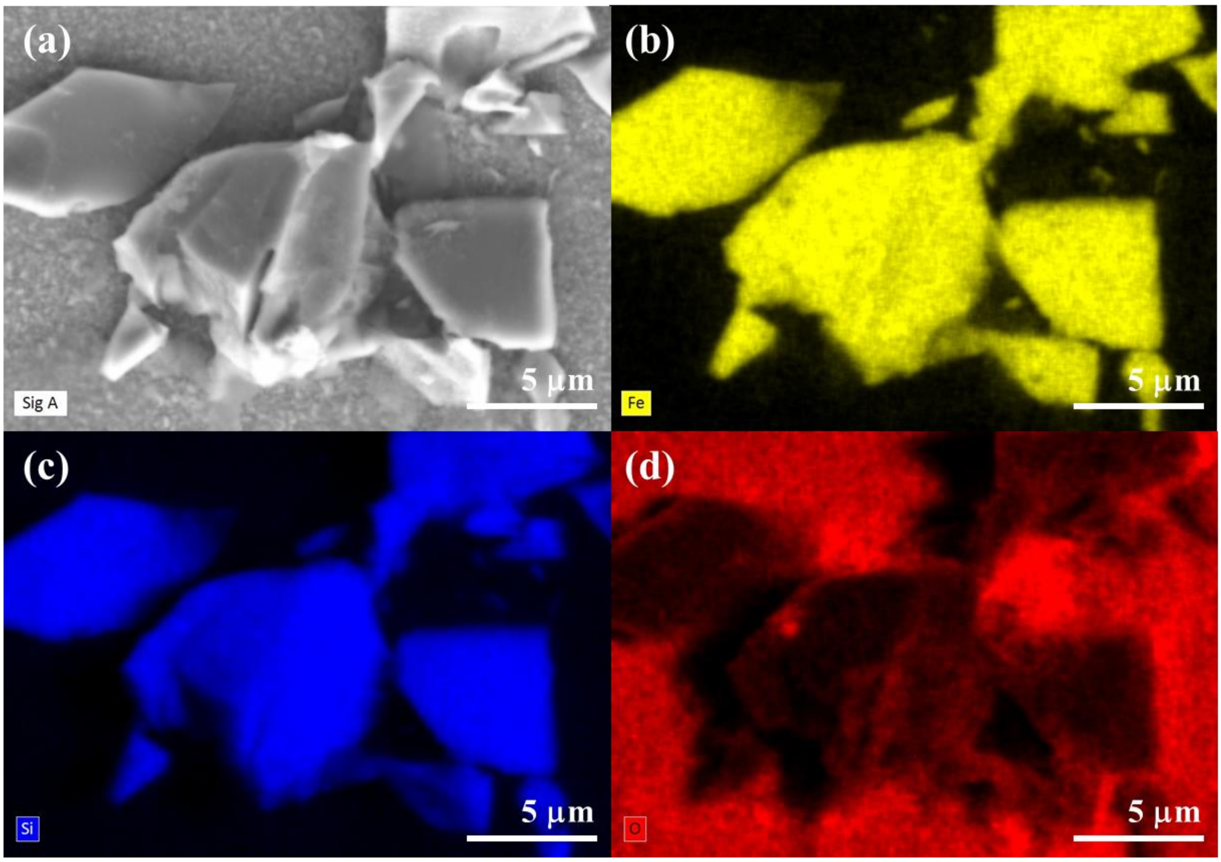

Figure S57. SEM image (a) and the respective EDX mapping of selected FeSi particles after 24 h OER-CP (b-d). The iron (b, yellow) and Si (c, blue) were still found to be homogeneously distributed on the FeSi particles while some part of the surface of the particles showed strong oxygen (d, red) incorporation. The results obtained here suggested the formation of a Fe-oxide(hydroxide) phase, which is an active OER structure in the literature for enhanced catalytic activity.[55]

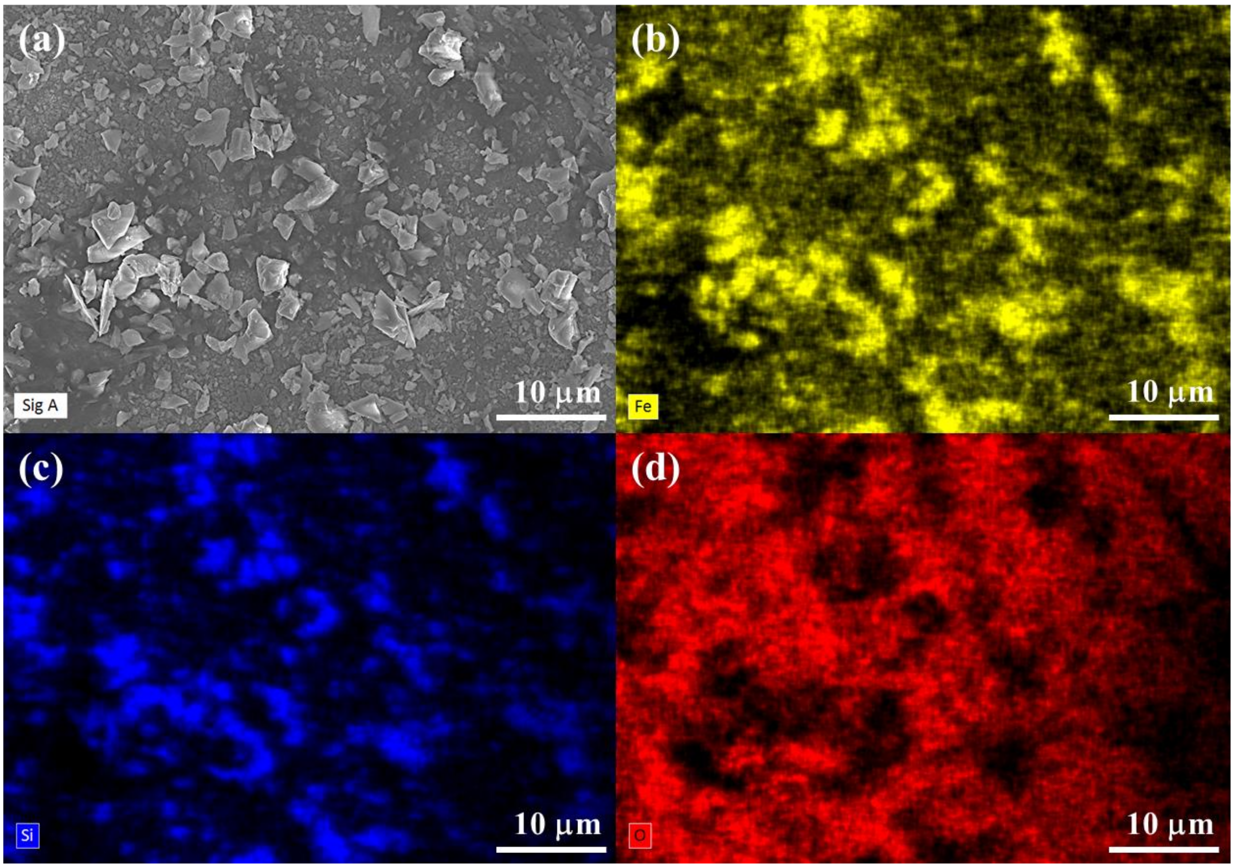

Figure S58. SEM image (a) and the respective EDX mapping of FeSi film after OER-CP (24 h) (b-d). Similar to the mapping on the particles (see Figure S47), the film also showed a homogenous distribution of Fe (yellow) and Si (blue) with the incorporation of O (red) suggesting the formation of a $FeO_x(H)$ phase in alkaline OER.[5,55]

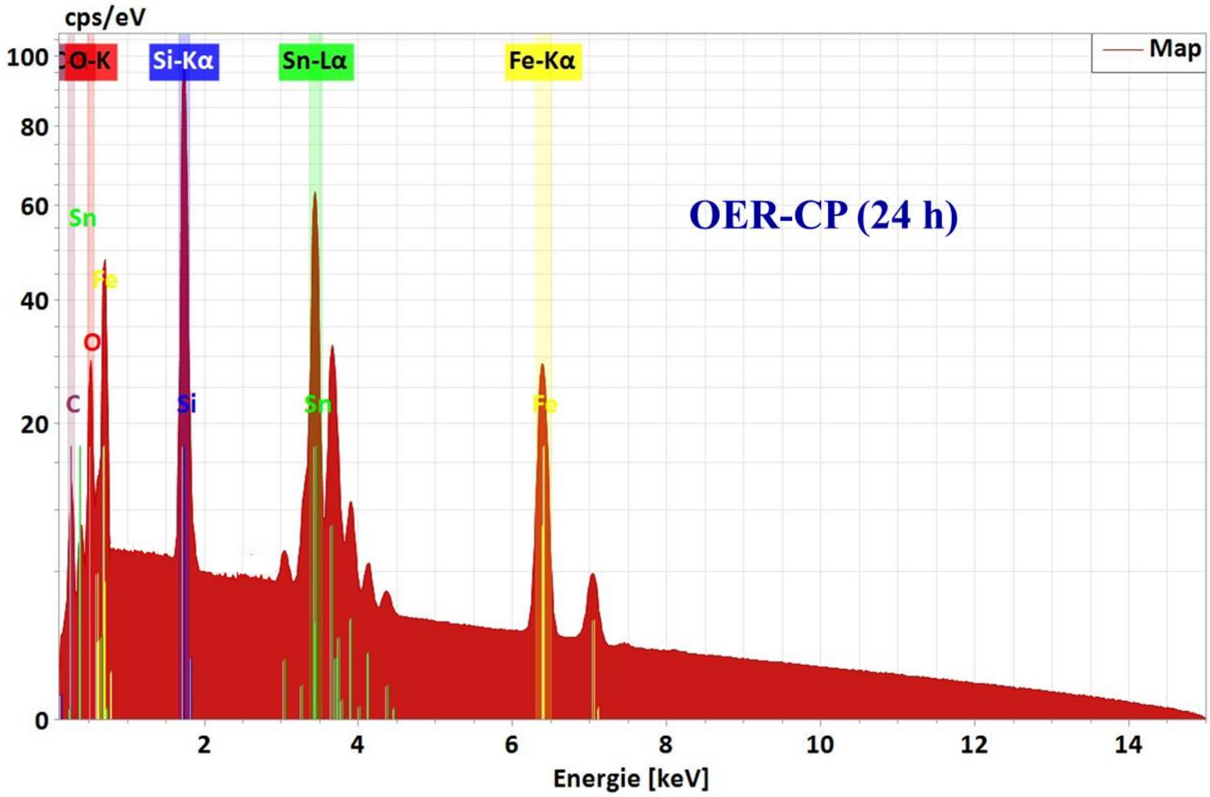

Figure S59. The EDX mapping spectrum of FeSi/FTO film after OER-CP (24 h). Sn signals arise from the FTO glass substrate electrode. Interestingly, the composition of Fe:Si was found to be 1: 0.72 which hinted the loss of Si from the surface of the particles under strongly alkaline OER conditions. This was also confirmed by ICP-AES analysis (see Table S1).

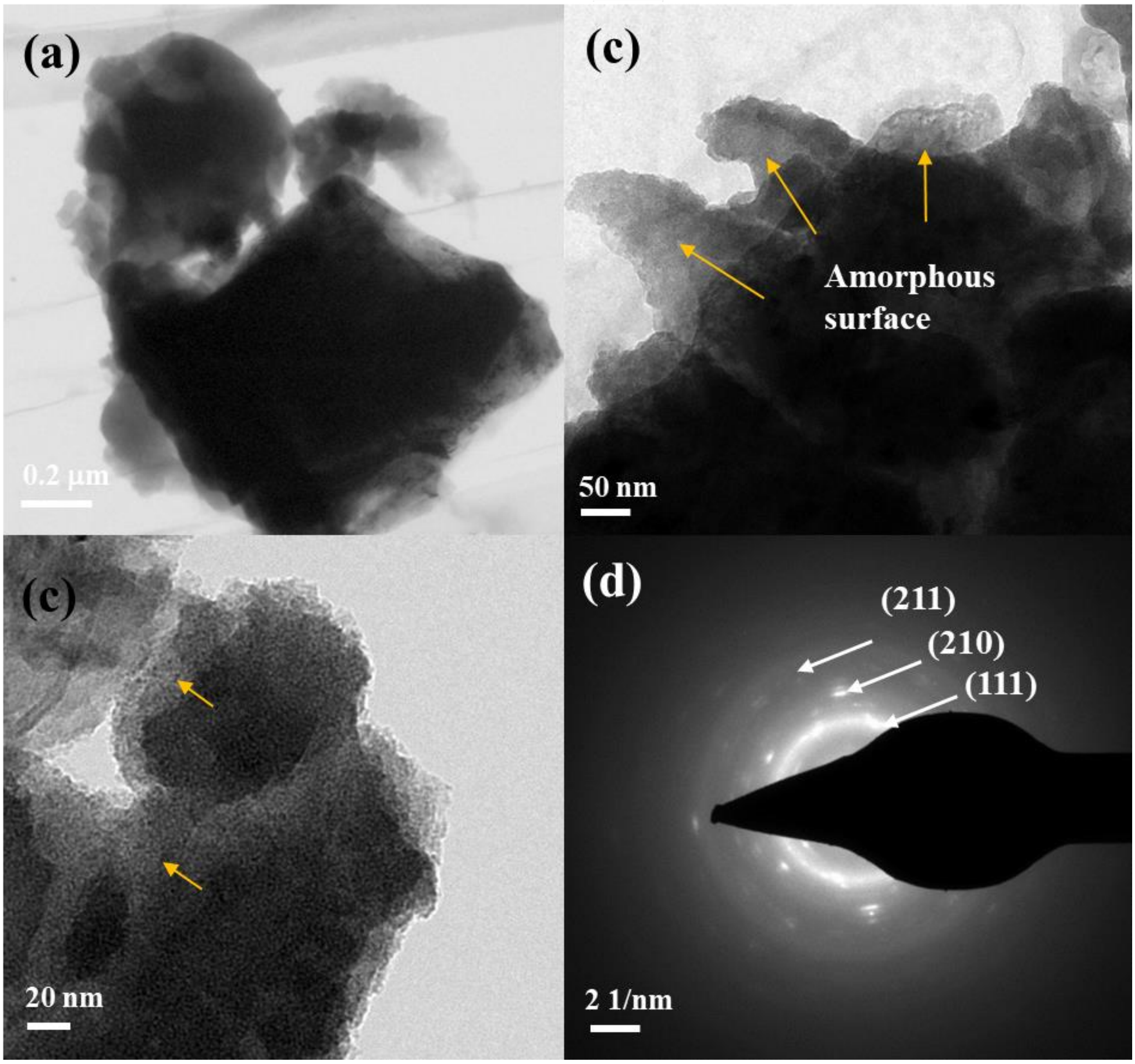

Figure S60. (a-c) TEM images at various magnifications and (d) SAED pattern of FeSi/FTO after OER-CP (24 h). The nanoparticles were first separated from the FTO by ultrasonication and placed on the TEM grid. The TEM image (a) displays the FeSi particles after OER-CP whereas the image (b and c) confirms the formation of amorphous surface structure. The SAED pattern (d) produced diffraction spots and rings of (211), (210), and (111) corresponding to the FeSi structure (JCPDS 38-1397).

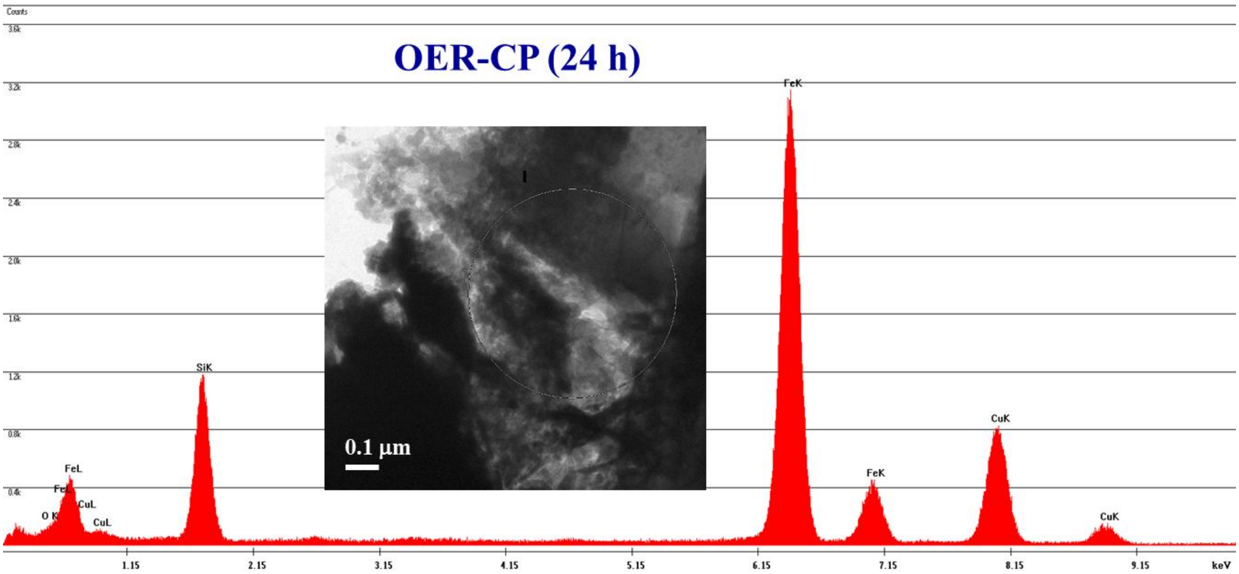

Figure S61. The EDX analysis of FeSi/FTO (on isolated particles; see inset) showing the presence of Fe and Si. The peaks for copper and carbon can be unambiguously correlated to the TEM grid (carbon film on 300 mesh Cu-grid).).

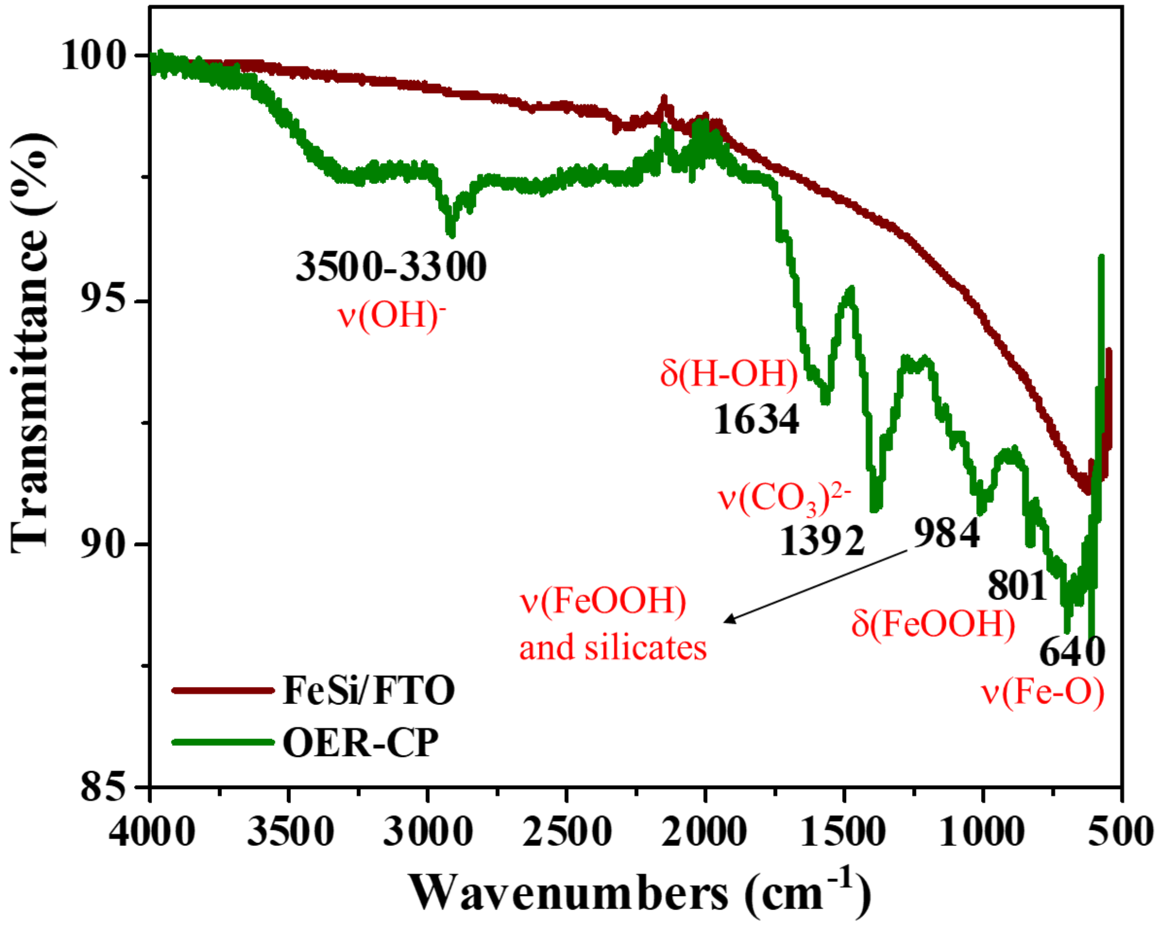

Figure S62. The FT-IR spectra of as-deposited FeSi/FTO (purple curve) and films after OER-CP (24 h) (green curve). After the OER, a transformation of the initial FeSi phase was observed with the appearance of some new IR bands. The broad band around 3300-3500 cm^{-1} could be assigned to stretching vibrations of interlayer water molecules whereas the band at 1634 cm^{-1} could be ascribed to the bending vibration of H_2O and structural OH groups.[23] The band at 1392 cm^{-1} can be attributed to carbon-oxygen stretching vibrations in $(CO_3)^{2-}$ anions,[56] the $(CO_3)^{2-}$ arises from dissolved CO_2 in the KOH electrolyte and probably resided in the interlayer spacing. The broad band at 984 cm^{-1} can be corroborated to stretching vibrations of an iron oxyhydroxide phase.[57] Furthermore, previous FT-IR investigations of potassium silicate solutions show various overlapping features in this region.[58] The band at 801 cm^{-1} is consistent with bending iron oxyhydroxide vibrations.[57] The band at 640 cm^{-1} is due to the stretching Fe-O present in the iron oxyhydroxide structure.[57] The FT-IR results clearly suggest that the surface of FeSi was partially transformed to iron oxyhydroxide, which is in its amorphous form (as deduced from TEM and X-ray diffraction studies) and also consistent with the literature reported iron oxyhydroxide structure for alkaline OER catalysts.[54b,59]

.

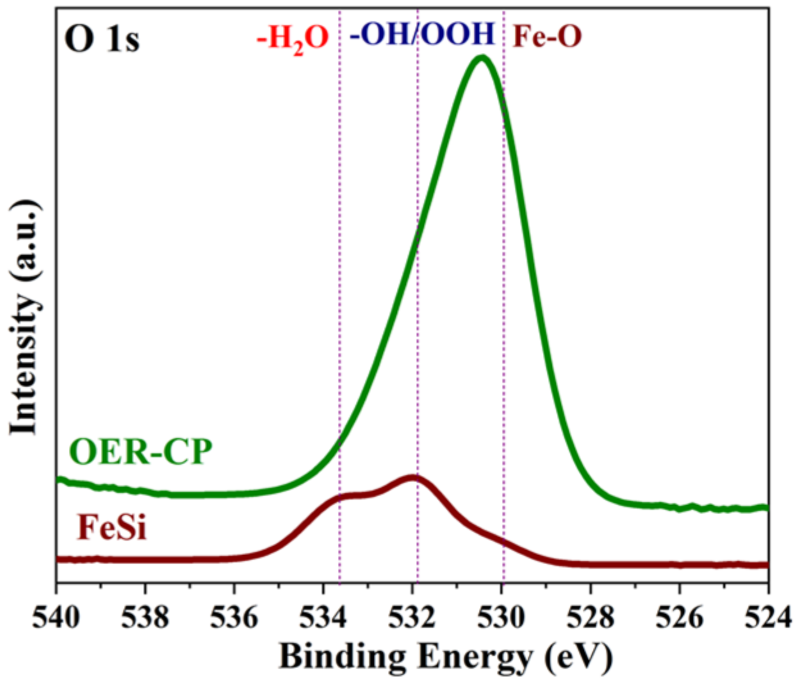

Figure S63. High-resolution deconvoluted O 1s XPS spectrum of FeSi as-prepared (purple) and after OER-CP (24 h, green). The O 1s spectrum of the as-prepared sample shows a minute amount of oxygen with peaks responsible for hydroxylation and adsorbed water. However, the O 1s of OER-CP display peaks exhibiting a large amount of hydroxylation (OH/OOH).[54b,60]

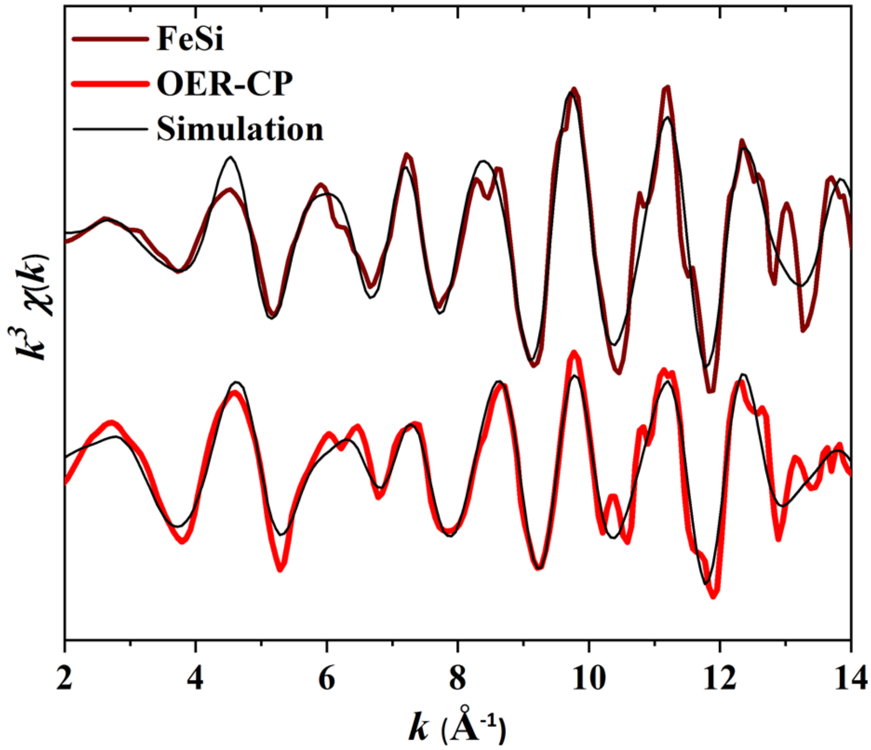

Figure S64. k^3-weighted experimental EXAFS spectra of as-prepared FeSi and post OER-CP (24 h) on FTO substrate. Thin black lines represent the simulation of the experimental data. The simulation parameters are given in Tables S8 and S9.

Table S8. Simulation parameters for as prepared FeSi powder. *N* is the *EXAFS* coordination number, *R* the absorber-backscatter distance, and σ the Debye-Waller parameter. In all refinements, the amplitude reduction factor ($s_o^2(k)$) was 0.7. The Debye-Waller factors were restricted as joined-fit to reduce the number of variables. The errors represent the 68% confidence interval of the respective fit parameter. The values marked with * were fixed to crystallographic values and not fitted (FeSi phase JCPDS 38-1397). The obtained R_f value of the fit is 18.2%, which corresponds to a mean deviation between data and simulation of 18.2% in the distance range of interest (1 - 4 Å of reduced distance).[31]

shell	*N*	error	*R* [Å]	error [Å]	σ [Å]	error [Å]
Fe-Si	4*		2.31	0.01	0.061	0.004
Fe-Si	3*		2.60	0.02	0.061	
Fe-Fe	6*		2.72	0.01	0.068	0.004
Fe-Si	3*		3.67	0.02	0.061	
Fe-Fe	6*		4.05	0.02	0.068	
Fe-Si	3*		4.10	0.05	0.061	

Table S9. Simulation parameters for FeSi in situ freeze quenched after 24 h at 420 mV overpotential yielding a current density of around 10 mA/cm². *N* is the *EXAFS* coordination number, *R* the absorber-backscatter distance, and σ the Debye-Waller parameter. In all refinements, the amplitude reduction factor ($s_o^2(k)$) was 0.7. The Debye-Waller factors were fixed to reasonable numbers for the Si- and Fe-shells and only refined for the O-shell in order to reduce the number of variables. The errors represent the 68% confidence interval of the respective fit parameter. The first six columns belong to a the FeSi phase (JCPDS 38-1397) and the values marked with * were fixed to the crystallographic data and not fitted. Additionally, a second phase was used for the simulation. This phase represents the newly formed amorphous FeO_xH_y phase. The phase is represented by a Fe-O distance correlating to Fe^{III} octahedrally coordinated by oxygen.[61] The other two distances belong to edge and corner sharing [FeO_6] octahedra.[61] The EXAFS coordination numbers of the two phases reveal a 1 to 1 ratio of the crystalline FeSi to the amorphous FeO_xH_y phase. The obtained R_f value of the fit is 10.8%, which corresponds to a mean deviation between data and simulation of 10.8% in the distance range of interest (1 - 4 Å of reduced distance).[31]

shell	*N*	error	*R* [Å]	error [Å]	σ [Å]	error [Å]
Fe-Si	2*		2.33	0.01	0.045	
Fe-Si	1.5*		2.54	0.01	0.045	
Fe-Fe	3*		2.75	0.01	0.050	
Fe-Si	1.5*		3.65	0.05	0.045	
Fe-Fe	3*		4.06	0.02	0.050	
Fe-Si	1.5*		4.11	0.06	0.045	
Fe-O	2.9	0.6	1.94	0.01	0.067	0.015
Fe-Fe	1.3	0.3	3.02	0.01	0.050	
Fe-Fe	0.5	0.4	3.47	0.06	0.050	

Table S10. Comparison of FeSi with $FeSn_2$ which are both intermetallic Fe containing OER catalysts.

	**FeSi**	**$FeSn_2$**
**Morphology**	Irregularly shaped particles (1-40 μm large)	Nanoparticles of around 50 nm diameter
**Abundance of 2nd element in earth's crust [ppm]**	270 000	2.2
**Catalyst loading on nickel foam**	0.8 mg/cm^2	1.6 mg/cm^2
**η at 100 mA/cm^2 geometric [mV]**	275	257
***i* at η = 300 mV BET normalised [mA/cm^2]**	122	13
**η at 100 A/g mass catalyst normalized [mV]**	269	278
**Tafel slope [mV/dec]**	39	33
**In situ formed phase**	New, highly disordered, and pXRD amorphous phase consisting of [FeO_6] octahedra which are connected via edge (70%) and corner sharing (30%), the phase is doped with silicon(II) and (IV) (Fe to Si around 1:0.4)	Crystalline α-FeOOH
**In situ spectroscopy**	Raman and X-ray adsorption including XANES and EXAFS	none
**High temperature and current density testing**	Includes testing 500 $mAcm^{-2}$ at 1.51 V_{RHE} and 65 °C	none

**Main arguments for increased activity**	(i) Fe acting as the active site<br>(ii) due to the amorphous nature and Si leaching more than only the near-surface Fe sites are OER active<br>(iii) sufficient conductivity of the in situ formed Fe(III) probably due to remaining Si(II) and (IV) species<br>(iv) more terminal Fe edge sites due to the amorphous nature<br>(v) the remaining conducting FeSi core might help in electron transport to the Fe(III) sites; however, the newly formed oxidic Fe(III) phase is comparably thick and thus must also be able to conduct electrons sufficiently	(i) Fe of α-FeOOH acting as the active site<br>(ii) Conducting $FeSn_2$ core which helps enables electron transport to the active α-FeOOH phase<br>(iii) Small particle size resulting in a large surface area

Table S11. Comparison of OER potentials vs RHE and the overpotentials of FeSi on NF tested at elevated temperatures using a three-electrode set-up in aqueous 1 M KOH conditions. E_{rev} was calculated based on $E_{rev} = 1.5184 - 1.5421 \times 10^{-3} \times T - 9.523 \times 10^{-5} \times T \times \ln T - 9.84 \times 10^{-8} \times T^2$.[63]

FeSi/NF	i (mAcm^{-2})	Potential (V_{RHE})	E_{rev} (V)	η (mV)
25 °C	10	1.449	1.230	219
	100	1.511		281
	500	1.584		354
35 °C	10	1.439	1.221	218
	100	1.500		279
	500	1.558		337
45 °C	10	1.428	1.213	215
	100	1.488		275
	500	1.542		329
55 °C	10	1.413	1.204	209
	100	1.475		271
	500	1.519		315
65 °C	10	1.398	1.196	202
	100	1.461		265
	500	1.504		308

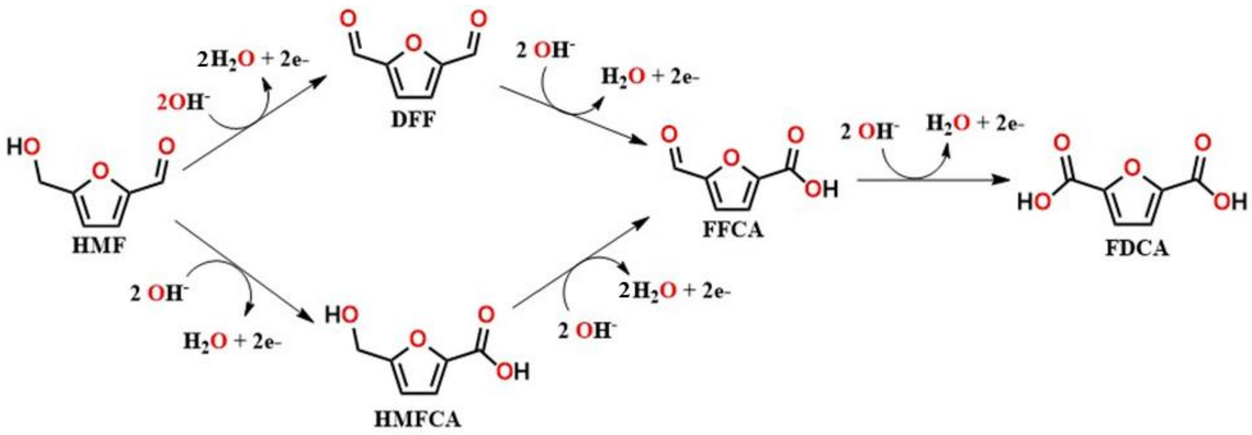

Scheme S1. Two plausible pathways of HMF to FDCA under electrocatalytic oxidation.[62]

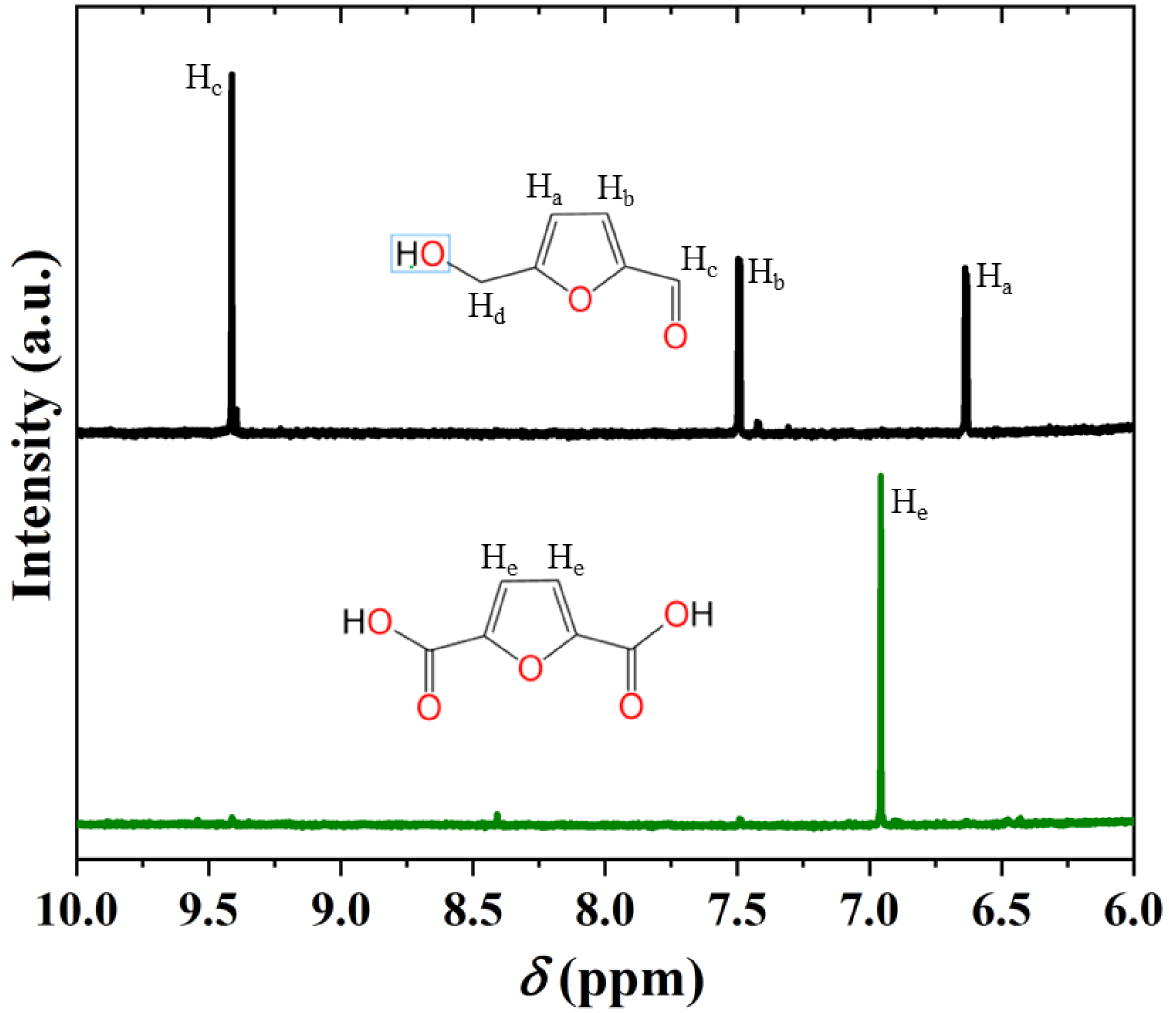

Figure S65. ^{1}H NMR spectrum of 5-hydroxymethylfurfural (HMF, black line) before the start of the reaction and after a CP experiment (20 mA) for 90 min forming the final product 2,5-furandicarboxylic acid (FDCA, green line).

References

[1] M. Risch, F. Ringleb, M. Kohlhoff, P. Bogdanoff, P. Chernev, I. Zaharieva, H. Dau, *Energy Environ. Sci.* **2015**, *8*, 661-674.

[2] M. Wilke, G. M. Partzsch, R. Bernhardt, D. Lattard, *Chem. Geol.* **2004**, *213*, 71-87.

[3] a) J. J. Rehr, R. C. Albers, *Rev. Mod. Phys.* **2000**, *72*, 621-654; b) A. L. Ankudinov, B. Ravel, J. J. Rehr, S. D. Conradson, *Phys. Rev. B* **1998**, *58*, 7565-7576.

[4] M. Risch, V. Khare, I. Zaharieva, L. Gerencser, P. Chernev, H. Dau, *J. Am. Chem. Soc.* **2009**, *131*, 6936-6937.

[5] B. Chakraborty, R. Beltran-Suito, J. N. Hausmann, S. Garai, M. Driess, P. W. Menezes, *Adv. Energy Mater.* **2020**, *10*, 2001337.

[6] J. Yang, H. W. Liu, W. N. Martens, R. L. Frost, *J. Phys. Chem. C* **2010**, *114*, 111-119.

[7] P. W. Menezes, A. Indra, D. Gonzalez-Flores, N. R. Sahraie, I. Zaharieva, M. Schwarze, P. Strasser, H. Dau, M. Driess, *ACS Catal.* **2015**, *5*, 2017-2027.

[8] Y. P. Vinichenko, E. N. Sidorova, *J. Phys.: Conference Series* **2016**, *741* 012194.

[9] C. Panda, P. W. Menezes, M. Zheng, S. Orthmann, M. Driess, *ACS Energy Lett.* **2019**, *4*, 747-754.

[10] a) L. Besra, M. Liu, *Prog. Mater. Sci.* **2007**, *52*, 1-61; b) F. Bozza, R. Polini, E. Traversa, *Fuel Cells* **2008**, *8*, 344-350; c) P. W. Menezes, C. Panda, S. Garai, C. Walter, A. Guiet, M. Driess, *Angew. Chem. -Int. Ed.* **2018**, *57*, 15237-15242; d) J. Pfrommer, A. Azarpira, A. Steigert, K. Olech, P. W. Menezes, R. F. Duarte, X. X. Liao, R. G. Wilks, M. Bar, T. Schedel-Niedrig, M. Driess, *ChemCatChem* **2017**, *9*, 672-676.

[11] a) Y. Yang, J. X. Huang, J. Zeng, J. Xiong, J. B. Zhao, *ACS Appl. Mater. Interfaces* **2017**, *9*, 32801-32811; b) Y. Yang, J. Q. Li, D. Q. Chen, T. Fu, D. Sun, J. B. Zhao, *ChemElectroChem* **2016**, *3*, 757-763.

[12] S. Anantharaj, S. R. Ede, K. Karthick, S. S. Sankar, K. Sangeetha, P. E. Karthik, S. Kundu, *Energy Environ. Sci.* **2018**, *11*, 744-771.

[13] a) C. C. L. McCrory, S. Jung, I. M. Ferrer, S. M. Chatman, J. C. Peters, T. F. Jaramillo, *J. Am. Chem. Soc.* **2015**, *137*, 4347-4357; b) C. C. L. McCrory, S. H. Jung, J. C. Peters, T. F. Jaramillo, *J. Am. Chem. Soc.* **2013**, *135*, 16977-16987.

[14] a) L. Negahdar, F. Zeng, S. Palkovits, C. Broicher, R. Palkovits, *ChemElectroChem* **2019**, *6*, 5588-5595; b) A. R. C. Bredar, A. L. Chown, A. R. Burton, B. H. Farnum, *ACS Appl. Energ. Mater.* **2020**, *3*, 66-98.

[15] G. Inzelt, A. Lewenstam, F. Scholzm, *Handbook of Reference Electrodes*, Springer-Verlag Berlin Heidelberg **2013**.

[16] P. W. Menezes, C. Panda, S. Garai, C. Walter, A. Guiet, M. Driess, *Angew. Chem. Int. Ed.* **2018**, *57*, 15237-15242.

[17] S. Loos, I. Zaharieva, P. Chernev, A. Lissner, H. Dau, *ChemSusChem* **2019**, *12*, 1966-1976.

[18] F. Song, M. M. Busch, B. Lassalle-Kaiser, C.-S. Hsu, E. Petkucheva, M. Bensimon, H. M. Chen, C. Corminboeuf, X. Hu, *ACS Cent. Sci.* **2019**, *5*, 558-568.

[19] F. Song, X. Hu, *Nat. Commun.* **2014**, *5* 4477.

[20] J. Jiang, F. Sun, S. Zhou, W. Hu, H. Zhang, J. Dong, Z. Jiang, J. Zhao, J. Li, W. Yan, M. Wang, *Nat. Commun.* **2018**, *9*, 1038.

[21] W. Ma, R. Ma, C. Wang, J. Liang, X. Liu, K. Zhou, T. Sasaki, *ACS Nano* **2015**, *9*, 1977-1984.

[22] B. M. Hunter, J. D. Blakemore, M. Deimund, H. B. Gray, J. R. Winkler, A. M. Muller, *J. Am. Chem. Soc.* **2014**, *136*, 13118-13121.

[23] K. Zhang, W. H. Wang, L. Kuai, B. Y. Geng, *Electrochim. Acta* **2017**, *225*, 303-309.

[24] X. J. Wu, Y. M. Zhao, T. Y. Xing, P. L. Zhang, F. S. Li, H. Lee, F. Li, L. C. Sun, *ChemSusChem* **2018**, *11*, 1761-1767.
[25] Y. H. Liu, Z. Y. Jin, X. Q. Tian, X. Q. Li, Q. Zhao, D. Xiao, *Electrochim. Acta* **2019**, *318*, 695-702.
[26] M. Liu, L. Kong, X. Wang, J. He, X.-H. Bu, *Small* **2019**, *15*, 1903410.
[27] F. G. Chen, L. Y. Zhang, H. Q. Wu, C. Guan, Y. Yang, J. Qiu, P. B. Lyu, M. Li, *Nanotechnology* **2019**, *30*, 32.
[28] X. Teng, L. X. Guo, L. L. Ji, J. Y. Wang, Y. L. Niu, Z. B. Hu, Z. F. Chen, *ACS Appl. Energy Mater.* **2019**, *2*, 5465-5471.
[29] D. Lim, E. Oh, C. Lim, S. E. Shim, S. H. Baeck, *Catal. Today* **2020**, *352*, 27-33.
[30] L. Q. Ji, Y. Kong, C. Wang, H. Tan, H. L. Duan, W. Hu, G. N. Li, Y. Lu, N. Li, Y. Wang, J. Tian, Z. M. Qi, Z. H. Sun, F. C. Hu, W. S. Yan, *ACS Catal.* **2020**, *10*, 5691-5697.
[31] W. Moschkowitsch, K. Dhaka, S. Gonen, R. Attias, Y. Tsur, M. C. Toroker, L. Elbaz, *ACS Catal.* **2020**, *10*, 4879-4887.
[32] M. Zhang, Y. Q. Liu, B. Y. Liu, Z. Chen, H. Xu, K. Yan, *ACS Catal.* **2020**, *10*, 5179-5189.
[33] M. Zhao, H. L. Li, W. Y. Yuan, C. M. Li, *ACS Appl. Energy Mater.* **2020**, *3*, 3966-3977.
[34] Y. Kong, J. Li, Y. Wang, W. Chu, Z. Q. Liu, *Catal. Lett.* **2020**, doi.org/10.1007/s10562-10020-03179-y.
[35] H. T. He, J. Gu, X. M. Liu, D. L. Yang, Y. Zhu, R. Yao, Q. Fan, R. S. Huang, *Catalysts* **2020**, *10*, 431.
[36] Y. Lin, J. L. Wang, D. L. Cao, Y. Q. Gong, *Sustain. Energy Fuels* **2020**, *4*, 1933-1944.
[37] W. J. Zhu, G. X. Zhu, J. Hu, Y. Zhu, H. Chen, C. L. Yao, Z. X. Pi, S. W. Zhu, E. Y. Li, *Inorg. Chem. Comm.* **2020**, *114*, 107851.
[38] R. A. Raimundo, V. D. Silva, E. S. Medeiros, D. A. Macedo, T. A. Simoes, U. U. Gomes, M. A. Morales, R. M. Gomes, *J. Phys. Chem. Solids* **2020**, *139*, 109325.
[39] M. X. Chen, S. L. Lu, X. Z. Fu, J. L. Luo, *Adv. Sci.* **2020**, *7*, 1903777.
[40] S. Si, H. S. Hu, R. J. Liu, Z. X. Xu, C. B. Wang, Y. Y. Feng, *Int. J. Hydrogen Energy* **2020**, *45*, 9368-9379.
[41] S. Liang, B. Wei, M. K. Yuan, Y. Li, X. Ma, Y. Y. Wu, L. L. Xu, *Chemistryselect* **2020**, *5*, 3062-3068.
[42] L. Xu, L. L. Cao, W. Xu, Z. H. Pei, *Appl. Sur. Sci.* **2020**, *503*, 144122.
[43] X. L. Zhang, R. L. Liu, C. Y. Tao, S. S. Wu, F. Huang, H. W. Wang, *J. Alloys Comp.* **2020**, *813*, 152219.
[44] S. Thoufeeq, P. K. Rastogi, S. Thomas, A. Shravani, T. N. Narayanan, M. R. Anantharaman, *Chemistryselect* **2020**, *5*, 1385-1395.
[45] X. Li, M. L. Fan, D. N. Wei, X. L. Wang, Y. L. Wang, *J. Electrochem. Soc.* **2020**, *167*, 037555.
[46] X. Q. Liang, Y. H. Li, H. Fan, S. J. Deng, X. Y. Zhao, M. H. Chen, G. X. Pan, Q. Q. Xiong, X. H. Xia, *Nanotechnology* **2019**, *30*, 484001.
[47] Y. Q. Feng, X. Wang, P. P. Dong, J. Li, L. Feng, J. F. Huang, L. Y. Cao, L. L. Feng, K. Kajiyoshi, C. R. Wang, *Sci Rep* **2019**, *9*, 11.
[48] L. M. Ren, C. Wang, W. Li, R. H. Dong, H. X. Sun, N. Liu, B. Y. Geng, *Electrochim. Acta* **2019**, *318*, 42-50.
[49] M. Zhou, Q. H. Weng, X. Y. Zhang, X. Wang, Y. M. Xue, X. H. Zeng, Y. Bando, D. Golberg, *J. Mater. Chem. A* **2017**, *5*, 4335-4342.
[50] B. Q. Li, S. Y. Zhang, C. Tang, X. Y. Cui, Q. Zhang, *Small* **2017**, *13*, 1700610.

[51] M. Chatti, A. M. Glushenkov, T. Gengenbach, G. P. Knowles, T. C. Mendes, A. V. Ellis, L. Spiccia, R. K. Hocking, A. N. Simonov, *Sustain. Energy Fuels* **2018**, *2*, 1561-1573.
[52] J. H. Yu, G. Z. Cheng, W. Luo, *J. Mater. Chem. A* **2017**, *5*, 15838-15844.
[53] X. Xu, F. Song, X. L. Hu, *Nat. Commun.* **2016**, *7*, 12324.
[54] a) P. W. Menezes, A. Indra, I. Zaharieva, C. Walter, S. Loos, S. Hoffmann, R. Schlögl, H. Dau, M. Driess, *Energy Environ. Sci.* **2018**, *12*, 988-999; b) S. Yao, V. Forstner, P. W. Menezes, C. Panda, S. Mebs, E. M. Zolnhofer, M. E. Miehlich, T. Szilvasi, N. A. Kumar, M. Haumann, K. Meyer, H. Grutzmacher, M. Driess, *Chem. Sci.* **2018**, *9*, 8590-8597.
[55] J. Hu, S. W. Li, J. Y. Chu, S. Q. Niu, J. Wang, Y. C. Du, Z. H. Li, X. J. Han, P. Xu, *ACS Catal.* **2019**, *9*, 10705-10711.
[56] A. A. Lobinsky, V. P. Tolstoy, I. A. Kodinzev, *Nanosystems-Phys. Chem. Maths.* **2018**, *9*, 669-675.
[57] a) S. H. Kang, G. Z. Wang, M. Fang, H. M. Wang, X. B. Wang, W. P. Cai, *J. Mater. Res.* **2015**, *30*, 1629-1638; b) D. M. Cwiertny, G. J. Hunter, J. M. Pettibone, M. M. Scherer, V. H. Grassian, *J. Phys. Chem. C* **2009**, *113*, 2175-2186; c) S. Music, S. Krehula, S. Popovic, *Mater. Lett.* **2004**, *58*, 2640-2645.
[58] J. Osswald, K. T. Fehr, *J. Mater. Sci.* **2006**, *41*, 1335-1339.
[59] C. Feng, M. B. Faheem, J. Fu, Y. Q. Xiao, C. L. Li, Y. B. Li, *ACS Catal.* **2020**, *10*, 4019-4047.
[60] N. S. McIntyre, D. G. Zetaruk, *Anal. Chem.* **1977**, *49*, 1521-1529.
[61] X. M. Wang, M. Q. Zhu, L. K. Koopal, W. Li, W. Q. Xu, F. Liu, J. Zhang, Q. S. Liu, X. H. Feng, D. L. Sparks, *Environ, Sci. Nano* **2016**, *3*, 190-202.
[62] M. Sajid, X. B. Zhao, D. H. Liu, *Green Chem.* **2018**, *20*, 5427-5453.
[63] R. L. Leroy, C. T. Bowen, D. J. Leroy, *J. Electrochem. Soc.* **1980**, *127*, 1954.
